# Keeping it centered: decoding the activities that regulate yeast histone H3 variant Cse4 and confine it to centromeres

**DOI:** 10.3389/fcell.2026.1782273

**Published:** 2026-04-01

**Authors:** Martina Greco, Joanna Klim, Peter De Wulf

**Affiliations:** Department of Cellular, Computational and Integrative Biology (CIBIO), University of Trento, Trento, Italy

**Keywords:** CENP-A, centromere, Cse4, kinetochore, Psh1, Scm3

## Abstract

The field of centromere biology was launched in 1980 with the isolation of a 120-bp centromeric DNA fragment from *Saccharomyces cerevisiae*. Fifteen years later, the discovery that the yeast histone H3 variant Cse4 is the conserved counterpart of human CENP-A established both proteins as the defining epigenetic marks of centromeres. Subsequent genetic screens, biochemical and molecular biology studies have elucidated how Cse4 is specifically targeted to and stably maintained at centromeres. The mislocalization of Cse4 beyond centromeres disrupts transcriptional programs and drives chromosomal instability and aneuploidy. This review traces Cse4 research from its early breakthroughs to current insights into its regulatory pathways. Although derived from yeast, these mechanistic advances provide a conceptual framework for understanding analogous, and likely conserved, processes in humans, where CENP-A biology remains less well defined but is increasingly being implicated in cancer and therapy resistance when perturbed.

## Introduction

**FIGURE 1:**
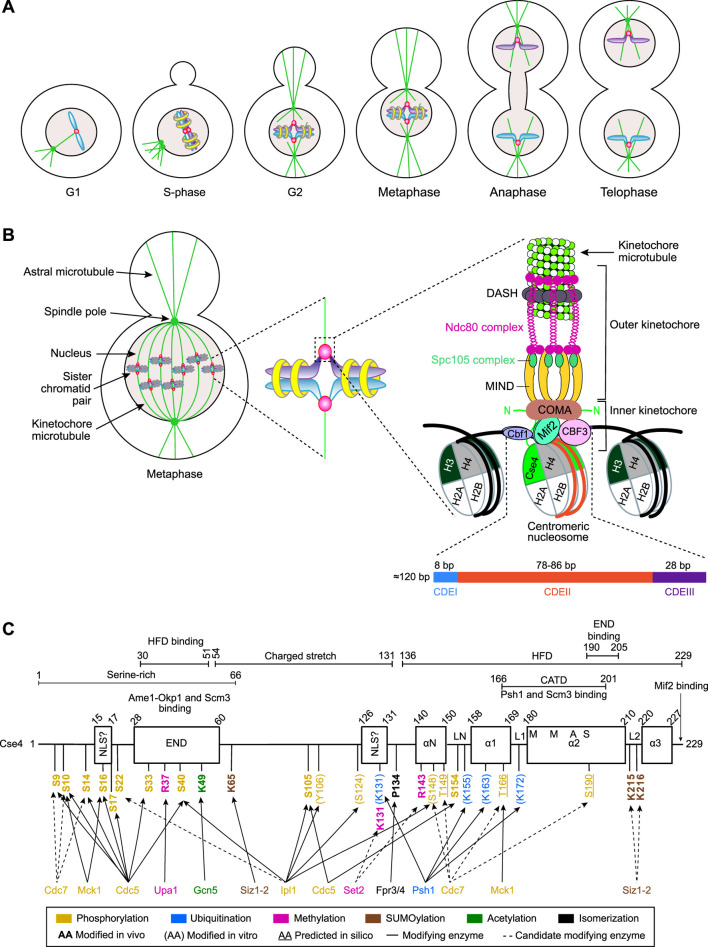
The *Saccharomyces cerevisiae* cell cycle, kinetochore structure, and domain structure of Cse4. **(A)** The *Saccharomyces cerevisiae* cell cycle, showing key stages and corresponding chromosome organization and dynamics. For simplicity, only one chromosome or sister chromatid pair of the 16 in haploid yeast is depicted. **(B)** A simplified schematic of the *Saccharomyces cerevisiae* kinetochore, highlighting the proteins and complexes discussed in this review. **(C)** Domain architecture of Cse4 and its post-translational modifications. Enzymes shown to mediate these modifications *in vitro*, *in vivo*, or as predicted *in silico* are indicated. Abbreviations: NLS, nuclear localization signal; END, essential N-terminal domain; HFD, histone-fold domain; CATD, centromere-associated targeting domain.

Faithful cell division requires precise genome replication followed by accurate chromosome segregation (Figure 1A). This fidelity is enforced by a coordinated network of regulatory proteins. During segregation, centromeres direct kinetochore assembly, enabling replicated chromosomes to attach to the mitotic spindle. Errors in this process lead to aneuploidy, an abnormal number of chromosomes in the daughter cells, which is a hallmark of many cancers and developmental disorders. The budding yeast *Saccharomyces cerevisiae* provides an advantageous model for dissecting these mechanisms, as its core cell cycle machinery is evolutionarily conserved yet relatively simple compared to that of higher eukaryotes. This review examines the histone H3 variant Cse4, the yeast ortholog of human CENP-A, which is essential for centromere identity and kinetochore formation. We highlight major discoveries and outline the regulatory pathways governing Cse4, some of which were identified only recently, and list the corresponding human orthologs in Supplementary Table 1.

### Centromeres, kinetochores, and faithful chromosome segregation

The centromere is a specialized chromosomal locus on which kinetochores assemble after DNA replication. The duplicated chromosomes, called sister chromatids, are paired by cohesin rings (Figure 1B). Next, each centromere directs the formation of a kinetochore, an evolutionarily conserved multi-protein complex comprising more than 60 subunits in *S. cerevisiae* and more than 100 in humans. Its main components are conserved across eukaryotes (Drinnenberg et al., 2016; Musacchio and Desai, 2017; van Hooff et al., 2017). Two kinetochores attach their underlying sister chromatid pair to microtubules emanating from opposite spindle poles (Figure 1A). Once all sister chromatids have achieved bipolar attachment to the mitotic spindle, cohesin cleavage triggers chromatid separation. Microtubule depolymerization drives their equal segregation between the mother and daughter cells. The various stages of the yeast cell division process and associated chromosome states (as represented by one chromosome or sister chromatid pair) are shown in Figure 1A.

Centromere sequences exhibit extensive diversity across eukaryotes. In *S. cerevisiae*, the short “point centromeres” of approximately 120 bp contain three defined sequence elements (CDEI–III) (Figure 1B). By contrast, human “regional centromeres” span ∼340 kb to 4.8 Mb and comprise ∼171 bp alpha-satellite repeats that are organized into higher-order arrays. Such variability indicates that DNA sequence alone does not specify centromere identity. Instead, incorporation of a histone H3 variant (Cse4 in *S. cerevisiae* and CENP-A in humans) into centromeric nucleosomes marks the locus and directs kinetochore assembly. This deposition occurs within a restricted cell cycle window (early S-phase in budding yeast and G1 in humans). Mislocalization of Cse4 or CENP-A can generate neocentromeres that are potentially capable of recruiting kinetochores (Scott and Sullivan, 2014). The presence of multiple kinetochores bound to a chromosome produces antagonistic spindle forces that promote chromosome missegregation, breakage, and large-scale genomic rearrangements, thereby driving genomic instability. Aberrant Cse4/CENP-A deposition alters transcriptional programs, consequently compromising cellular fitness. Notably, CENP-A overexpression and mislocalization are frequently observed in human cancers and may contribute to tumor development and progression (Sun et al., 2016; Shrestha et al., 2017; Mahlke and Nechemia-Arbely, 2020; Jeffery et al., 2021; Renaud-Pageot et al., 2022).

As shown in Figure 1B for *S. cerevisiae*, the kinetochore comprises two functional protein layers: an inner layer that binds centromeric DNA and an outer layer that mediates attachment to spindle microtubules. Kinetochores also serve as the platform for the spindle assembly checkpoint (SAC), which verifies the proper bi-orientation of each sister chromatid pair on the mitotic spindle. Dynamic microtubule forces act on each kinetochore pair, while the cohesin-mediated binding of both sister centromeres resists these forces, generating intrakinetochore and intercentromeric tensions that are monitored by the SAC. Incorrect or absent microtubule attachments fail to produce these tensions, activating the SAC to delay anaphase onset until all sister chromatids have achieved bi-orientation. The failure of even a single chromatid pair to satisfy these requirements induces a SAC-dependent arrest of cell division at the metaphase–anaphase transition and can lead to apoptotic cell death. This surveillance mechanism prevents propagation of cells with abnormal chromosome numbers (Lara-Gonzalez et al., 2021; McAinsh and Kops, 2023; Pun and North, 2024).

### Yeast centromeres harbor a Cse4-containing octameric nucleosome

The *S. cerevisiae* chromosome III centromere (CEN3), characterized 45 years ago (Clarke and Carbon, 1980), spans ∼120 bp of DNA and contains three conserved DNA elements: the A + T-rich CDEII (78–86 bp) flanked by CDEI (8 bp) and CDEIII, a 28-bp sequence with partial dyad symmetry. Mutations in CDEIII severely impair centromere function (Ng et al., 1986; Fitzgerald-Hayes, 1987; Saunders et al., 1988; Baker and Rogers, 2005), whereas mutations in CDEI and CDEII permit chromosome maintenance. DNA wrapping within the centromeric nucleosome juxtaposes CDEI and CDEIII on one face while allowing the flexible CDEII region to engage the histone core. This configuration provides a platform for kinetochore assembly, creates a stable attachment site that is resistant to spindle tension during sister chromatid bi-orientation, and insulates the centromere from transcription activity (Hill and Bloom, 1987). Affinity purifications of centromeric DNA identified centromere-binding factors. Cbf1 binds CDEI (Bram and Kornberg, 1987; Baker et al., 1989; Jiang and Philippsen, 1989; Baker and Masison, 1990; Cai and Davis, 1990) as a homodimer and functions as a transcriptional repressor (Hemmerich et al., 2000; Chen et al., 2019; Hedouin et al., 2022; Smurova et al., 2023). The four-protein CBF3 complex (Ndc10 (Cbf2), Cep3 (Cbf3), Ctf13, and Skp1) (Lechner and Carbon, 1991; Leber et al., 2018) binds CDEIII and recruits all other kinetochore subunits, including Cse4 (Bouck D. and Bloom K., 2005; Yan et al., 2018; Guan et al., 2021; Dendooven et al., 2023) and Mif2, which interacts with CDEII through its AT-hook motif (Brown, 1995; Meluh and Koshland, 1995) (Figure 1B).

Canonical nucleosomes comprise two copies each of histones H2A, H2B, H3, and H4. By contrast, centromeric nucleosomes in yeast and humans substitute histone H3 with Cse4 and CENP-A, respectively (De Rop et al., 2012; Muller and Almouzni, 2014; McAinsh and Marston, 2022) (Figure 1B). Human centromeres contain alternating blocks of CENP-A and H3-containing nucleosomes across large alpha-satellite arrays that fold into a higher-order structure, which attaches up to 30 microtubules to each centromere. By comparison, *S. cerevisiae* centromeres comprise a single Cse4-containing nucleosome that anchors one microtubule (Fitzgerald-Hayes et al., 1982; Winey et al., 1995) (Figure 1B). Conventional anti-Cse4 chromatin immunoprecipitation (ChIP) could not determine the number or positioning of Cse4 nucleosomes relative to the centromere. However, combining anti-Cse4 ChIP experiments with MNase digestion and Southern blotting demonstrated that Cse4 is restricted to the centromere and that a single, Cse4-containing nucleosome constitutes the minimal chromatin unit required for kinetochore assembly and faithful chromosome segregation (Furuyama and Biggins, 2007).

Cryo-electron microscopy of soluble Cse4 nucleosomes assembled *in vitro* from co-expressed *S. cerevisiae* histone proteins H2A, H2B, and Cse4, and *Kluyveromyces lactis* H4, together with 3D reconstruction and density modeling, showed that the octamer wraps a shorter stretch of DNA (119 bp) than a canonical yeast nucleosome (146 bp) (White et al., 2001; Migl et al., 2020), which is consistent with MNase protection assays of *in vitro* reconstituted Cse4 nucleosomes (Kingston et al., 2011). Structural studies also revealed that the last three C-terminal residues of Cse4 form a hydrophobic patch that interacts with the centromere-binding protein Mif2 (Kato et al., 2013), while the exposed L1 loop binds the N-terminal tail of Chl4. This kinetochore protein contacts the DNA. The loose N-terminal tail of Cse4 mediates binding to the inner kinetochore heterotetrameric COMA complex (Ctf19, Okp1, Mcm21, and Ame1) (De Wulf et al., 2003), further promoting centromere recognition during kinetochore assembly (Anedchenko et al., 2019; Hinshaw and Harrison, 2019; Deng et al., 2023) (Figures 1B,C).

### Two roads lead to Cse4

In a seminal study, Stoler et al. (1995) developed a genetic screen in *S. cerevisiae* to identify factors required for faithful chromosome segregation. The screen exploited a disomic haploid strain carrying an engineered, mitotically unstable chromosome III with a defective centromere containing a 47-bp insertion in CDEII. This chromosome harbored the selectable marker *URA3* and *SUP11*, which encodes a suppressor tyrosyl-tRNA. Colony color served as a sensitive readout of chromosome segregation fidelity. The host strain carried the *ade2-101* mutation, which results in red pigmentation due to accumulation of an adenine biosynthetic intermediate; expression of *SUP11* suppresses this phenotype by promoting translational read-through of the premature stop codon, restoring Ade2 activity and yielding white colonies. As a result, chromosome copy number could be inferred from colony color using a sectoring assay. Single-chromosome loss produced red or pink sectors (1:0 segregation), whereas nondisjunction resulted in red-and-white sectoring (2:0 segregation). Mutagenesis of this reporter strain with ethyl methanesulfonate enabled the isolation of mutants exhibiting increased chromosome missegregation, which were subsequently screened for temperature-sensitive growth defects. One mutant, named *cse4-1* (defective in chromosome segregation), displayed a 17-fold increase in missegregation of the *cen130-3* chromosome. The mutation was recessive, as *CSE4/cse4-1* heterozygotes exhibited normal chromosome segregation. At 38 °C, haploid *cse4-1* cells arrested as large-budded G2/M cells with 2N DNA content and short bipolar spindles, consistent with a chromosome segregation defect rather than impaired DNA replication. Complementation with a genomic library identified ORF YKL262 as the affected gene. Next, YKL262 deletion lethality was rescued by plasmid-borne *CSE4*, demonstrating that *CSE4* activity is essential for viability (Stoler et al., 1995). The *cse4-1* mutation was later identified as an A61T substitution (Stoler et al., 2007).

In 1996, the *S. cerevisiae* histone H4-producing gene *HHF1* (one H4-encoding gene in addition to *HHF2*) was cloned into a plasmid, mutagenized *in vitro* using sodium bisulfite, and expressed as the sole H4-producing gene in yeast (Smith et al., 1996). Two temperature-sensitive *hhf1* mutants arrested at G2/M as large-budded cells with a single nucleus and short bipolar spindle, indicative of a mitotic defect. Functional analyses of both mutants yielded a composite allele, *hhf1-20*, that carried two substitutions: T82I, which was lethal due to disrupted H4–DNA binding, and A89V, which partially suppressed T82I at 28 °C. At this temperature, *hhf1-20* cells exhibited a two-fold increase in chromosome missegregation (rising to 50-fold at 35 °C) and were inviable at 37 °C. A dosage suppressor screen of the *hhf1-20* allele identified *HHF1*, *HHF2,* and *CSE4* (Stoler et al., 1995) but not the histone H3-encoding *HHT1* and *HHT2*, indicating a specific functional interaction between H4 and Cse4. The authors proposed that at the restrictive temperature, the protein encoded by *hhf1-20* failed to interact with Cse4 and the centromeric DNA, thereby impairing kinetochore function. Overexpressing *CSE4* in the *hhf1-20* mutant restored growth at 37 °C by promoting functional Cse4–Hhf1-20 dimer formation. ChIP experiments and immunofluorescence imaging localized Cse4 to centromeres (Meluh et al., 1998), likely via direct binding, given its homology to the DNA-binding histone protein H3 (Luger et al., 1997), establishing Cse4 as an H4 partner and essential centromeric histone variant.

### The sequence determinants of Cse4 activity

Cse4 is a 229-amino acid protein consisting of an N-terminal tail (131 residues) and a C-terminal histone-fold domain (HFD, 94 residues) connected by a short linker (residues 131–136) (Figure 1C). The N-terminal region contains a putative bipartite nuclear localization signal (residues 15–17 and 126–131), a serine-rich segment (residues 1–66), and a highly charged region (residues 54–131) enriched in arginine and glutamic acid. Unlike histone H3, this N-terminal tail does not bind DNA and instead projects outward from the centromeric nucleosome to support kinetochore-specific functions (Meluh et al., 1998; Keith et al., 1999). Within this region, a 33-residue essential N-terminal domain (END; residues 28–60) is required for chromosome segregation and viability (Keith et al., 1999; Chen et al., 2000). Interestingly, residues 34–46 of the END domain adopt an ordered alpha-helical structure upon binding to the Okp1–Ame1 complex (Deng et al., 2023). The END domain is absent from histone H3 and human CENP-A (Collins et al., 2004), explaining their inability to rescue a *cse4Δ* strain (Stoler et al., 1995). Notably, yeast Cse4 can localize to kinetochores in CENP-A-depleted human cells, indicating functional conservation (Wieland et al., 2004). The methionine at position 93 of Cse4 suggests an alternative translation start site that would produce an H3-length protein, supporting the hypothesis that Cse4 arose from a gene fusion in which H3 acquired the END domain. In contrast to Cse4, the N-terminal tail of histone H3 binds DNA and is extensively modified to regulate transcription (Mann and Grunstein, 1992; Santos-Rosa et al., 2009; Sperling and Grunstein, 2009; Singh et al., 2021; Deshpande et al., 2022). In contrast, the CENP-A N-terminus does not bind DNA but stabilizes centromere-binding proteins such as CENP-B (which has no clear *S. cerevisiae* ortholog) and CENP-C (the *S. cerevisiae* Mif2 ortholog), which are essential for kinetochore assembly in humans (Fachinetti et al., 2015; Ali-Ahmad et al., 2019).

Alanine-scanning mutagenesis of the Cse4 END domain identified key residues (D36, R37, R44, R46, and K49) required for function (Chen et al., 2000). Mutations at these positions; singly or combined, caused temperature sensitivity, slow growth, and synthetic lethality with mutations in CBF3 or COMA components, as well as Cbf1 or Mcm22 (a subunit of the inner kinetochore Mcm22–Mcm16–Ctf3 complex (Sanyal et al., 1998; Measday et al., 2002)), highlighting the essential role of the END sequence in Cse4 and kinetochore function (Chen et al., 2000). The END domain was later shown to interact with and stabilize COMA subunits Okp1–Ame1 and the Cse4 chaperone Scm3 (Ortiz et al., 1999; Anedchenko et al., 2019; Shukla et al., 2024; Popchock et al., 2025) (Figure 1C). In contrast, the region between the END domain and the linker preceding the HFD (residues 60–131) is dispensable for Cse4 function (Chen et al., 2000).

The Cse4 histone-fold domain (residues 136–229) shares 64% identity with the HFDs of H3 and human CENP-A (Chen et al., 2000), comprises a central α-helix (α2) flanked by three shorter α-helices (αN, α1, and α3) that are separated by β-strands (LN, L1, and L2) (Figure 1C). This domain is essential for centromere targeting of Cse4 in *S. cerevisiae* (Morey et al., 2004). Chimeric proteins containing the Cse4 N-terminus fused to the HFD of H3 or CENP-A failed to rescue *cse4Δ*, demonstrating that the Cse4 HFD confers centromere specificity. Mutational analyses further showed that this function did not depend on a single amino acid or short internal segment but rather on a combination of distributed residues (Keith et al., 1999). Notably, only one functional END domain and one functional HFD within a centromeric Cse4 dimer are sufficient to support nucleosome assembly and kinetochore formation (Chen et al., 2000).

### Cse4 expression and deposition at centromeres

#### Regulation of *CSE4* expression

Although Cse4 has been extensively characterized at the protein level, including its modifications, turnover, centromere recruitment, and cell cycle dynamics, its transcriptional regulation remains poorly understood. A genome-wide study revealed that *CSE4* transcript levels fluctuate periodically during the cell cycle but did not meet the stringent criteria for inclusion as a high-confidence cell cycle-regulated gene. Nevertheless, the data provided strong evidence for temporal regulation, with *CSE4* mRNA abundance peaking during S-phase, consistent with its role in guiding kinetochore assembly immediately following centromere replication (Spellman et al., 1998). To date, *CSE4* expression has been linked to two transcription factors. Cse4 promoter binds Stp1 (Venters et al., 2011), a downstream effector of the Ssy1–Ptr3–Ssy5 amino acid-sensing pathway. Upon detection of extracellular amino acids, proteolytic activation of Stp1 by Ssy5 enables its nuclear entry and transcriptional activation of permease-encoding genes involved in peptide and amino acid uptake, suggesting that Stp1 may upregulate *CSE4* under nutrient-rich conditions to support cell growth and proliferation. To date, no human orthologs of Stp1 have been identified (Supplementary Table 1). *CSE4* transcription is also regulated by Sfp1 (Cipollina et al., 2008), a nutrient-responsive factor that promotes the expression of ribosomal proteins and ribosome biogenesis genes under favorable conditions (Marion et al., 2004). Under nutrient limitation, Sfp1 relocalizes to the cytoplasm, leading to reduced transcription and slowed growth. Sfp1 additionally controls cell cycle regulators, including *CLN1* and *CLN2* at the G1/S transition, *PDS1* at G2/M, and modulates DNA damage responses, linking growth signals to cell division (Xu and Norris, 1998; Albert et al., 2019). Based on these activities, Sfp1 has been proposed as a functional ortholog of the human transcription factor c-Myc (Lempiainen et al., 2009).

#### Cse4 is deposited at centromeres in the early S-phase

**FIGURE 2:**
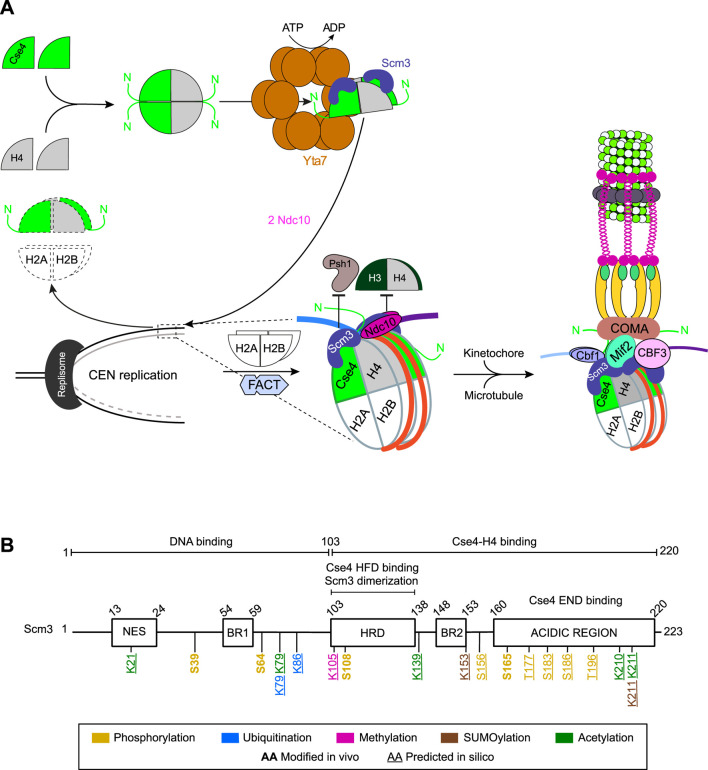
Cse4–H4 deposition at centromeres and the domain structure of Scm3. **(A)** Pathway for Cse4 nucleosome formation, centromere deposition, and subsequent kinetochore assembly during early S-phase. **(B)** Domain architecture of Scm3 and its post-translational modifications. The enzymes responsible for mediating these modifications *in vitro*, *in vivo*, or as predicted *in silico* remain unknown. NES, nuclear export signal; BR, basic-residue patch; HRD, heptad repeat domain; END, essential N-terminal domain. HFD, histone-fold domain.

While Cse4 was known to be present in two Cse4–H4 dimers per centromere, the timing and stability of Cse4 incorporation during the cell cycle were unclear. FRAP analysis of Cse4–GFP revealed low fluorescence recovery (∼18%) at centromeres in G1, G2/M, metaphase, anaphase, and telophase, while substantially higher recovery (∼63%) was observed in early S-phase, coincident with centromere replication. These results indicated that Cse4 exchange occurs specifically during centromere replication, after which the newly deposited Cse4–H4 dimers are stably retained (Figure 2A). During the deposition process, sister kinetochores transiently detach from spindle microtubules and are randomly recaptured following centromeric nucleosome reassembly (Pearson et al., 2004) (Figure 2A). These findings were corroborated using a strain expressing endogenously tagged, photoconvertible Cse4–tdEos–Cse4 (Wisniewski et al., 2014), whose fluorophore irreversibly converts from a fluorescent green to red state upon exposure to violet light. Live-cell imaging showed that red-labeled centromeric Cse4–tdEos–Cse4 in G1 cells was replaced by newly synthesized green Cse4–tdEos–Cse4 in early S-phase, which then persisted through mitosis and the subsequent G1. A second round of photoconversion in the following cell cycle confirmed the incorporation of newly synthesized green Cse4–tdEos–Cse4 exclusively in early S-phase. Consistently, FRAP analysis of mitotic cells detected no Cse4–tdEos–Cse4 exchange. Together, these data demonstrate that Cse4–H4 deposition is tightly restricted to early S-phase and that incorporated dimers remain stably associated with centromeres throughout the cell cycle.

#### Centromeric Cse4 is bound to hypoacetylated histone H4

The N-terminal tail of yeast histone H4 contains four conserved lysines (K5, K8, K12, and K16) that undergo regulated acetylation to control chromatin dynamics and gene expression (Dion et al., 2005). Among these, K16 acetylation is particularly important for chromatin compaction and the maintenance of silent chromatin domains. H4K16 acetylation levels are controlled by the opposing activities of the acetyltransferase Sas2 and the deacetylase Sir2 (Suka et al., 2002; Shia et al., 2006; Grunstein and Gasser, 2013).

**FIGURE 3:**
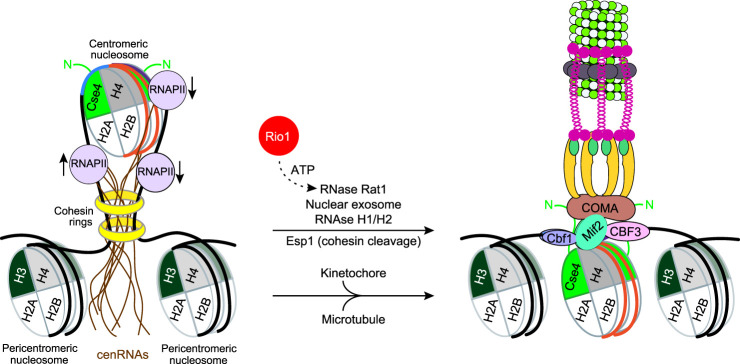
Role of C-loop formation in kinetochore assembly and its dissolution at mitosis. Model depicting how centromeric transcripts (cenRNAs) promote the formation and stabilization of the centromeric C-loop. CenRNAs, produced by RNA pol II from pericentromeric regions, intertwine with centromere-flanking chromatin. This association is strengthened and stabilized by the local enrichment of cohesin complexes. The resulting C-loop configuration positions the centromeric nucleosome at its apex, facilitating kinetochore assembly and enabling sister chromatid bi-orientation on the spindle. At mitotic entry, the C-loop is dissolved through the degradation of cenRNAs by the nuclear exosome, RNase Rat1, and RNases H1/H2 and the cleavage of cohesin by the protease Esp1. The Rio1 kinase promotes Rat1 activity, acting in parallel with the exosome and RNases H1/H2.

ChIP analyses revealed that, when associated with Cse4, centromeric H4 is hypoacetylated, especially at K16. Expression of an acetyl-mimetic H4K16Q or increasing the acetylation of wild-type H4 by *SAS2* overexpression, *SIR2* deletion or inactivation, or nicotinamide treatment (inhibits histone deacetylation) caused increased chromosome loss and reduced viability of *cse4* and *hhf1* mutants that are defective in centromere targeting (*cse4-1*) or in the Cse4–H4 interaction (*hhf1-20*) (Choy et al., 2011). These results suggested that the Cse4–H4 interaction protects H4 from acetylation. Consistent with this model, H4 hypoacetylation promotes the formation of the ∼50 kb pericentromeric chromatin loop (C-loop) (Choy et al., 2012) that positions the Cse4 nucleosome at its apex (Figure 3), a configuration that is required for proper sister chromatid bi-orientation (Yeh et al., 2008; Stephens et al., 2011; Lawrimore and Bloom, 2019).

#### Histone H4 binding converts closed Cse4 into an open state

Newly synthesized Cse4 adopts a “closed” conformation in which its END domain (specifically, residues 30–51) interacts with the α2 helix in the HFD (residues 190–205) (Figure 1C). This closed state may protect Cse4 from degradation, prevent premature interactions with kinetochore components, and restrict its incorporation at centromeric and non-centromeric regions. Binding to histone H4 disrupts these intramolecular contacts, converting Cse4 to an “open” conformation that exposes its N-terminal tail (Malik et al., 2018) (Figure 2A). Deletion of either *HHF1* or *HHF2* markedly reduced the open Cse4 population, further confirming that histone H4 drives the closed-to-open transition of Cse4 in cells (Ohkuni et al., 2024). However, the role of histone H4 acetylation in this process remains unclear. Only the open Cse4–H4 dimers are competent for centromeric incorporation (Malik et al., 2018; Mishra et al., 2018; Fischbock-Halwachs et al., 2019; Deng et al., 2023; Popchock et al., 2025) (Figure 2A). In addition, the Cse4–H4 interaction promotes the exposure of the Cse4 C-terminal tail to SUMOylation, which contributes to its non-centromeric localization (Eisenstatt et al., 2021; Ohkuni et al., 2022).

#### Scm3 deposits Cse4–H4 at centromeres

Genetic screens for dosage suppressors of temperature-sensitive *cse4* alleles mutated in their HFD identified two genes: *CSE4* itself and the essential ORF YDL139C, which was later named suppressor of chromosome missegregation 3 (*SCM3*). *SCM3* was not recovered in dosage suppressor screens of *cse4* END-domain mutants (Stoler et al., 2007), indicating a specific, functional interaction between Scm3 and the HFD of Cse4. Scm3 was also shown to physically interact with Cse4 (Camahort et al., 2007; Mizuguchi et al., 2007), to form homodimers (Stoler et al., 2007) and to associate with the centromere-binding CBF3 subunit Ndc10. The localization of Scm3 and Cse4 to centromeres is mutually dependent. In contrast, Ndc10 does not directly interact with Cse4 (Camahort et al., 2007).

Scm3 is a conserved 223-amino acid protein, a functional ortholog of human HJURP (Sanchez-Pulido et al., 2009) that deposits CENP-A at centromeres during G1 (Dunleavy et al., 2009). Structural and functional analyses have identified several key domains within Scm3 (Figure 2B). The N-terminal region (residues 1–25) is essential for viability and contains a functional nuclear export signal (residues 13–24), whereas the function of residues 1–13 remains unknown (Shivaraju et al., 2011). Scm3 also contains two nonessential basic-residue patches (BR1: residues 54–59; BR2: residues 148–153) that resemble but do not function as nuclear localization signals, implying that its nuclear import depends on another internal sequence or interaction with a partner protein such as Cse4. The C-terminal half of Scm3 comprises an essential globular domain formed by five tandem heptad repeats (HRD (heptad repeat domain): residues 103–138), BR2, and an acidic stretch (residues 160–220). It mediates Cse4–H4 binding and H2A–H2B displacement (Mizuguchi et al., 2007; Stoler et al., 2007; Shivaraju et al., 2011). The HR domain, which promotes Scm3 dimerization, directly binds to the HFD of Cse4 and constitutes the primary Cse4-binding site. Mutations in this region disrupt Cse4 localization to centromeres, underscoring its essential role in the centromeric targeting of Cse4 (Stoler et al., 2007; Shivaraju and Gerton, 2011; Shivaraju et al., 2011; Xiao et al., 2011).

Newly synthesized Scm3 is intrinsically disordered (Shukla et al., 2024) but undergoes partial helical folding (Zhou et al., 2011) upon binding the Cse4 CATD region, which directs the centromeric targeting of the (Cse4–H4)_2_ complex (Cho and Harrison, 2011; Dechassa et al., 2011; Xiao et al., 2011; Zhou et al., 2011; Wisniewski et al., 2014). Scm3 also interacts weakly via its C-terminal acidic region (residues 160–220; Figure 2B) with the END domain of Cse4. However, this interaction neither induces Scm3 folding nor forms a stable Scm3–Cse4 complex, as the Cse4 N-terminus disrupts Scm3’s internal contacts. Through its N-terminal residues 1–103 (Mizuguchi et al., 2007), Scm3 binds centromeric DNA with higher affinity than the Cse4 N-terminus. Centromeric recruitment of (Cse4–H4)_2_ by two Scm3 molecules is mediated primarily by the Scm3 HR domain and the Cse4 HFD, after which DNA wrapping likely displaces Scm3 from the Cse4 HFD to the END domain (Wisniewski et al., 2014).

*In vitro* assays identified Scm3 as a Cse4-specific chaperone that binds the (Cse4–H4)_2_(H2A–H2B)_2_ octamer but not its H3-containing counterpart, as Scm3 pulled down Cse4–H4 but not H2A–H2B (Shivaraju et al., 2011). Scm3 binding to Cse4-containing octamers displaces H2A–H2B dimers, consistent with reduced H2A–H2B occupancy at Scm3-bound centromeres, as observed by chromatin immunoprecipitation (ChIP) sequencing (Mizuguchi et al., 2007).

These data support a model in which two Scm3:Cse4–H4 complexes associate with centromeric DNA, followed by (H2A–H2B)_2_ incorporation to complete nucleosome assembly (Figure 2B). This process requires the Cse4 centromere-associated targeting domain (CATD; residues 166–201) within its HFD (Shivaraju et al., 2011) (Figure 1C). Although Scm3 lacking its HR domain cannot bind Cse4 and its CATD, Scm3 can still bind centromeric DNA via its N-terminal region (residues 1–103) independently of Cse4 (Mishra et al., 2011). Notably, centromeric DNA inhibits Cse4 nucleosome assembly *in vitro* unless Scm3 is present, underscoring Scm3’s essential chaperone function in (Cse4–H4)_2_ deposition at centromeres (Xiao et al., 2011).

#### Ndc10 cooperates with Scm3

Centromeric DNA is an intrinsically poor template for Cse4 nucleosome assembly *in vitro* (Drew and Travers, 1985; Meluh et al., 1998; Widom, 2001; Mizuguchi et al., 2007). *In vivo*, DNA-binding factors Cbf1 and Ndc10 (CBF3 complex) bind CDEI and CDEIII, respectively, thereby positioning two Scm3:(Cse4–H4) dimers at CDEII through an Scm3–Ndc10 interaction. Each Scm3 engages one end of CDEII and dimerizes (Xiao et al., 2011) (Figure 2A). Cse4 stabilizes the Scm3–Ndc10 interaction, indicative of cooperative kinetochore assembly (Camahort et al., 2007) (Figure 2A). Subsequent histone-DNA interactions and formation of the Cse4:Cse4 interface reposition Scm3 from the Cse4 HFD to its N-terminus within the centromere-bound (Cse4–H4)_2_ complex. This shift of Scm3 frees the Cse4 HFD for (H2A–H2B)_2_ incorporation and completion of the centromeric nucleosome. Consistently, deleting CDEIII, which abolishes Ndc10/CBF3 binding, leads to increased (H3–H4)_2_ incorporation, suggesting that CDEIII–CBF3 prevents (H3–H4)_2_ loading at centromeres (Popchock et al., 2023) (Figure 2A). Once assembled, Cse4, Ndc10, and Scm3 remain associated with centromeric chromatin throughout the cell cycle (Camahort et al., 2007; Mizuguchi et al., 2007; Stoler et al., 2007; Cho and Harrison, 2011; Xiao et al., 2011).

#### Scm3 protects centromeric and soluble Cse4 from degradation

Intracellular Scm3 levels remain constant across the cell cycle, but the centromeric association is dynamic: it peaks in S-phase, declines through metaphase, doubles in anaphase, and returns to baseline at mitotic exit (Luconi et al., 2011; Mishra et al., 2011). The transient anaphase enrichment likely stabilizes Cse4, as Cse4 is lost from centromeres in the anaphase-arrested *scm3-1* mutant (Luconi et al., 2011), indicating that Scm3 acts both as a Cse4 loader and protector. Conditional Scm3 depletion triggers a SAC-dependent G2/M arrest and reduces the centromeric levels of Cse4, Ndc10/CBF3, Mif2, and Cbf1 (Camahort et al., 2007; Mizuguchi et al., 2007; Stoler et al., 2007). In contrast, Scm3 overexpression increases its own centromeric occupancy but markedly (three-fold) decreases centromeric Cse4 and H4 levels, leading to a 10-fold increase in chromosome loss and modestly increased soluble Cse4. Restoring Cse4 or H4 expression rescues centromeric Cse4 levels and genomic stability, supporting a role for Scm3 in protecting non-chromatin-bound Cse4 from degradation (Mishra et al., 2011).

Proteomic analyses have identified phosphorylation of Scm3 at S39, S64, S108, and S165 (Engel et al., 2025; UniProt, 2025) (Figure 2B), although their functional relevance and responsible enzymes remain unknown. *In silico* analyses further predict additional post-translational modifications, including phosphorylation (S156, T177, S183, S186, and T196), acetylation (K21, K79, K139, K210, and K211), methylation (K105), ubiquitination (K79 and K86), and SUMOylation (K153 and K211) (Shrestha et al., 2024). Collectively, these findings suggest previously unrecognized regulatory mechanisms governing Scm3 localization and function at centromeres.

#### Yta7 unfolds (Cse4–H4)_2_ tetramers and delivers the Cse4–H4 dimers to Scm3

Genetic suppressor screens using the kinetochore mutant *cse4-R37A okp1-5*, which exhibits a synthetic growth defect, identified one mutation in the E3 ubiquitin ligase Ubr2 and multiple mutations in the gene *YTA7* (yeast tat-binding analog 7). Although deleting *YTA7* alone did not affect cell viability or chromosome loss, it often impaired growth when combined with other kinetochore mutations. However, *yta7Δ* rescued the temperature sensitivity of *okp1-5*, implicating Yta7 in kinetochore assembly and/or function (Shahnejat-Bushehri and Ehrenhofer-Murray, 2020).

Yta7 is a large (1,379 amino acids) conserved AAA^+^-ATPase and ATP-dependent histone chaperone (Cattaneo et al., 2014) that assumes a double-stacked hexameric ring structure (Figure 2A) (Wang et al., 2022) similar to its fission yeast ortholog Abo1 (Cho et al., 2019). ATAD2, the human ortholog of Yta7 (Supplementary Table 1), is frequently overexpressed in cancer and associates with poor prognosis (Boussouar et al., 2013). Yta7 regulates gene expression by promoting nucleosome assembly and disassembly during transcription, in part through interaction with the FACT (facilitates chromatin transcription) complex subunit Spt16 to remodel H2A–H2B during transcription elongation (Gradolatto et al., 2008; Gurova et al., 2018). Yta7 binds all core histones and variant H2A.Z (Tackett et al., 2005; Kurat et al., 2011), independently of their acetylation state. Despite its roles in gene regulation, deleting *YTA7* does not alter kinetochore gene expression (Lombardi et al., 2011) or centromere transcription (Shahnejat-Bushehri and Ehrenhofer-Murray, 2020).

ChIP analyses showed that Yta7 localizes to centromeres, and its deletion reduces centromeric Cse4 levels without increasing histone H3 occupancy, indicating that Cse4 loss is not due to local H3 replacement. Yta7 interacts with Cse4 *in vivo*, and its overexpression increases centromeric Cse4 in an Scm3-dependent manner, suggesting that Yta7 functions as a Cse4 chaperone, facilitating its incorporation into centromeric nucleosomes. Genetic and biochemical evidence, including co-immunoprecipitation and the absence of additive growth defects in *yta7Δ scm3-1* mutants, supports the explanation that Yta7 and Scm3 act in the same pathway. These findings suggest that Yta7 facilitates Cse4 incorporation by unfolding (Cse4–H4)_2_ tetramers and favoring their transfer to Scm3, thereby regulating the availability of (Cse4–H4)_2_ heterodimers for centromeric nucleosome assembly (Figure 2A) (Shahnejat-Bushehri and Ehrenhofer-Murray, 2020). Whether the human ortholog ATAD2 plays a similar role in CENP-A deposition remains unknown.

#### Scm3 and Okp1–Ame1 stabilize Cse4 at centromeres

Reconstitution experiments demonstrated that the Cse4 END domain binds the Okp1–Ame1 dimer of the COMA complex (Anedchenko et al., 2019; Fischbock-Halwachs et al., 2019; Hinshaw and Harrison, 2019). Binding assays further revealed a ternary complex involving the Cse4 N-terminus, Scm3, and Okp1–Ame1, indicating cooperative interactions (Shukla et al., 2024). Consistent with this, depleting Okp1 reduced the centromeric Cse4 levels by 56%, indicating a role of Okp1–Ame1 in stabilizing deposited Cse4 (Popchock et al., 2025). Disruption of the Cse4 END: Okp1–Ame1 interaction is lethal when the integrity of COMA or associated proteins (e.g., the Chl4–Iml3 complex) is compromised (Anedchenko et al., 2019), establishing the Cse4 END:Scm3–Okp1–Ame1 axis as a critical linker between the Cse4 nucleosome and the spindle microtubule (Figure 2A).

Single-molecule total internal reflection fluorescence microscopy (TIRFM) revealed that deletion of the Cse4 END domain reduces centromere binding 88% relative to wild-type (Popchock et al., 2025). Likewise, END mutations that disrupt the Cse4:Okp1–Ame1 interaction reduce centromeric Cse4 levels by more than 50% despite normal Scm3 and Ndc10. An Ame1 mutant defective in END binding similarly impaired the association of Cse4 with centromeres (Deng et al., 2023). Together, these findings establish the Cse4 END domain and its interaction with Okp1–Ame1 as essential for maintaining Cse4 at centromeres after deposition.

### Post-translational modifications of Cse4

#### Methylation

Endogenous Cse4 migrates ∼10 kDa above its predicted molecular weight on SDS-PAGE (Stoler et al., 1995; Keith et al., 1999), indicating post-translational modifications. Mass spectrometry of yeast-purified Cse4 confirmed methylation at R37 within the END domain (Figure 1C). Although the nonmethylatable Cse4-R37A mutant was phenotypically normal, it exhibited synthetic lethality, G2/M arrest, and chromosome segregation defects when combined with *cbf1Δ* or mutations in other kinetochore components, including the COMA subunit Ctf19 and the MIND complex. Because Cse4-R37A protein levels are comparable to wild-type levels, these defects stem from the loss of R37 methylation, demonstrating its importance for kinetochore assembly (Samel et al., 2012).

R37 methylation is minimal in G1, increases during S-phase, and peaks at metaphase (Mishra et al., 2023). In G2/M-enriched cells, the centromeric levels of Cse4 and Cse4-R37A were similar, whereas the methyl-mimic Cse4-R37F showed reduced centromeric occupancy and higher exchange, as determined by FRAP analysis. The R37F mutation caused a ∼50% reduction in kinetochore Ame1, Ctf19 (COMA complex), and Mif2 levels, leading to a 15-fold increase in chromosome loss relative to wild-type and R37A Cse4 (Mishra et al., 2023). These findings indicate that timely R37 methylation is essential for proper Cse4 nucleosome assembly and kinetochore formation during S-phase and for Cse4 stability during mitosis.

The combined deletion of the major arginine methyltransferases Hmt1, Rmt2, and Hsl7 did not affect R37 methylation, indicating that these enzymes are not responsible for this modification. Upa1 emerged as a candidate based on its human ortholog, the SPOUT methyltransferase 1 (CENP-32, Supplementary Table 1), which associates with kinetochores (Ohta et al., 2015) and displays negative genetic interactions with *CENP-P* (*CTF19* in yeast) (Horlbeck et al., 2018). Consistently, *UPA1* deletion in yeast causes growth defects and negative genetic interactions with kinetochore mutants (Costanzo et al., 2010; 2016). Western blot analysis showed that Cse4-R37 methylation was reduced by 40% in *upa1Δ* cells and rescued by plasmid-based expression of *UPA1*. Conversely, Upa1 overexpression significantly increased Cse4-R37 methylation in G2/M and increased chromosome loss, demonstrating a cell cycle-dependent role for Upa1 in Cse4-R37 methylation (Mishra et al., 2023) (Figure 1C). Of note, the temperature sensitivity of *cse4-R37A cbf1Δ* mutant was suppressed by deletion of the E3 ubiquitin ligase *UBR2*, *MUB1* (Ubr2 adapter), and by overexpression of the MIND subunit Dsn1 (Samel et al., 2017), whose levels are regulated by Ubr2–Mub1 (Akiyoshi et al., 2013). Similarly, deleting the E3 ubiquitin ligase *PSH1* rescues the temperature sensitivity of the *cse4-R37A ctf19Δ* mutant (Samel et al., 2017). As Ubr2–Mub1 and Psh1 negatively regulate MIND proteins (Herrero and Thorpe, 2016), these results suggest that stabilizing kinetochore components (by deleting *UBR2*, *MUB1*, and/or *PSH1*) can partially compensate for defective kinetochore assembly caused by loss of Cse4 methylation at R37.

Cse4 purified from *S. cerevisiae* is also monomethylated at K131 and R143 (Tran Nguyen et al., 2023). These residues lie near the DNA entry/exit site of the centromeric nucleosome, where their methylation may influence Cse4–DNA interactions by enhancing DNA unwrapping and destabilizing the nucleosome (Furuyama and Henikoff, 2009; Tran Nguyen et al., 2023). K131 is also ubiquitinated by the E3 ubiquitin ligase Psh1 *in vitro*, although *in vivo* evidence is lacking (Hewawasam et al., 2010). Nonmethylatable K131A and R143A mutants, alone or combined, showed no growth or temperature-sensitive defects, indicating that these modifications are not essential for viability. However, in the *spc25-1* background (Spc25 is a subunit of the Ndc80 complex), the K131A and/or R143A substitutions exacerbated the mutant’s growth defect and increased plasmid loss. The *cse4-R143A spc25-1* double mutant exhibited a G2/M delay at the semi-permissive 30 °C, a phenotype not observed in either single mutant, indicating that a loss of methylation at R143 and/or K131 impairs kinetochore function in a genetically compromised (*spc25-1*) background. Similar effects were observed with charge-altering R143 substitutions (R143E: negative; R143Q: neutral). In contrast, mutations in other subunits of the Ndc80 complex (Ndc80, Nuf2, or Spc24), CBF3, or *cbf1Δ* showed no genetic interaction with *cse4-K131A* or *cse4-R143A*. Together, these data suggest that methylation at K131 and R143 contributes to Cse4–DNA interactions and to coupling the centromeric nucleosome with the outer kinetochore (Tran Nguyen et al., 2023).

A suppressor screen using the *cse4-R143A spc25-1* mutant identified a P253S mutation in the lysine methyltransferase Set2 that partially rescued the mutant’s temperature-sensitive growth defect. Set2, which associates with RNA polymerase II (RNA pol II) to methylate H3K63 and facilitate transcription elongation (Krogan et al., 2003; Xiao et al., 2003), also mono-methylates H3K37 to promote origin licensing and DNA replication (Santos-Rosa et al., 2021). Importantly, deleting *SET2* rescued the growth defect and G2/M arrest of *cse4-R143A spc25-1* cells at semi-permissive temperature. Similar rescue of *cse4-R143A spc25-1 set2Δ*, and *cse4-R143A-K131A spc25-1* mutants suggests that Set2 likely methylates Cse4 at K131 (Tran Nguyen et al., 2023) (Figure 1C).

#### Phosphorylation

ChIP analyses identified phosphorylation of Cse4 at S22, S33, S40, and S105, with the phosphorylated form showing enhanced centromere association compared to its dephosphorylated form (obtained following phosphatase treatment). The phosphorylation of centromeric Cse4 is cell cycle-dependent, increasing in G2/M cells. Given that the Aurora B kinase Ipl1 activates the SAC in response to erroneous kinetochore-microtubule attachments, its role in phosphorylating Cse4 was investigated. The *ipl1-2* mutant exhibited a three-fold reduction in phosphorylated Cse4 levels at centromeres, despite normal intracellular Cse4 levels (Boeckmann et al., 2013). *In vitro* kinase assays confirmed that Ipl1 phosphorylates Cse4 at S22, S40, S105, Y106, S124, and S148 but not at S33 (Figure 1C), suggesting that a different kinase targets S33 *in vivo*. To determine the functional importance of the four *in vivo* phosphorylated Cse4 sites (S22, S33, S40, and S105), phosphomimetic (Cse4-4SD) and phospho-null (Cse4-4SA) variants were created in the *ipl1-2* background. Cse4-4SD partially rescued the temperature sensitivity of *ipl1-2* cells, whereas Cse4-4SA did not. Moreover, Cse4-4SD suppressed growth defects in *ipl1-2* strains carrying phospho-null mutations in Ipl1-targeted kinetochore proteins involved in microtubule attachment, such as Ndc80 and the DASH subunits Dam1 and Spc34. Together, these findings indicate that Ipl1-mediated Cse4 phosphorylation stabilizes kinetochores with impaired microtubule attachments and promotes chromosome bi-orientation when the phosphorylation of other Ipl1 substrates is compromised (Boeckmann et al., 2013).

The four *in vivo* phosphorylated residues of Cse4 are located at its N-terminus, with S33 and S40 positioned within the END domain, which mediates the interactions with Scm3 and Okp1–Ame1. Combining the phosphomimetic *cse4-4SD* allele with *okp1-5* or *ame1-4* mutations exacerbated the temperature sensitivity of the latter mutants, indicating that constitutive phosphorylation of these four residues destabilizes kinetochores when Okp1–Ame1 function is compromised (Boeckmann et al., 2013). This genetic interaction highlights a functional link between Cse4 phosphorylation and COMA activity. Consistently, depleting Ipl1 reduced Cse4 centromere occupancy by 40%, a defect that was partially rescued by the phosphomimetic Cse4-S40D, which restored Ame1 centromeric levels (Popchock et al., 2025). In contrast, Scm3 binding to the phospho-null Cse4-S40A was reduced. Together, these findings suggest that Ipl1-mediated phosphorylation of Cse4 at S40 stabilizes the association of Cse4 and Scm3 with centromeric DNA and the Okp1–Ame1 complex, likely in a temporally regulated manner. Given the physical interaction between Ipl1 and Ndc10, the phosphorylation of Cse4 at S40 likely occurs directly at the centromere (Biggins et al., 1999).

Cdc5, the conserved polo-like kinase that is essential for mitosis and chromosome segregation (Botchkarev and Haber, 2018; Colicino and Hehnly, 2018; Klemm et al., 2021), interacts with Cse4 (Snead et al., 2007), specifically during G2/M. *In vivo*, Cse4 was shown to be phosphorylated in a Cdc5-dependent manner (Mishra et al., 2019). *In vitro*, Cdc5 phosphorylates Cse4 at nine serines (Figure 1C): eight in the N-terminal tail (S9, S10, S14, S16, S17, S33, S40, and S105) and one in the HFD (S154). Inactivating Cdc5 reduced Cse4 centromere occupancy by ∼50% and increased centromeric chromatin accessibility, implicating Cdc5 in Cse4 deposition and nucleosome formation. The non-phosphorylatable Cse4-9SA mutant complemented the *cse4Δ* mutation and localized to centromeres, whereas the phosphomimetic Cse4-9SD did not, suggesting that regulated phosphorylation is critical. Additionally, the *cse4-9SA* mutant showed increased chromosome loss and reduced centromeric levels of Mif2 and cohesin when combined with COMA complex mutations (e.g., *mcm21Δ*). This supports a model where Cdc5-mediated phosphorylation of Cse4 is required for cohesin and kinetochore recruitment (Mishra et al., 2019). Because Ipl1 and Cdc5 target overlapping residues, they likely regulate Cse4 in different contexts, for example, depending on the cell cycle, centromeric nucleosome or kinetochore assembly state, or during sister chromatid pairing and/or bi-orientation (Mishra and Basrai, 2019).

Cdc5-dependent phosphorylation of Cse4 at S33 is dispensable for viability, as neither phospho-null (S33A) nor phosphomimetic (S33E) mutants displayed growth defects, even in combination with *cbf1Δ*, mutations in the CBF3 or COMA complexes, downstream kinetochore components, or a nonmethylatable R37A mutation (Hoffmann et al., 2018). However, both S33 mutants displayed strong synthetic growth defects when combined with a histone H4 allele with mutations at three essential N-terminal lysines (K5, K8, and K12) that are required for nucleosome assembly (Choy et al., 2012), showing impaired growth and increased sensitivity to nocodazole and hydroxyurea. These defects were most pronounced in the *cse4-S33A* background, which showed reduced centromeric levels of Cse4-S33A relative to Cse4-S33E in the H4 triple-lysine mutant (Hoffmann et al., 2018). Synthetic growth defects were also observed between *cse4-S33A* or *cse4-S33E* and mutations in histones H2A and H2B. In particular, Cse4-S33E interacted strongly with acidic patch mutations in H2A that impair H2A–H2B deposition (Hodges et al., 2017). Together, these data suggest that S33 phosphorylation modulates Cse4 deposition or stability at centromeres, potentially by affecting Scm3 recruitment or binding (Hoffmann et al., 2018).

The conserved Cdc7–Dbf4 (DDK) kinase complex associates with centromeric chromatin to promote DNA replication (Natsume et al., 2013; Rossbach et al., 2017) and regulate cohesin loading via phosphorylation of the COMA subunit Ctf19 (Hinshaw et al., 2017). Cdc7 also phosphorylates Cse4 *in vitro* (Mishra et al., 2021b), likely targeting consensus S/T-D/E motifs in the HFD (S148, T149, T166, and S190) (Figure 1C). Because Cdc7 can also phosphorylate S/T residues that are preceded by an S/T that has been primed by another kinase, such as Cdc5, additional candidate sites include S9, S10, and S14 in the N-terminal tail (Mishra et al., 2019). Although Cdc7 and Cse4 interact primarily during S-phase, Cdc7 remains centromere-associated throughout the cell cycle, with a peak in G1 and S-phase. In *cdc7-7* mutants, centromeric Cse4 levels were modestly reduced in S-phase but significantly reduced in G2/M. FRAP analyses revealed increased Cse4 turnover at metaphase centromeres, indicating a role for Cdc7 in Cse4 maintenance. Consistently, alanine substitutions at the four Cdc7 candidate target residues in the Cse4 HFD, or at all seven candidate Cdc7 sites, including the three N-terminal residues, led to an increase in chromosome loss. The phosphomimetic Cse4-4D failed to complement the *cse4Δ* mutant (Mishra et al., 2021b). Because the HFD mediates interactions with centromeric DNA, histone H4, and Scm3 during nucleosome assembly, a loss or constitutive phosphorylation of these residues likely disrupts these interactions, explaining the reduced centromeric retention of Cse4 in the absence of Cdc7 activity.

Overall, the complex phosphorylation landscape of Cse4 likely enables precise spatiotemporal control of centromeric nucleosome assembly, kinetochore formation, and activity throughout the cell cycle (Fuller et al., 2008; Lampson and Cheeseman, 2011). However, the mechanisms underlying kinase specificity and coordination with opposing phosphatases, such as Glc7 and PP2A, remain unclear (Wang and Ng, 2006, p. 2; Klemm et al., 2024). Recent yeast two-hybrid screens have identified interactions between Cse4 and Snf1 kinase, its regulatory partner Snf4, and the Snf1-activating kinase Sak1 (Hong et al., 2003; Sutherland et al., 2003; Mishra et al., 2018). Along with adapter proteins, the Snf1–Snf4 complex functions in stress-responsive signaling, chromatin remodeling, and transcriptional regulation (Conrad et al., 2014; Salminen et al., 2016). In addition, Cse4 interacted with Sps1, a kinase that phosphorylates histone H4 (Krishnamoorthy et al., 2006) and contributes to spindle disassembly and cytokinesis during meiosis II (Paulissen et al., 2020). These observations suggest that kinases Snf1–Snf4, Sak1, Sps1, and additional, as-yet-uncharacterized kinases and phosphatases may play important roles in regulating Cse4 and centromeric chromatin dynamics.

#### Acetylation

Mass spectrometry identified the *in vivo* acetylation of Cse4 at K49 within the END domain (Boeckmann et al., 2013) (Figure 1C). K49 acetylation coincides with R37 methylation during S-phase, suggesting coordinated regulation. Both R37 methylation and K49 acetylation prevent correct binding between Cse4 and COMA’s Okp1–Ame1 dimer. Consistently, the acetyl-mimic Cse4-K49Q failed to rescue the lethality of the *ctf19Δ cse4-R37A* (*cse4-R37A-K49Q*) strain, suggesting that the loss of K49 acetylation partially suppressed the impaired Okp1–Ame1:Cse4-R37A association. Immunoprecipitation experiments identified that Gcn5, as part of the SAGA complex, is the histone acetyltransferase responsible for Cse4K49 acetylation (Anedchenko et al., 2019). The interplay between R37 methylation and K49 acetylation mirrors the methylation–acetylation crosstalk on canonical histones, where combinatorial modifications of the same or adjacent lysines and arginines modulate chromatin accessibility to regulate transcription and DNA repair (Rajan et al., 2020; Zhou et al., 2022). In Cse4, this crosstalk modulates the END-domain interactions with Scm3 and Okp1–Ame1, thereby fine-tuning nucleosome assembly, kinetochore recruitment and stability.

#### SUMOylation

Cse4 is SUMOylated at multiple lysine residues, a modification that regulates its stability and chromatin localization. In *S. cerevisiae*, the E3 SUMO ligases Siz1 and Siz2 redundantly SUMOylate Cse4, as its SUMOylation is abolished in *siz1Δsiz2Δ* cells, resulting in a two-fold increase in Cse4 stability, indicating that SUMOylation promotes its turnover (Ohkuni et al., 2016). A conserved N-terminal motif (64–DKSD-67) identified K65 as a SUMOylation site. The non-SUMOylatable Cse4-K65R mutant showed reduced SUMOylation and ubiquitination, a 50% increase in Cse4 stability, and mislocalization to non-centromeric chromatin. When overexpressed, Cse4-K65R caused a 1.6-fold increase in chromosome loss, demonstrating that K65 SUMOylation targets mislocalized Cse4 for degradation (Ohkuni et al., 2018).

Cse4 also contains a C-terminal SUMOylation motif at K215 and K216. Preventing SUMOylation at these residues (Cse4-K215/216R) impaired Cse4 deposition at centromeres and non-centromeric regions by reducing interactions with the chaperones Scm3 and CAF-1 (Ohkuni et al., 2020). Overexpression of this mutant rescued the synthetic dosage lethality in strains deleted for Cse4-specific E3 ubiquitin ligases, further indicating a role for K215/216 SUMOylation in Cse4 deposition (Ohkuni et al., 2020). Thus, SUMOylation at K65 and K215/216 has opposing functions *in vivo*: K65 promotes Cse4 degradation, whereas K215/216 facilitates Cse4 deposition and, upon overexpression, mislocalization. Importantly, the Cse4-K215/216R mutation suppressed the Cse4-K65R phenotype, suggesting that C-terminal SUMOylation precedes K65 modification and serves a distinct regulatory role. Once Cse4 SUMOylated at K215/216 is ectopically deposited, K65 SUMOylation will trigger its degradation (Ohkuni et al., 2020). Cse4 SUMOylation is conformation-dependent. The CATD mutation Y193A stabilizes a closed conformation of Cse4 by weakening Cse4–H4 interactions, thereby reducing SUMOylation. Overexpression of H4 restored Cse4–H4 binding, promoted an open Cse4 conformation, and rescued SUMOylation of Cse4-Y193A (Ohkuni et al., 2024), underscoring the importance of the Cse4–H4 interaction in regulating the fate of Cse4.

#### A mechanistic model for Cse4 deposition, maintenance, and kinetochore recruitment to centromeres

Building on the above observations, a mechanistic model can be proposed for centromeric Cse4 deposition and maintenance (Figure 2A) (Popchock et al., 2025). Newly synthesized Cse4 adopts a closed conformation due to the internal interaction between END and HFD domains (Malik et al., 2018). Upon binding to histone H4, Cse4 releases the N-terminal tail. When the (Cse4–H4)_2_ tetramer forms, Yta7 unfolds it. Next, one Scm3 molecule binds with high affinity to Cse4’s HFD in each (Cse4–H4) dimer. Both Scm3 molecules next anchor the (Cse4–H4)_2_ tetramer to the CDEII region of the centromere. Subsequently, the Scm3 molecules move from the HFDs of both (Cse4–H4) dimers to the END domain of each Cse4, allowing two (H2A–H2B) dimers to be recruited and complete the nucleosome (Figure 2A). Scm3, via its N-terminus, in concert with Cbf1 and Ndc10/CBF3, then mediates tight DNA wrapping and end-sealing around the nascent nucleosome (Cho and Harrison, 2011; Dechassa et al., 2011; Shivaraju and Gerton, 2011; Shivaraju et al., 2011). Scm3 remains at the centromere (Cho and Harrison, 2011) and is additionally recruited in early anaphase to stabilize the Cse4 nucleosome during chromosome segregation. Once deposited, Cse4 is stabilized by coordinated modulation of END-domain interactions. R37 methylation and S40 phosphorylation strengthen binding to Okp1–Ame1 and Scm3, whereas S33 phosphorylation and K49 acetylation weaken these associations, allowing the fine-tuning of kinetochore assembly. Scm3 and Okp1–Ame1 together anchor the mature Cse4 nucleosome to the inner kinetochore, while additional centromeric factors wrap and seal the DNA, creating the rigid platform needed for microtubule attachment and faithful chromosome segregation.

### Cse4 localizes to centromeres and centromere-like regions

Restricting Cse4 and other eukaryotic H3 variants to centromeric DNA is essential for faithful chromosome segregation (Smith, 2002). However, the mechanisms that regulate H3 variant abundance and their incorporation into centromeric chromatin remain poorly understood in higher eukaryotes. In human cells and *Drosophila*, the overexpression of CENP-A and CID, respectively, causes their ectopic deposition in euchromatin, leading to dicentric chromosomes and genomic instability, which is strongly associated with cancer (Van Hooser et al., 2001; Tomonaga et al., 2003; Heun et al., 2006; Moreno-Moreno et al., 2006; Amato et al., 2009; Dong et al., 2021; Jeffery et al., 2021; Balachandra et al., 2024). By contrast, Cse4 overexpression in *S. cerevisiae* does not induce chromosome loss or lethality, indicating the existence of mechanisms that limit excess Cse4 and prevent its misincorporation outside centromeres (Crotti and Basrai, 2004). The genetic tractability and biochemical accessibility of haploid yeast have therefore made *S. cerevisiae* a powerful model to perform genetic interaction screens to identify conserved factors that regulate Cse4 and restrict its localization to centromeres (Collins et al., 2004).

ChIP sequencing of endogenous Cse4 showed stable enrichment at the 16 centromeres and transient incorporation at highly transcribed loci, characterized by rapid histone turnover, where temporarily deposited Cse4 is quickly replaced by histone H3 (Au et al., 2008; Lefrancois et al., 2009). Upon Cse4 overexpression, its non-centromeric association increases and can recruit kinetochore proteins, including Ndc10, Mif2, and Ndc80 (Lefrancois et al., 2013). Twenty-three such sites, termed centromere-like regions (CLRs), are primarily pericentromeric and contain an AT-rich ∼90 bp sequence that resembles CDEII (Baker and Rogers, 2005). Cse4 and kinetochore occupancy at CLRs is substantially lower than at native centromeres, confirming that centromeres are the primary binding sites for Cse4 and kinetochores, even under conditions of Cse4 overexpression. However, CLRs can partially mimic centromere function: they weakly stabilize acentric plasmids and, adjacent to an inactivated centromere, can support chromosome segregation in a Cse4 overexpression and Ndc10-dependent manner (Lefrancois et al., 2013). CLR activity is independent of cohesin (Lefrancois et al., 2013), which forms the pericentromeric C-loop that isolates the centromeric nucleosome and balances the tensions from spindle microtubules during bi-orientation (Yeh et al., 2008; Stephens et al., 2011; Lawrimore and Bloom, 2019) (Figure 3). The pericentromeric localization of CLRs suggests they may represent evolutionary remnants of regional centromeres (Lefrancois et al., 2013).

#### Cse4 is required for segregating the multicopy 2-µm plasmid

Most wild *Saccharomyces* strains harbor the native 2-micron (2-µm) plasmid (Stevens and Moustacchi, 1971), a multicopy (40–60 copies per cell) selfish DNA element that encodes its own replication and amplification functions without affecting host fitness (Rizvi et al., 2018). Stable inheritance of the plasmid depends on a dedicated partitioning system rather than a canonical centromere. This system is centered on the ∼600 bp STB (stability) locus, which contains five AT-rich tandem repeats bound by the Rep1–Rep2 complex that recruits Cse4 (Hajra et al., 2006). Two STB repeats resemble the ∼120-bp centromeric sequence that accommodates a single Cse4 nucleosome, suggesting that the STB may enclose one or two Cse4 nucleosomes. Whether this number depends on Cse4 availability remains unknown (Huang et al., 2011b).

A chromosomally integrated STB can recruit Cse4 and function as a CLR (Huang et al., 2011a). Notably, Cse4 integrated at STB sites is protected from degradation: upon shut-off of Cse4 overexpression, global Cse4 levels declined, yet Cse4 at STB and centromeres remained stable (Hajra et al., 2006). These observations have led to the hypothesis that yeast centromeres may have evolved from an ancestral STB following integration of a 2-µm plasmid, eventually acquiring the additional features necessary for efficient chromosome segregation (Malik, 2006; Liu et al., 2013).

Unlike centromeres, the STB recruits Cse4 independently of Ndc10/CBF3 and does not assemble a canonical kinetochore. Instead, the STB forms a partitioning complex (Hajra et al., 2006) that incorporates cohesin for plasmid pairing (Mehta et al., 2002; Ghosh et al., 2007; 2010) and microtubule-associated proteins, including Bik1, Bim1, and Cip1/Kip1, to mediate spindle-mediated segregation (Cui et al., 2009; Prajapati et al., 2017). After mitosis, Cse4 and the microtubule-binding factors dissociate from the STB, while Rep1–Rep2 reassociate in early S-phase to recruit Cse4 for the next cell cycle (Hajra et al., 2006). Given their copy number, 2-µm plasmids may act as sinks for excess Cse4, thereby limiting its ectopic chromatin incorporation. Thus, despite their classification as selfish elements, 2-µm plasmids may contribute to genome stability by sequestering surplus Cse4 (Rizvi et al., 2018). The ability of Rep1–Rep2 to bind to chromosomal DNA in a sequence-independent manner suggests a potential role for this complex in ectopic Cse4 deposition. Because yeast Rep1–Rep2 can also associate with mammalian DNA (Liu et al., 2016) (Rep1–Rep2 orthologs remain unidentified), analogous mechanisms regulating excess CENP-A may operate in higher eukaryotes.

### Ubiquitin-mediated proteolysis restricts Cse4 to centromeres

#### Non-ubiquitinated Cse4 mislocalizes beyond centromeres

Endogenous Cse4 has a half-life of ∼30 min (Au et al., 2013). It is stabilized upon proteasome inhibition, indicating that ubiquitin-dependent degradation by the 26S proteasome is central to Cse4 homeostasis (Collins et al., 2004). Both endogenous and overexpressed Cse4 are ubiquitinated, with the N-terminal serving as the primary ubiquitination site. Substitution of all eight N-terminal lysines with arginine markedly reduced ubiquitination, increased Cse4 stability three-fold relative to wild-type, and increased chromosome loss, whereas mutation of the eight C-terminal lysines had a smaller effect (Au et al., 2013). Complete substitution of all 16 lysines (Cse4-16R) abolished ubiquitination. Endogenous Cse4-16R rescued a *cse4Δ* mutant without detectable chromosome missegregation (Au et al., 2008), while its overexpression led to centromeric deposition, mislocalization to non-centromeric chromatin, and a 19-fold increase in chromosome missegregation. This mislocalization was suppressed by overexpression of H3 lacking its N-terminal regulatory element (residues 3–40; H3Δ3–40), which competes for histone H4 binding (Au et al., 2008). Although overexpressed Cse4-16R was more stable than overexpressed wild-type Cse4, it remained subject to turnover, suggesting additional proteasome-independent degradation pathways. Notably, overexpressed Cse4 degradation followed non-linear kinetics, consistent with rapid turnover of the soluble pool and relative protection of chromatin-bound Cse4. Accordingly, overexpression of H3–H4 reduced Cse4-16R mislocalization by displacing it from euchromatin into the degradable soluble pool (Au et al., 2008). Together, these findings establish proteolysis as a key mechanism controlling Cse4 abundance and centromeric confinement, while excess Cse4 incorporation outside centromeres may disrupt transcription and overwhelm cellular degradation capacity.

#### RING E3 ubiquitin ligase Psh1 targets excess Cse4 for proteasomal degradation via CATD binding

To identify proteins involved in confining Cse4 to centromeres, wild-type Cse4 (Hewawasam et al., 2010) and the lysine-free mutant Cse4-16R (Ranjitkar et al., 2010) were affinity-purified from yeast, resulting in the identification of the nonessential, 406-residue protein Psh1 (Pob3/Spt16 histone-associated 1). Reciprocal purifications of Psh1 similarly recovered Cse4, in addition to the four canonical histones, variant H2A.Z (Htz1), and numerous kinetochore components (Hewawasam et al., 2010; Ranjitkar et al., 2010). Psh1 localized to centromeres throughout the cell cycle and bound both chromatin-associated (Hewawasam et al., 2010) and soluble Cse4 (Ranjitkar et al., 2010). Their interaction was maintained in an *ndc10-1* mutant, which is unable to recruit Cse4 and form kinetochores, demonstrating that the Psh1–Cse4 association occurs independently of centromere binding (Ranjitkar et al., 2010).

**FIGURE 4:**
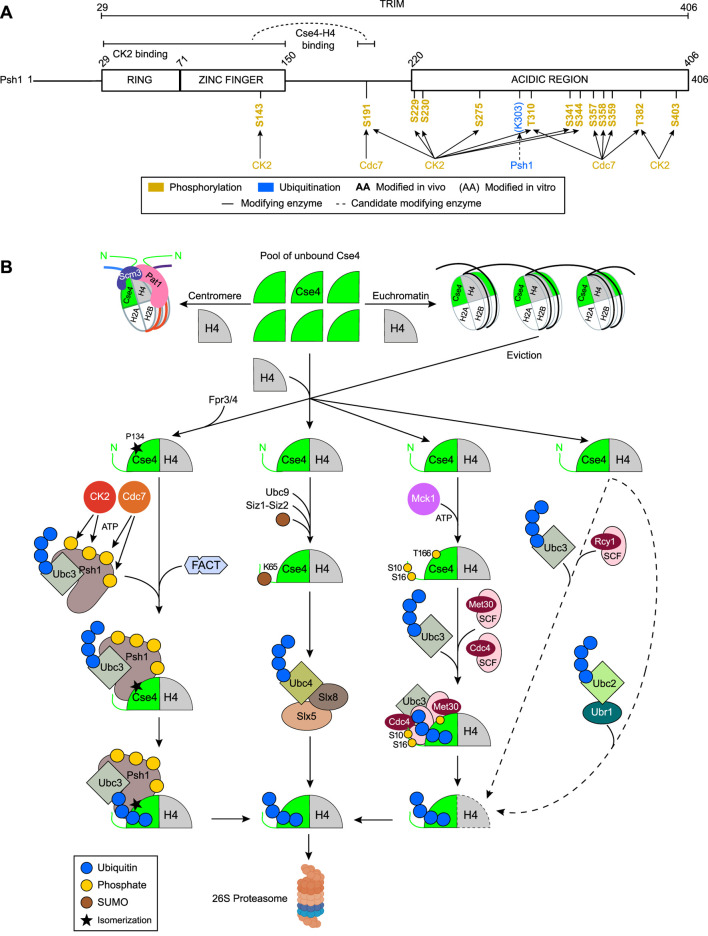
E3 ligase Psh1 and parallel degradation pathways regulating Cse4 levels. **(A)** Domain architecture of Psh1 and its post-translational modifications. Enzymes known to mediate these modifications *in vitro* or *in vivo* are indicated. **(B)** Model for the deposition of endogenous or overexpressed unbound Cse4 at centromeres and its misincorporation at non-centromeric regions. Multiple parallel pathways, defined by specific Cse4 post-translational modifications and the involved enzymes, target Cse4 for ubiquitination and subsequent proteasomal degradation. For simplicity, single Cse4–H4 dimers are depicted.

While its function was unknown, Psh1 qualified as a TRIM (tripartite motif) protein, as it contains an N-terminal RING domain (residues 29–71), a central C4 Zinc finger domain (residues 71–150), and a C-terminal acidic region (residues 220–406) (Figure 4A). The RING domain is characteristic of many E3 ubiquitin ligases that act as interfaces to direct the transfer of ubiquitin from their cognate E2 enzyme onto a substrate, without forming a covalent E3–ubiquitin intermediate. While domain mutagenesis showed that the RING domain is required for the Psh1–Cse4 interaction, both the RING and C4 Zinc domains are required for ubiquitination activity (Hewawasam et al., 2010). *In vitro* ubiquitination assays combined with Western blotting demonstrated that Psh1 polyubiquitinates Cse4 and not the four canonical histones (Hewawasam et al., 2010; Ranjitkar et al., 2010). Mass spectrometry analysis of Cse4 *in vitro* ubiquitinated by Psh1 identified four Psh1-dependent ubiquitination sites: K131 residing in Cse4’s linker region, K155 in loop 1, and K163 and K172 in HFD’s α1 and α2 helices, respectively (Hewawasam et al., 2010) (Figure 1C). K163 was the most ubiquitinated site and is not conserved to H3, suggesting a specific and important role in the *in vivo* regulation of Cse4 stability. Mutating these four residues individually did not reveal any growth or ploidy defects (Camahort et al., 2009). Consistently, Cse4 stability was increased in *psh1Δ* cells (Hewawasam et al., 2010; Ranjitkar et al., 2010), which was similarly observed for the Cse4 mutant protein in which the four lysines were substituted with alanine, confirming that Psh1 activity underlies Cse4 degradation.

Inducibly overexpressing *CSE4* from a *PGAL* promoter on a 2-µm plasmid caused cell death in *psh1Δ* cells, indicating that Psh1 activity is essential for clearing Cse4 when its levels are excessive (Hewawasam et al., 2010). Under these conditions, Cse4 accumulated in both the soluble and chromatin-associated fractions (Ranjitkar et al., 2010), an anomaly that activated the SAC and delayed cell cycle progression. This phenotype suggested defects in kinetochore function and chromatin perturbations at centromeres and regions beyond (Ranjitkar et al., 2010). Notably, *in vitro* ubiquitination assays showed that Scm3 protected Cse4 from Psh1-dependent ubiquitination and subsequent proteasomal degradation (Hewawasam et al., 2010) (Figure 2A). In cells lacking Scm3, Cse4 in both soluble and chromatin-associated pools may be more accessible to Psh1. Overexpression of histone H3 did not reduce the viability of *psh1Δ* cells. Furthermore, Psh1 did not interact with overexpressed H3 (Ranjitkar et al., 2010), indicating that Psh1 discriminates between both histones. Co-immunoprecipitation experiments showed that full-length Cse4, as well as its HFD, but not its N-terminus, interacts with Psh1 (Figure 1C).

Deleting Cse4’s CATD region within its HFD (residues 166–201) (Figure 1C) abolished Psh1 binding and Cse4 degradation, while inserting the CATD sequence in H3 enabled Psh1 binding (Ranjitkar et al., 2010). More specifically, CATD residues T171 and W178 (loop L1) proved sufficient for Psh1 binding, though A189 and S190 in the α2 helix were of primary importance, with other α2 residues further strengthening the CATD–Psh1 interaction (Zhou et al., 2021). Psh1 binds to the reconstituted (Cse4–H4)_2_ tetramer and the (Cse4–H4)_2_(H2A–H2B)_2_ octamer but not to the assembled Cse4 nucleosome. This further suggested that Psh1 requires access to Cse4’s L1 loop and α2 helix, which are occluded by the DNA in the CATD domain (Deyter and Biggins, 2014; Zhou et al., 2021). In cells, kinetochore proteins bound to the nucleosome may further restrict Psh1 access, implying that Psh1 primarily targets soluble or evicted Cse4, or Cse4 that is transiently exposed from the nucleosome.

Mass spectrometry identified K131 (linker region), K155 (loop 1), and K163 and K172 (HFD’s α1 and α2 helices, respectively) as Cse4 lysines targeted by Psh1 (Hewawasam et al., 2010) (Figure 1C). K163 is the most ubiquitinated site and is not conserved in H3, suggesting a specific and important role in regulating Cse4 stability *in vivo*. Mutating these four residues individually did not reveal growth or ploidy defects (Camahort et al., 2009). The mutant Cse4 exhibited increased stability, comparable to wild-type Cse4 expressed in *psh1Δ* cells (Hewawasam et al., 2010; Ranjitkar et al., 2010), confirming that Psh1 activity underlies Cse4 degradation. Furthermore, Psh1-mediated degradation is essential to counteract excessive Cse4 levels, as evidenced by cell death when Cse4 is overexpressed in *psh1Δ* cells (Hewawasam et al., 2010). However, cycloheximide translation-shut-off experiments showed that Cse4 was still degraded in the absence of Psh1, suggesting that additional, Psh1-independent pathways contribute to Cse4 turnover (Collins et al., 2004) (Figure 4B).

#### Cse4 mislocalization in cells lacking Psh1 activity

To assess the impact of Psh1 loss on Cse4 localization, Cse4-binding sites were mapped by ChIP sequencing in wild-type and *psh1Δ* cells (Hildebrand and Biggins, 2016). In wild-type yeast, endogenous Cse4 was confined to centromeres, whereas *psh1Δ* cells showed additional enrichment in pericentromeric regions. Upon Cse4 overexpression, a large fraction of the protein localized to AT-rich intergenic regions, including promoters (46% in *PSH1* and 90% in *psh1Δ* strains), with minimal occupancy within coding sequences (Lefrancois et al., 2013). In *psh1Δ* cells, this intergenic preference was further skewed toward promoters. Cse4 overexpression in *psh1Δ* cells resulted in differential expression of 294 genes, with 184 upregulated and 110 downregulated compared to wild-type yeast endogenously expressing or overexpressing Cse4 (Hildebrand and Biggins, 2016). Downregulated genes showed Cse4 enrichment at promoter sites bound by the Mediator subunit Med9, suggesting impaired mediator function and reduced RNA pol II transcription. In contrast, upregulated genes exhibited Cse4 enrichment in nucleosome-depleted promoter regions but not at the +1 nucleosome. Notably, most promoters bound by Cse4 displayed no transcriptional change, indicating that gene expression effects depend on the level, position, and stability of the ectopically incorporated Cse4 (Hildebrand and Biggins, 2016).

#### Cse4 P134 isomerization by Fpr3 and Fpr4 regulates Psh1–Cse4 binding

The yeast peptidyl-prolyl cis–trans isomerase Fpr3 was identified as a kinetochore-associated protein through co-purification with the kinetochore component Dsn1 (Akiyoshi et al., 2010) and centromeric DNA (Ohkuni and Kitagawa, 2011). Fpr3 interacts with the histone H3 N-terminal tail, like its paralog Fpr4, which isomerizes histone H3 at proline 38 (H3P38) to block H3K64 methylation and regulate gene expression (Nelson et al., 2006). To assess whether Fpr3 and Fpr4 regulate Cse4, endogenous Cse4 levels were measured in *fpr3Δ* and *fpr4Δ* single mutants and in the double mutant. Cse4 concentrations increased ∼50% in the single mutants and two-fold in the double mutant relative to wild-type (Ohkuni et al., 2014). Overexpressed Cse4 was more stable in the *fpr3Δ* mutant than in wild-type or *psh1Δ* cells. Cse4 stability was the highest in a *psh1Δ fpr3Δ* double mutant, indicating that Fpr3 activity promotes Cse4 degradation by Psh1 (Figure 4B). Mutation analysis of the five prolines in Cse4 identified P134 as critical for stability; its substitution with valine produced the strongest stabilizing effect. P134 lies within the α1 helix of the histone-fold domain, proximal to four lysines (K131, K155, K163, and K172) that are ubiquitinated by Psh1 (Figure 1C). Consistent with a role in proteolysis, Psh1–Cse4 binding was reduced by ∼40% in *fpr3Δ*, *fpr4Δ*, *fpr3Δ fpr4Δ*, and *cse4-P134V* mutants, indicating that P134 isomerization enhances the Psh1–Cse4 interaction (Ohkuni et al., 2014). Accordingly, centromeric Cse4 levels were elevated in *fpr3Δ* and *fpr3Δ fpr4Δ* strains, with the stronger effect in the double mutant suggesting partial redundancy between Fpr3 and Fpr4. The specific proline conformation (*cis* or *trans*) that promotes Cse4 degradation remains unresolved.

#### Phosphorylation modulates Psh1 activity to promote Cse4 degradation

To investigate the post-translational regulation of Psh1, mass spectrometry was performed on Psh1 purified from whole-cell extracts and on kinetochore-associated Psh1 co-purified with Dsn1. Ten phosphorylation sites were identified (eight serines and two threonines). T382 and S403 were specific to kinetochore-associated Psh1, whereas S229, S230, and S275 were unique to soluble Psh1. The remaining sites (S143, S191, T310, S341, and S344) were shared by both (Hewawasam et al., 2014). These findings suggested differential regulation of soluble and kinetochore-bound Psh1. Most phosphorylation sites lie in the C-terminal half of Psh1 and were largely excluded, except for S143, from the RING and C4 Zinc finger domains that mediate Cse4 ubiquitination and control Cse4 levels *in vivo* (Hewawasam et al., 2010; Ranjitkar et al., 2010; Zhou et al., 2021) (Figure 4A).

In cells endogenously expressing a non-phosphorylatable Psh1-S8A/T2A mutant, overexpressed Cse4 displayed reduced ubiquitination, a five-fold increase in stability, and a slow-growth phenotype. In contrast, a phosphomimetic mutant (Psh1-S6D, S143D, S191D, S229D, S230D, S275D, and S344D) decreased Cse4 stability two-fold. Because both mutants retained Cse4 binding, the altered stability likely reflected changes in ubiquitination efficiency. Notably, phosphorylation of T310, S341, T382, and S403 is critical for Psh1 activity, as the Psh1-S6A mutant could not phenocopy the 10-alanine mutant. Among these, T382 and S403 were phosphorylated only in kinetochore (Dsn1)-associated Psh1. The restriction of T382 and S403 phosphorylation to kinetochore-associated Psh1 suggests a mechanism for spatially regulated Cse4 degradation, potentially enabling selective ubiquitination of Cse4 that is, for example, displaced from the centromeric nucleosome (Hewawasam et al., 2014).

Casein kinase CK2 is a candidate kinase for Psh1, as all four CK2 subunits (catalytic subunits Cka1 and Cka2 and regulatory subunits Ckb1 and Ckb2) co-purified with Psh1 (Hewawasam et al., 2014). Direct phosphorylation was confirmed by *in vitro* kinase assays, while *in vivo* deletion of *CKA2* produced the strongest reduction in Psh1 phosphorylation, as assessed by Phos-tag immunoblotting. Co-immunoprecipitation experiments showed that Psh1 preferentially interacts with the regulatory subunit Ckb1 via its RING domain (Hewawasam et al., 2010), suggesting that Ckb1 recruits CK2 to enable Cka2-dependent phosphorylation of Psh1 (Hewawasam et al., 2014). Functionally, *cka2Δ* cells overexpressing Cse4 exhibited a slow-growth phenotype, indistinguishable from *psh1Δ* cells, with no additive effect in the double mutant, indicating that CK2 and Psh1 act in the same pathway. Overexpressed Cse4 accumulated to approximately threefold and two-fold higher levels in *cka2Δ* cells than in wild-type and *psh1Δ* strains, respectively. Endogenous Cse4 was 1.5-fold more stable in *cka2Δ* cells, without showing a detectable change in ubiquitination, whereas ubiquitination of overexpressed Cse4 was strongly reduced relative to the wild-type and *psh1Δ* strains. These findings suggest that CK2 regulates Cse4 stability through both Psh1-dependent and Psh1-independent mechanisms. The latter may occur via regulating Fpr3 (Wilson et al., 1997) or activation of the Psh1’s partner, the E2 ubiquitin-conjugating enzyme Ubc3, which is activated by CK2 (Coccetti et al., 2008).

Previous *in vitro* experiments showing that Psh1 undergoes auto-ubiquitination at K303 (Hewawasam et al., 2010) (Figure 4A) suggested that CK2 might promote Psh1 self-degradation. However, analysis of the Psh1 phosphomutants revealed the opposite effect. CK2-mediated phosphorylation increased Psh1 stability: the phosphomimetic Psh1-S6D mutant was stable, whereas a non-phosphorylatable 10-alanine mutant exhibited a two-fold reduction in stability. Thus, CK2 phosphorylation stabilizes Psh1 and increases its activity toward Cse4 (Hewawasam et al., 2014) (Figure 4B).

The stabilization of overexpressed Cse4 in *cka2Δ* cells resulted in its mislocalization to non-centromeric sites like the rDNA locus and *PHO5* promoter, albeit less extensively than in the *psh1Δ* mutant. Notably, the *cka2Δ psh1Δ* double mutant exhibited more severe mislocalization of Cse4 than in either single mutant. Because the Psh1–Cse4 interaction is preserved in the *cka2Δ* strain, unphosphorylated Psh1 may partially restrain ectopic deposition by limiting the pool of free Cse4. In the *cka2Δ psh1Δ* mutant, loss of this restraint likely increases the availability of stabilized Cse4, leading to increased misincorporation (Hewawasam et al., 2014).

Stabilization and reduced ubiquitination of overexpressed Cse4 were also observed in mutants of the essential kinase Cdc7 (*cdc7-7)* and its activator Dbf4 (Eisenstatt et al., 2020). Cse4 overexpression in *cdc7-7* caused mislocalization across the chromatin, implicating Cdc7 in the clearance of excess Cse4. The growth defects and Cse4 mislocalization in the *cdc7-7* mutant mirrored those seen in the *psh1Δ* strain overexpressing Cse4 (Hewawasam et al., 2010; Ranjitkar et al., 2010). The absence of additive effects with respect to cell viability or Cse4 stability in *cdc7-7 psh1Δ* double mutants indicated that Cdc7 and Psh1 act in the same pathway. This suggests that Cdc7 may activate Psh1 (Eisenstatt et al., 2020); indeed, Psh1 contains 23 predicted Cdc7 phosphorylation sites, six of which (S191, T310, S357, S358, S359, and T382) have been experimentally validated (Engel et al., 2022) (Figure 4A). Further work should clarify how Cdc7, potentially in coordination with CK2, activates Psh1 to promote the turnover of excess Cse4 (Figure 4B).

#### Pat1 and Scm3 protect centromeric Cse4 from Psh1-mediated ubiquitination

The protein associated with topoisomerase II 1 (Pat1) is an mRNA decapping enzyme and P-body component (Nissan et al., 2010) that also localizes to centromeres in an Ndc10-dependent manner (Mishra et al., 2013). *pat1Δ* cells exhibited increased chromosome loss and segregation defects, which were phenocopied by deletion of the Pat1 decapping domain (residues 245–422), indicating a centromeric function independent of mRNA turnover (Mishra et al., 2013). A conserved 29-amino acid region within this domain (residues 279–308) is also present in the human ortholog PAT1L (Supplementary Table 1). At centromeres, *pat1Δ* cells show loss of chromatin integrity and a ∼50% reduction in Cse4 levels without changes in Cse4 mRNA concentrations, indicating defective Cse4 stability or deposition/maintenance (Haase et al., 2013; Mishra et al., 2013). Unlike wild-type cells, in which Cse4 ubiquitination peaks at G2/M, *pat1Δ* cells displayed increased ubiquitination throughout the cell cycle (Mishra et al., 2015). Consistently, centromeric Psh1 was enriched at G2/M in the *pat1Δ* mutant, and Pat1 physically interacted with Psh1, limiting its access to Cse4 (Mishra et al., 2015). These findings indicate that Pat1 protects centromeric Cse4 by competitively inhibiting Psh1-mediated ubiquitination.

At centromeres, Pat1 also physically interacts with the Cse4 chaperone and protector Scm3 (Mishra et al., 2015), suggesting that Pat1 and Scm3 cooperate to shield Cse4 from Psh1-mediated ubiquitination. Consistent with this model, Psh1 overexpression in wild-type cells accelerated Cse4 degradation, reduced its centromeric localization, and increased centromeric chromatin accessibility, phenocopying *pat1Δ* (Mishra et al., 2015), likely by outcompeting Pat1 and Scm3. Conversely, Scm3 depletion destabilized Cse4 and sensitized Psh1-deficient cells. *In vitro*, the Psh1-mediated ubiquitination of Cse4 was inhibited by Scm3 when present in a 10:1 M ratio relative to Psh1 (Zhou et al., 2021). Psh1 promoted the ubiquitination of wild-type Cse4, but not of a chimeric protein where the CATD domain of Cse4 was replaced with that of histone H3 (Zhou et al., 2021). This specificity was explained by the binding of its competitor, Scm3, to the essential MMAS motif (M181/M184/A189/S190) within the Cse4 CATD (Zhou et al., 2011) (Figure 1C). However, despite Scm3’s high-affinity binding to Cse4–H4, Psh1 can disrupt the Scm3:Cse4–H4 complex via its RING domain (residues 29–71), which engages a broader interface on Cse4–H4.

#### RING-type E3 ubiquitin ligases SCF–Rcy1, Slx5–Slx8, and Ubr1 collaborate with Psh1 to promote Cse4 degradation

Although Psh1 and its E2 partner Ubc3 promote Cse4 degradation in yeast, Cse4 levels still declined in *psh1Δ* cells (Hewawasam et al., 2010; 2014; Ranjitkar et al., 2010; Au et al., 2013), indicating the existence of additional degradation mechanisms (Figure 4B). While the proteasome, autophagy, and lysosomal (vacuolar) pathways constitute the major proteolytic systems in eukaryotes (Athane et al., 2015), deletion of key components of autophagy or vacuolar degradation does not affect Cse4 turnover (Singh et al., 2009; Cheng et al., 2016), identifying the ubiquitin–proteasome system as the primary pathway for Cse4 clearance.

A synthetic dosage lethality screen of 96 ubiquitin pathway genes identified *rcy1Δ* as a mutation that compromises growth upon Cse4 overexpression. Rcy1 is an F-box protein that confers substrate specificity to the SCF (Skp1–Cullin-1/Cdc53–F-box) E3 ligase complex. Endogenous Rcy1 and Cse4 interacted both *in vivo* and *in vitro*, and deleting *RCY1* reduced Cse4 ubiquitination (Cheng et al., 2016). Cse4 degradation was more strongly impaired in *psh1Δ rcy1Δ* cells than in either single mutant, indicating that Psh1 and SCF–Rcy1 function in parallel pathways, although residual turnover suggested additional mechanisms (Cheng et al., 2016) (Figure 4B).

Screening nonessential E2 enzymes identified Ubc4 as another contributor to Cse4 turnover, as deleting *UBC4* impaired Cse4 degradation (Cheng et al., 2017). The Ubc4-associated E3 ligases Slx5 and Slx8, which form a heterodimer, were found to bind Cse4 *in vivo* (Ohkuni et al., 2016; Cheng et al., 2017). Slx5–Slx8 ubiquitinates substrates that have been SUMOylated by the Ubc9 (E2)–Siz1/2 (E3) pathway. In the *slx5Δ* and *siz1Δsiz2Δ* mutants, Cse4 stability increased two-fold, indicating that Siz1/2-dependent SUMOylation promotes Cse4 degradation via the Slx5–Slx8 complex (Ohkuni et al., 2016) (Figure 4B). Lysine 65 (K65) in the Cse4 N-terminal tail becomes exposed upon histone H4 binding and serves as a SUMOylation site (Ohkuni et al., 2018) (Figure 2A). The Cse4-K65R mutant exhibited markedly reduced SUMOylation and ubiquitination *in vivo*, a 50% reduction in Slx5 binding, and a 50% increase in stability, comparable to wild-type Cse4 in *slx5Δ* cells. Cse4-K65R was also ∼35% more stable than wild-type Cse4 in a *psh1Δ* background. These findings indicated that K65 SUMOylation promotes Slx5–Slx8-mediated ubiquitination and degradation of Cse4, defining a pathway that functions in parallel to Psh1 (Ohkuni et al., 2018) (Figure 4B).

Proteomic analyses identified the E3 ubiquitin ligase Ubr1 as a Cse4 interactor along with Psh1 (Ranjitkar et al., 2010). In addition, *ubr1Δ* cells exhibit reduced ubiquitination and degradation of both endogenous and overexpressed Cse4 (Cheng et al., 2017). Growth assays revealed a hierarchy among the Cse4-directed E3 ligases: *psh1Δ* and *rcy1Δ* mutants displayed the most severe defects upon Cse4 overexpression, whereas *ubr1Δ* and *slx5Δ* mutants were less affected. Combined deletions of *PSH1*, *RCY1*, *SLX5*, and *UBR1* resulted in additive Cse4 stabilization and mislocalization, indicating that these ligases function independently (Figure 4B). Nonetheless, residual Cse4 turnover persisted in the quadruple mutant, suggesting additional, unidentified degradation pathways that may act redundantly or be engaged under stress conditions such as Cse4 overexpression (Ohkuni et al., 2016; Cheng et al., 2017).

#### The SCF–Met30 and SCF–Cdc4 RING E3 ubiquitin ligase complexes jointly regulate Cse4 degradation

Using the synthetic genetic array (SGA, comprising 4,293 nonessential genes) and a collection of temperature-sensitive (ts) mutants, a Cse4 dosage lethality screen identified 140 negative genetic interactions, defined as cases when combining two mutations produces a more severe fitness defect than either single mutation. Among these were the essential genes encoding F-box proteins Cdc4 and Met30 (Au et al., 2020). SCF–Met30 and SCF–Cdc4 are E3 ubiquitin ligase complexes that associate with the RING protein Rbx1 and the E2 enzyme Ubc3 to ubiquitinate specific substrates (Petroski and Deshaies, 2005). Cse4 overexpression was lethal in the *met30-ts* and *cdc4-ts* strains but not when an N-terminally truncated Cse4 (Cse4Δ129) was overexpressed, implicating the Cse4 N-terminus as the SCF recognition and target region. Consistent with this model, overexpressed full-length Cse4 was more stable and exhibited reduced ubiquitination in both ts mutants compared with wild-type yeast. The mutations in Met30 or Cdc4 also affected endogenous Cse4; ChIP and imaging analyses revealed its mislocalization with the kinetochore protein Mif2, as well as increased centromeric chromatin accessibility, leading to chromosome segregation defects (Au et al., 2020). Met30 and Cdc4 co-immunoprecipitated with overexpressed Cse4, further supporting Cse4 as a substrate of both SCF complexes. Endogenous Cse4 was similarly stabilized in *met30-ts* and *cdc4-ts* mutants, with no additional stabilization in the double mutant, indicating that SCF–Met30 and SCF–Cdc4 act in the same pathway (Figure 4B). Cse4 stability was further increased in a *psh1Δ met30-ts* double mutant, demonstrating that SCF–Met30/Cdc4 functions in parallel to Psh1-mediated Cse4 turnover (Au et al., 2020).

The C-terminal WD40 domain of Met30 is required for its interaction with Cdc4. This finding, immunoprecipitation, and reconstitution experiments support a model in which SCF–Met30 first binds Cse4 and subsequently recruits SCF–Cdc4 (or Cdc4 alone) to promote Ubc3-mediated Cse4 ubiquitination (Au et al., 2020) (Figure 4B). Substrate recognition by SCF F-box proteins requires prior substrate phosphorylation. In the case of Cdc4, this priming is mediated by the kinase Mck1, the yeast ortholog of GSK-3β (Supplementary Table 1) (Perkins et al., 2001; Delgoshaie et al., 2014). Consistent with this requirement, deletion of *MCK1* approximately doubled Cse4 stability, reduced its ubiquitination by ∼50%, and increased its mislocalization to non-centromeric chromatin by 2.5-fold (Zhang et al., 2024). Co-immunoprecipitation experiments demonstrated an *in vivo* interaction between Mck1 and Cse4, suggesting that Cse4 is a direct Mck1 substrate. This hypothesis was supported by Cse4 containing an Mck1 consensus phosphorylation motif with candidate residues S10, S16, and T166 (Ikui et al., 2012) (Figure 1C). Mutation of these residues to alanine stabilized overexpressed Cse4-3A in wild-type cells to levels comparable to those in *mck1Δ* strains. It resulted in reduced ubiquitination, increased mislocalization, and increased chromosome loss, indicating that phosphorylation at these sites is required for efficient Cse4 degradation. Consistently, the interaction between Cdc4 and Cse4 was reduced in both the *mck1Δ* and Cse4-3A mutants, confirming that Mck1-dependent phosphorylation facilitates Cdc4–Cse4 binding. However, the increased growth defect caused by Cse4-3A overexpression in the *mck1Δ* mutant, compared to wild-type cells, suggested that Mck1 also contributes to Cse4 turnover through phosphorylation-independent mechanisms. Notably, S10 and S16 are also phosphorylated by Cdc5 *in vitro* (Mishra et al., 2019), whereas S10 conforms to a Cdc7 consensus motif (Mishra et al., 2021b), indicating that Cse4 degradation is regulated by a complex, cell cycle-coordinated phosphorylation network.

#### Doa1 promotes the N-terminus-dependent degradation of Cse4

*DOA1* (degradation of alpha2 1) encodes a nonessential protein that maintains ubiquitin homeostasis by recycling ubiquitin following proteasomal degradation (Johnson et al., 1995; Zhao et al., 2009). A Cse4 dosage lethality screen identified Doa1 as a contributor to Cse4 turnover: *doa1Δ* cells exhibited reduced free ubiquitin levels, decreased Cse4 polyubiquitination, and increased Cse4 stabilization, phenotypes that were rescued by ubiquitin (*UBI4*) overexpression (Au et al., 2013). Deleting *DOA1* increased the half-life of both overexpressed and endogenous Cse4 relative to wild-type cells (Au et al., 2013). Doa1-dependent regulation requires the Cse4 N-terminus, as fusion of this region to a non-ubiquitinatable Cse4 histone-fold mutant (9K-R) or to the histone-fold domain of histone H3 resulted in strongly reduced ubiquitination and increased stability of Cse4 in the *doa1Δ* mutant compared with *psh1Δ* cells. These findings demonstrate that Psh1 is not the sole regulator of Cse4 proteolysis and that Doa1 is required, together with Psh1, to promote N-terminus-dependent Cse4 degradation (Au et al., 2013).

#### Histone H4 facilitates the Psh1–Cse4 interaction and its proteolysis when mislocalized

A Cse4 dosage lethality screen of a histone H4 alanine-scanning mutant collection was performed to assess whether H4 contributes to Cse4 regulation, given their *in vivo* dimerization (Deyter et al., 2017). The H4-R36A mutant was lethal upon Cse4 overexpression, indicating defective Cse4 turnover. Substitution of R36 with lysine, which preserves positive charge while eliminating arginine-specific methylation, rescued the growth defect. In contrast, substitution with glutamic acid reproduced the dosage lethality, demonstrating that a positive charge at residue 36 is critical (Deyter et al., 2017). In H4-R36A cells, kinetochore structure and function remained intact, yet overexpressed Cse4 accumulated at both centromeric and euchromatic loci. This accumulation correlated with a two-fold reduction in the Cse4–Psh1 interaction, leading to decreased ubiquitination and stabilization of Cse4. The H4-R36A mutation was proposed to alter the structure of the (Cse4–H4)_2_ tetramer or nucleosome, impairing Psh1 access to the Cse4 CATD; notably, H4-R36 contacts DNA near the nucleosome entry/exit site adjacent to the CATD (Tachiwana et al., 2011). The weakened interaction between mutant (Cse4–H4-R36A)_2_ tetramers and Psh1 likely induced the delocalization of Psh1 to 3′-UTRs, as shown by ChIP. Reduced Cse4 ubiquitination in the H4-R36A mutant was accompanied by increased SUMOylation (Deyter et al., 2017), a phenotype shared with *psh1Δ* cells (Ranjitkar et al., 2010; Ohkuni et al., 2016). Consequently, misincorporated Cse4 at the promoter and coding regions during transcription escaped recognition, eviction, ubiquitination, and degradation. The combined accumulation of stable Cse4 and mislocalized Cse4 and Psh1 likely accounted for the observed lethality following Cse4 overexpression in H4-R36A cells (Deyter et al., 2017).

### Histone H3 and H4 levels determine Cse4 localization

#### Low histone H4 levels prevent Cse4 mislocalization

**FIGURE 5:**
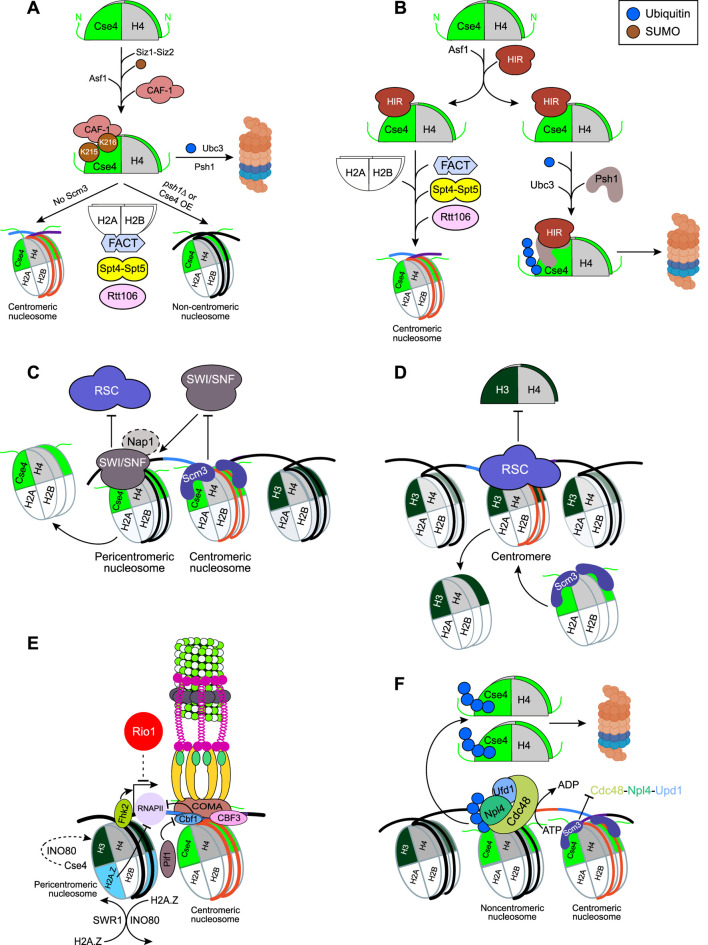
Pathways regulating Cse4 deposition and eviction. Schematics depict the roles of key complexes in controlling Cse4 localization. **(A)** Dual role of the histone chaperone CAF-1 in promoting Cse4 inclusion at centromeres and its mislocalization to non-centromeric regions. The incorporation of H2A–H2B dimers by FACT, Spt4–Spt5, and Rtt106 is also shown. **(B)** Promotion of centromeric Cse4 deposition by the histone chaperones Asf1 and the HIR complex. **(C)** Eviction of Cse4 from non-centromeric nucleosomes by the SWI/SNF chromatin remodeler complex. **(D)** Eviction of misincorporated histone H3 from centromeres by the RSC chromatin remodeler complex. **(E)** Downregulation of centromeric transcription by transcription factors, histone variant H2A.Z, and the assembled kinetochore. The roles of SWR1 and INO80 complexes in depositing and evicting H2A.Z at pericentromeric nucleosomes are also shown. **(F)** Eviction of polyubiquitinated, mislocalized Cse4 by the Cdc48–Npl4–Ufd1 complex.

Deletions of the histone H4 genes *HHF1* and *HHF2* strongly suppressed the slow-growth phenotype of Cse4-overexpressing *psh1Δ* cells and similarly rescued the growth defects of Cse4-overexpressing strains mutated in other degradation pathways (e.g., *cdc4-1, cdc7-4*, *hir2Δ*, *doa1Δ*, and *slx5Δ*) (Eisenstatt et al., 2021). These results suggested that reduced H4 levels promote Cse4 turnover. Although H4 depletion did not affect Cse4 ubiquitination in *psh1Δ* cells, it markedly reduced Cse4 SUMOylation in *hhf1Δ psh1Δ* and *hhf2Δ psh1Δ* mutants, despite normal protein levels of SUMO pathway components. This decrease in SUMOylation specifically affected residues K215 and K216 within the histone-fold domain. SUMOylation at both sites facilitates binding to chaperones such as Scm3 and CAF-1, hence promoting centromeric Cse4 deposition and preventing its mislocalization (Figure 5A) (Hewawasam et al., 2018; Ohkuni et al., 2020). In contrast, SUMOylation at K65 promotes the degradation of mislocalized Cse4 (Figure 4B) (Ohkuni et al., 2018). Disrupting the Cse4–H4 interaction, either by H4 depletion or by mutations disrupting their dimerization, reduced Cse4 SUMOylation and rescued the slow-growth phenotype of Cse4-overexpressing *psh1Δ* strains. Limiting H4 dosage keeps overexpressed Cse4 in a closed conformation that is less accessible to ubiquitination and SUMOylation (Ohkuni et al., 2024), thereby reducing protective chaperone binding, accelerating Cse4 turnover even in the absence of Psh1, and preventing mislocalization while restoring cell viability (Eisenstatt et al., 2021).

#### Low histone H3 levels and a cancer-associated H3 mutation promote Cse4 mislocalization

Histone H4 is the obligate dimerization partner for both histone H3 and Cse4. Therefore, reducing H3 levels increases the pool of free H4 available to bind Cse4, promoting its incorporation beyond centromeres (Au et al., 2008; Eisenstatt et al., 2021; Ohkuni et al., 2024). In *S. cerevisiae*, *HHT1* and *HHT2* encode histone H3. Deleting either gene increased the relative availability of H4 for Cse4 binding approximately fourfold. As a result, Cse4 overexpression in *hht1Δ* or *hht2Δ* strains caused more severe growth defects than in wild-type cells, due to increased Cse4–H4 binding, the consequent adoption of an open Cse4 conformation, increased global Cse4 SUMOylation, and increased Cse4 stability, followed by mislocalization (Ohkuni et al., 2024; 2025). These observations predicted that H3 mutants defective in H4 binding should phenocopy reduced H3 dosage. To test this, three H3 mutations residing at the H3–H4 dimer interface (Y99A, E97A, and E97K) were examined (White et al., 2001; Singh et al., 2009). Notably, E97 is evolutionarily conserved and ranks among the five most frequently mutated H3 HFD mutations in cancer (Nacev et al., 2019). Overexpression of these H3 mutants in an *hht1Δ* background did not affect total Cse4 or H4 levels. The H3–Y99A mutant led to increased Cse4–H4 binding, promoting an open Cse4 conformation and increasing Cse4 SUMOylation and synthetic dosage lethality in *psh1Δ* cells. The H3-E97A and H3-E97K mutants showed reduced binding to histone H4 and decreased stability *in vivo*. Their overexpression similarly enhanced Cse4–H4 association, increased SUMOylation, and induced synthetic dosage lethality in *psh1Δ* strains overexpressing Cse4 (Ohkuni et al., 2025).

### Roles of chromatin remodelers and histone chaperones in Cse4 deposition and extraction

Chromatin remodelers are large ATP-dependent complexes that regulate DNA accessibility by restructuring nucleosomes, including repositioning, eviction of histone dimers or tetramers, and exchanging canonical histones with variants. These activities are essential for transcription, replication, repair, and recombination. Remodelers frequently act in concert with histone chaperones, which are ATP-independent proteins or small complexes that bind histones directly to mediate nucleosome assembly, disassembly, and histone exchange, while preventing histone aggregation and degradation. Many chaperones exhibit substrate specificity, acting on H3–H4 (e.g., CAF-1, HIRA, and Asf1) or H2A–H2B (e.g., FACT, Nap1, and Chz1). In contrast, others specialize in histone variant dynamics, including SWR1 (H2A.Z exchange), HIRA (H3.3 deposition), Scm3 (Cse4–H4 deposition at centromeres), and Yta7 (unfolding of Cse4–H4 tetramers) (Avvakumov et al., 2011; Martire and Banaszynski, 2020; Torres-Arciga et al., 2022).

#### CAF-1 histone chaperone complex

The conserved heterotrimeric chromatin assembly factor-1 (CAF-1) complex, composed of the yeast subunits Cac1–3, deposits them onto newly replicated DNA (Volk and Crispino, 2015). CAF-1 receives nascent H3–H4 dimers from Asf1, promotes H3–H4 tetramer formation, and deposits them onto newly replicated DNA. Although classically considered as replication-coupled, CAF-1 is also recruited to actively transcribed regions in yeast (Chen B. et al., 2023). CAF-1 activity is nonessential due to functional redundancy with the chaperone Rtt106, as evidenced by the inviability and severe nucleosome assembly defects of the *cac1Δ rtt106Δ* double mutant (Li et al., 2008).

The CBF3-dependent localization of Cac1 to centromeres suggested a role for CAF-1 in Cse4–H4 deposition (Sharp et al., 2002). Co-immunoprecipitation experiments confirmed that CAF-1 physically interacts with Cse4, primarily through Cac1 and Cac3. *In vitro* reconstitution assays demonstrated that recombinant CAF-1 can also assemble Cse4-containing nucleosomes on both centromeric and non-centromeric DNA (Hewawasam et al., 2018).

Although Scm3 is the primary chaperone for Cse4 nucleosome assembly, its depletion did not prevent the centromeric incorporation of overexpressed Cse4, implying the involvement of additional chaperones. This hypothesis was supported by the finding that the combined downregulation of Scm3 and absence of CAF-1 (*cac2Δ*) caused a severe growth defect when Cse4 was overexpressed, demonstrating that Scm3 and CAF-1 can independently mediate Cse4 incorporation (Hewawasam et al., 2018) (Figure 5A). CAF-1 may also function under physiological conditions, as it localizes to centromeres throughout the cell cycle and interacts with Cse4 in asynchronous and G2/M-arrested cells, suggesting a replication-independent role in Cse4 nucleosome assembly (Hewawasam et al., 2018).

To assess whether CAF-1 promotes the misincorporation of overexpressed Cse4 in *psh1Δ* cells, CAF-1 subunits were deleted individually. Loss of CAF-1 suppressed the growth defect caused by Cse4 overexpression in *psh1Δ* cells and markedly reduced genome-wide Cse4 incorporation, including at promoters, highly transcribed regions such as the rDNA locus, and subtelomeres. Chromatin-associated Cse4 levels were markedly reduced in *cac2Δ* and *cac2Δ psh1Δ* strains, compared to wild-type yeast and the *psh1Δ* mutant (Hewawasam et al., 2018) (Figure 5A). These results indicated that CAF-1 facilitates Cse4 mislocalization to regions of high nucleosome turnover, thereby disrupting chromatin function and cell growth (Hildebrand and Biggins, 2016). Given that Scm3 protects Cse4 from Psh1-mediated ubiquitination, a similar role for CAF-1 was tested *in vitro* by assessing the polyubiquitination of recombinant Cse4 by Psh1 in the presence or absence of CAF-1. Surprisingly, the presence of CAF-1 enhanced the Psh1-mediated ubiquitination of Cse4. Because CAF-1 does not physically interact with Psh1, it likely binds soluble Cse4 or Cse4–H4, thereby exposing ubiquitination sites and promoting Psh1 access (Hewawasam et al., 2018) (Figure 5A).

#### SUMOylation of Cse4’s HFD is required for deposition by CAF-1 and Scm3

SUMOylation of Cse4 at K65 by Siz1 and Siz2 primes it for polyubiquitination by E2 Ubc4 and the E3 Slx5–Slx6 ligase complex, promoting proteasomal degradation and restricting Cse4 to centromeres (Figure 4B). Cse4 also contains a SUMOylation motif at K215 and K216 within its histone-fold domain. Mutating these residues (K215/216R or K215/216A) reduced Cse4 SUMOylation but also abolished the synthetic lethality observed upon their overexpression in *psh1Δ* cells, a phenotype that resembles that of *cac2Δ psh1Δ* cells overexpressing wild-type Cse4 (Hewawasam et al., 2018). Cse4-K215/216R and Cse4-K215/216A exhibited decreased binding to CAF-1. They showed diminished non-centromeric localization upon overexpression, compared to wild-type Cse4, suggesting that the SUMOylation of K215 and K216 facilitates the CAF-1-dependent misincorporation of Cse4 (Ohkuni et al., 2020) into non-centromeric chromatin. Both variants also showed reduced interaction with the chaperone Scm3, resulting in impaired centromeric localization. Consistently, cells endogenously expressing either Cse4 mutant showed increased chromosome loss compared to those expressing wild-type Cse4, indicating impaired centromere function. All three CAF-1 subunits and Scm3 harbor predicted SUMO-interacting motifs, suggesting that SUMOylation at K215/216 promotes the recruitment and activities of both chaperones toward Cse4 (Ohkuni et al., 2020). This modification is functionally essential, as Scm3-depleted cells overexpressing either Cse4-K215 or Cse4-216 mutants were inviable, a defect that was rescued by overexpressing wild-type Cse4 (Ohkuni et al., 2020) (Figure 4A). Slx5 also harbors a predicted SUMO-interacting motif. Cse4 SUMOylation was markedly reduced in E3 SUMO ligase *siz1Δsiz2Δ* mutant cells and in the K65/215/216R triple mutant, indicating Siz1 and Siz2 as the principal ligases that target these lysines. Despite shared regulation, SUMOylation at K65 and K215/216 serves distinct roles. Overexpression of the Cse4-K215/216R or Cse4-K65/215/216R mutants revealed similarly weak interactions with Scm3 and CAF-1 and similarly defective localization. This demonstrates that SUMOylation at K215/216 promotes chaperone-mediated deposition, whereas SUMOylation at K65 regulates Cse4 turnover (Ohkuni et al., 2020).

#### HIR histone chaperone complex

During transcription and primarily in a replication-independent manner, the HIR complex (Hir1-3 and Hpc2) receives nascent H3–H4 dimers from Asf1 to mediate their deposition into chromatin (Shibahara and Stillman, 1999; Green et al., 2005; Chen B. et al., 2023). The HIR complex is functionally analogous to CAF-1, as both localize independently to centromeres via Ndc10/CBF3. Loss of either HIR or CAF-1 activity impaired centromeric chromatin integrity two-fold, with the *cac1Δ hir1Δ* double mutant exhibiting additive defects, including a 97-fold increase in chromosome loss relative to wild-type yeast (Sharp et al., 2002). Notably, this double mutant displayed reduced centromeric Cse4 levels and increased ectopic deposition, a phenotype absent in the single mutants, indicating that both pathways are required for centromere-specific Cse4 deposition (Sharp et al., 2002; Lopes da Rosa et al., 2011) (Figures 5A,B). In *cac1Δ hir1Δ* cells, endogenous Cse4 is preferentially mislocalized at the +1 and −1 nucleosomes flanking transcription start sites, correlating with reduced histone turnover activity at these regions and increased Cse4 retention in the absence of both complexes. In contrast, coding regions, which exhibit low H3–H4 exchange, were largely unaffected (Dion et al., 2007; Lopes da Rosa et al., 2011).

Deletion of *HIR1* or *HIR2* increased the stability of overexpressed Cse4 by six- and eight-fold, respectively, whereas a loss of *PSH1* in *hir2Δ* cells had no additional effect, indicating that Psh1 mediates Cse4 degradation when the HIR complex is functional. Consistently, *hir2Δ* cells showed increased non-centromeric Cse4 enrichment; however, the kinetochore protein Mtw1 did not co-localize with these sites, demonstrating that ectopic Cse4 incorporation alone is insufficient for kinetochore recruitment (Ciftci-Yilmaz et al., 2018). Cse4 deposition in *hir2Δ* cells occurred primarily at promoters, less frequently in intergenic regions, and rarely within coding sequences, mirroring patterns observed in *psh1Δ* (Hildebrand and Biggins, 2016) and *cac1Δ hir1Δ* mutants (Lopes da Rosa et al., 2011). Because *hir2Δ* and *hir2Δ psh1Δ* strains exhibited similar increases in Cse4 stability, mislocalization, and plasmid loss, the relationship between Cse4, Psh1, and Hir2 was examined. The three proteins were found to interact. However, Psh1–Cse4 binding was reduced in *hir2Δ* cells. Overexpressing Psh1 promoted Cse4 proteolysis in the *hir2Δ* mutant, likely by restoring the interaction with Cse4, suggesting that Hir2 facilitates Psh1-dependent Cse4 turnover (Ciftci-Yilmaz et al., 2018) (Figure 5B).

#### SWI/SNF chromatin-remodeling complex

The SWI/SNF chromatin-remodeling complex functions during transcription and DNA replication (Centore et al., 2020; Bieluszewski et al., 2023; Chen K. et al., 2023) and is enriched at centromeres and pericentromeric regions. Deleting its catalytic ATPase, Snf2, reduced centromeric Cse4 levels and caused Cse4 mislocalization to loci not generally bound by SWI/SNF, while overall Cse4 levels remained comparable to wild-type yeast, indicating that Cse4 mislocalization was solely due to the lack of SWI/SNF activity. *In vitro*, SWI/SNF disassembled Cse4-containing nucleosomes in an ATP- and Nap1-dependent manner while sparing H3 nucleosomes (Gkikopoulos et al., 2011), indicating that SWI/SNF may selectively evict ectopically deposited Cse4 (Furuyama and Biggins, 2007). At centromeres, Cse4 may be protected from eviction by SWI/SNF by kinetochore proteins, the chaperone Scm3, or local post-translational inhibition of SWI/SNF activity. Alternatively, SWI/SNF may counteract the Cse4-evicting activity of the remodeler of the chromatin structure (RSC) complex, which also localizes to centromeres (Hsu et al., 2003) and disassembles Cse4 nucleosomes *in vitro* (Gkikopoulos et al., 2011) (Figures 5C,D).

#### RSC chromatin-remodeling complex

The RSC (remodeler of the structure of chromatin) complex is a yeast-specific and essential member of the SWI/SNF chromatin-remodeling family. Its catalytic ATPase, Sth1, is homologous to Snf2 (Cairns et al., 1996; Ye et al., 2019; Neumann and Wilkins, 2021). Depleting or inactivating Sth1 induced a SAC-mediated G2/M delay and increased centromere accessibility, indicating compromised nucleosome integrity (Tsuchiya et al., 1998). RSC mutants displayed lethal genetic interactions with mutations in *CSE4*, *MIF2*, CBF3 subunits, and the H4 *hhf1-20* allele, which disrupts centromeric chromatin (Meluh et al., 1998; Glowczewski et al., 2000), and with a mutation in the centromeric CDEI element, but not CDEII. Notably, Cse4 overexpression partially suppressed an RSC mutation. Because RSC mutations did not alter the expression of kinetochore proteins, tubulin, or cell cycle regulators (Hsu et al., 2003), its centromeric function is likely direct. Consistent with this, Sth1 was shown to interact with Cse4 and all four core histones but not with kinetochore components. Although RSC occupies centromeres and adjacent regions, centromeric Cse4 levels were unchanged in *sth1-ts* mutants (Hsu et al., 2003). These findings suggest that RSC remodels centromeric nucleosomes after Cse4 deposition, maintaining centromere identity by balancing Cse4–H4 and H3–H4 incorporation, potentially in cooperation with chaperones such as Scm3 and Yta7. Alternatively, RSC may facilitate precise Cse4 nucleosome positioning and promote the bending of the centromeric CDEII sequence element (Figure 5D).

#### SWR1 and INO80 chromatin-remodeling complexes

The SWR1 complex deposits the histone variant H2A.Z (encoded by *HTZ1*) in place of H2A through its Swr1 ATPase and subunit Swc2 (Willhoft et al., 2018). Although ectopic Cse4 accumulates at nucleosomes flanking nucleosome-depleted regions upstream of transcription start sites, which is a pattern similar to that of H2A.Z localization (Hildebrand and Biggins, 2016), this overlap is not mechanistic. ChIP analyses showed that Cse4 enrichment at promoters in *psh1Δ* cells was independent of H2A.Z, as Cse4 levels at promoters were unchanged in the *psh1Δ htz1Δ* double mutant, compared to *psh1Δ* cells, and as H2A.Z occupancy was unaffected by Cse4 overexpression in *psh1Δ* cells (Hildebrand and Biggins, 2016). The loss of SWR1 activity in *swr1Δ psh1Δ* cells caused only a minor increase in Cse4 mislocalization compared to the *psh1Δ* mutant, without altering global H2A.Z levels, indicating that H2A.Z deposition does not drive ectopic Cse4 incorporation. Instead, Cse4 and H2A.Z appear to compete for occupancy at shared genomic regions (Hildebrand and Biggins, 2016).

The INO80 chromatin-remodeling complex regulates transcription, replication, and DNA repair by promoting nucleosome turnover and histone exchange, including H2A.Z removal (Falbo and Shen, 2012; Bowman, 2025). The INO80 ATPase subunit Ino80 provides helicase activity, while component Nhp10 facilitates chromatin interactions (Falbo and Shen, 2012; Bowman, 2025). Reducing INO80 activity (*nhp10Δ*) decreased ectopic Cse4 accumulation, suggesting that INO80 facilitates Cse4 misincorporation following H3 eviction at promoters. Consistently, Cse4 co-immunoprecipitated with Ino80 in a manner that was enhanced in *psh1Δ* cells. Moreover, a deletion of *NHP10* partially suppressed the lethality of *psh1Δ* cells overexpressing Cse4, further supporting a role for INO80 in the toxic misincorporation of excess Cse4 (Hildebrand and Biggins, 2016) (Figure 5E).

#### FACT chromatin-remodeling complex

The FACT (facilitates chromatin transcription) complex opens chromatin to support transcription, DNA replication, and repair. It facilitates the passage of DNA and RNA polymerase by transiently removing H2A–H2B dimers while retaining the (H3–H4)_2_ tetramer and subsequently reassembling nucleosomes to preserve chromatin integrity and the local epigenetic state. In *S. cerevisiae*, FACT consists of the Spt16–Pob3 dimer and is co-immunoprecipitated with Psh1 (Krogan et al., 2002). Conditionally depleting Spt16 led to an approximately twofold increase in Cse4 stability. Although Cse4 overexpression in Spt16-depleted cells caused only a modest increase in soluble Cse4, chromatin incorporation and mislocalization increased to levels comparable to those observed in *psh1Δ* mutants (Deyter and Biggins, 2014). Deleting *NHP6*, which encodes a factor that recruits FACT to DNA (Formosa et al., 2001), did not stabilize Cse4, indicating that recruitment of FACT to nucleosomes is not required for Cse4 degradation (Deyter and Biggins, 2014). Instead, FACT activity may be facilitated by polymerase-induced nucleosome destabilization or redundancy with other histone chaperones, such as the HIR complex (Formosa et al., 2002). These findings suggest that FACT regulates Cse4 not through nucleosome disassembly, but by functioning as a histone chaperone that transiently evicts and redeposits H2A–H2B dimers to maintain chromatin integrity (Figure 2A).

Psh1 interacts with a small subpopulation of FACT via two regions of Spt16: a central domain and a C-terminal region (residues 301–406). Although the Psh1 C-terminus is dispensable for Cse4 binding (Hewawasam et al., 2010; Deyter and Biggins, 2014; Zhou et al., 2021), it is essential for Cse4 ubiquitination (Deyter and Biggins, 2014). Through this C-terminal interaction with Spt16, Psh1 selectively ubiquitinates Cse4 associated with FACT-remodeled nucleosomes. Consistent with this model, deleting the Spt16 C-terminus reduced Cse4 ubiquitination, increased the misincorporation of overexpressed Cse4, and compromised cell viability of Spt16-depleted cells (Deyter and Biggins, 2014). FACT likely facilitates this pathway by transiently destabilizing nucleosomes to expose Cse4, as supported by ChIP analyses showing reduced chromatin recruitment of a Psh1 mutant that lacks its Spt16-interacting C-terminus (Deyter and Biggins, 2014) (Figure 4B).

#### Cdc48 delivers mislocalized polyubiquitinated Cse4 to the proteasome

Multiple genetic screens identified temperature-sensitive (*ts*) mutants of the essential AAA^+^-ATPase Cdc48 as sensitive to Cse4 overexpression; mutations in its cofactors Ufd1 and Npl4 similarly caused Cse4 dosage lethality (Au et al., 2020; Ohkuni et al., 2022). Cdc48 promotes proteasomal degradation by extracting K48-linked polyubiquitinated substrates through ATP hydrolysis (Tsuchiya et al., 2017). Consistent with a role in Cse4 turnover, Cse4 stability increased 1.6-fold in a *cdc48-ts* mutant (Ohkuni et al., 2022). Although total soluble Cse4 levels were unchanged, mutation of Cdc48 or its cofactors led to mislocalization of endogenous Cse4 to euchromatin. In these mutants, Cse4 polyubiquitination was elevated throughout the cell cycle, but it was barely detectable in wild-type cells. Analysis of the centromere-restricted *cse4–Y193A* mutant, which complements *cse4Δ*, revealed markedly reduced polyubiquitination in the *cdc48-ts* mutant background (Ohkuni et al., 2022). Collectively, these findings demonstrate that mislocalized Cse4 is selectively polyubiquitinated and removed from non-centromeric chromatin by the Cdc48–Ufd1–Npl4 complex (Figure 5F).

A gene deletion analysis identified the E3 ligase responsible for polyubiquitinating Cse4 in the *cdc48-ts* mutant. Deletion of *PSH1*, but not *SLX5*, nearly eliminated Cse4 polyubiquitination, although contributions from additional E3 ligases cannot be excluded. Overexpressing Cse4 in the *cdc48-ts psh1Δ* double mutant reduced the occupancy of Cse4 and Cdc48 cofactor Npl4 at non-centromeric regions compared to the *cdc48-ts* single mutant (Ohkuni et al., 2022). These results indicated that Psh1-mediated ubiquitination of Cse4 facilitates the recognition of mislocalized Cse4 by the Npl4–Ufd1–Cdc48 complex (Figure 5F). Following extraction from non-centromeric chromatin, Cdc48 likely delivers ubiquitinated Cse4 to the proteasome. In contrast, centromeric Cse4 may be protected from both Psh1 and Cdc48 by Scm3.

#### Spt4–Spt5 transcription elongation complex

The transcription elongation factor Spt4 forms a complex with Spt5 that directly binds RNA pol II to promote early elongation and regulate transcriptional processivity (Guo et al., 2008). Acting as a regulatory hub, Spt4–Spt5 recruits chromatin remodelers (e.g., Chd1), histone chaperones (e.g., FACT), and histone-modifying complexes (e.g., SAGA and Rpd3S) (Uzun et al., 2021). Through these interactions, Spt4–Spt5 modulates chromatin structure to repress initiation or increase elongation and coordinates transcription with mRNA processing (Hartzog and Fu, 2013). In yeast, Spt4 is required for faithful chromosome segregation and associates broadly with chromatin, including centromeres, telomeres, the silent mating-type locus HMRa, and actively transcribed genes (Basrai et al., 1996). Its centromeric localization depends on the CBF3 complex, while its binding to the other sites does not. Deleting *SPT4* compromised centromeric chromatin integrity and caused an additional mislocalization of Cse4 to pericentromeres, telomeres, and the silent mating-type locus HMRa, while overall intracellular levels of Cse4 remained unaffected, indicating that Spt4–Spt5 restricts Cse4 to centromeres (Crotti and Basrai, 2004). Notably, this mislocalization phenotype resembled that observed in yeast lacking the CAF-1 and HIR histone chaperone activities (Sharp et al., 2002). In *cac1Δ hir1Δ* mutants, Spt4 was specifically lost from centromeres but retained at other genomic sites (Crotti and Basrai, 2004), placing Spt4 downstream of the CAF-1 and HIR in a pathway that ensures proper centromeric nucleosome assembly and Cse4 positioning (Figures 5A,B). Consistent with a centromere-specific role, Spt4 remained at centromeres but was absent from transcribed genes in the RNA pol II mutant *rpb1-1*, demonstrating that its function in centromeric nucleosome assembly is independent of transcription (Crotti and Basrai, 2004).

#### Additional histone chaperones and chromatin remodelers may promote Cse4 deposition at centromeres only

Rtt106 and Asf1 are established H3–H4 chaperones (Das et al., 2010; Bowman et al., 2011). In *psh1Δ* cells overexpressing Cse4, deletion of *RTT106* or *ASF1* failed to rescue growth and instead further reduced viability (Ciftci-Yilmaz et al., 2018; Hewawasam et al., 2018). Deleting either chaperone alone also impaired growth upon Cse4 overexpression, phenocopying the *psh1Δ* mutant (Ciftci-Yilmaz et al., 2018). These defects may reflect disrupted H3 nucleosome assembly and exchange required for transcriptional regulation (Das et al., 2010; Gurard-Levin et al., 2014). Alternatively, Rtt106 and Asf1 may facilitate the removal of mislocalized Cse4 by promoting the disassembly of ectopic Cse4 nucleosomes, directly or via chromatin remodelers. Consistent with this model, loss of Rtt106 or Asf1 increased the ectopic accumulation of Cse4 in *psh1Δ* cells. Rtt106 also interacts with FACT, which cooperates with Psh1 to degrade mislocalized Cse4 (Deyter and Biggins, 2014). Structural studies showed that Asf1 binds the C-terminal region of histone H3 (Natsume et al., 2007). Because these residues are conserved in Cse4, Asf1 may similarly bind the Cse4–H4 dimer, although a direct interaction has not been demonstrated. Supporting this possibility, deleting *ASF1* in H4 mutants that disrupt Scm3 binding and trigger Scm3 proteolysis significantly reduced centromeric Cse4 levels compared to wild-type cells. This indicated that Asf1 can promote Cse4 deposition at centromeres in the absence of Scm3 (Hewawasam et al., 2018), potentially by delivering Cse4–H4 to CAF-1 (Figure 5A).

Purification of the non-ubiquitinatable and hyperstable Cse4-16KR mutant protein (Ranjitkar et al., 2010) identified interactors beyond core kinetochore components, including Rvb1 and Rvb2. These conserved proteins have dual roles: first, as structural/ATPase subunits of the NuA4 histone acetyltransferase complex and of the chromatin-modifying INO80 and SWR1 complexes, and second, as the ATPase components of the R2TP cochaperone complex (comprising the AAA+ ATPases Rvb1p–Rvb2p, Tah1p, and Pih1p in yeast) that facilitates the biogenesis of the above chromatin modifiers and of RNA pol II-associated factors. Consistent with a role in Cse4 function, mutations in *RVB1* and *RVB2* genetically interacted with centromere and kinetochore mutants, such as *cse4-ts* (Costanzo et al., 2016). Their roles may be evolutionarily conserved, as the human orthologs RUVBL1 and RUVBL2 interact with the CENP-A chaperone HJURP (Perpelescu et al., 2015), although a similar interaction between yeast Scm3 and the Rvb proteins has not been demonstrated.

### Low centromere transcription and active cenRNA turnover promote Cse4 deposition at centromeres

Although high RNA pol II activity interferes with kinetochore assembly by physically occluding the centromere, as observed in both budding yeast and humans (Hill and Bloom, 1987; Bergmann et al., 2012), low-level centromere transcription is required for proper kinetochore function (Chan et al., 2012; Catania et al., 2015). The resulting centromeric RNAs (cenRNAs) act as structural scaffolds that recruit and stabilize key kinetochore proteins such as Cse4/CENP-A, Mif2/CENP-C, while promoting the assembly and activity of the chromosome passenger complex (Ipl1–Bir1–Sli15 in *S. cerevisiae*) (Blower, 2016; Caceres-Gutierrez and Herrera, 2017; Talbert and Henikoff, 2018; Corless et al., 2020). This structural role is underscored by the mislocalization of kinetochore proteins following cenRNA depletion by RNase treatment (Wong et al., 2007; Rosic et al., 2014; Blower, 2016). The mechanisms maintaining this finely tuned transcriptional output differ among organisms: regional centromeres (in fission yeast and higher eukaryotes, including humans) are regulated epigenetically through histone modifications and DNA methylation (Bergmann et al., 2012; Westhorpe and Straight, 2014), whereas the point centromeres of *S. cerevisiae* depend on a balance of transcriptional repressors and activators, the incorporation of the histone variant H2A.Z into flanking nucleosomes, and regulated cenRNA turnover (Figure 5E).

#### Production of cenRNAs occurs in the S-phase after centromere replication

In *S. cerevisiae*, cenRNAs, which are produced by RNA pol II, are exceedingly rare, with ∼0.002 molecules per cell in an asynchronous culture (Chen et al., 2019; Ling and Yuen, 2019b; Smurova et al., 2023). They were initially detected upon inhibition of their targeting to the nuclear exosome (Houseley et al., 2007). CenRNA transcription is cell cycle-regulated: transcripts appear in early S-phase, peak in mid-late S-phase (∼0.03 molecules per cell), and then return to basal G1 levels (Chen et al., 2019; Ling and Yuen, 2019b). This transcriptional burst strictly depends on centromere replication in early S-phase, as inactivation of the pre-replication complex abolishes cenRNA production, indicating tight coupling between replication and subsequent transcription (Ling and Yuen, 2019b). The cenRNAs originate from regions outside the core centromere on either DNA strand (Ling and Yuen, 2019b; Hedouin et al., 2022; Smurova et al., 2023). Despite the short centromeric DNA, cenRNAs are highly heterogeneous and unusually long (∼460 nt to >9,000 nt), extending well into pericentromeric regions (Ling and Yuen, 2019a; Smurova et al., 2023). Although the functional relevance of their length remains unclear, cenRNAs may stabilize centromeric chromatin loops (C-loops) by interlacing with chromatin, bound cohesin complexes, and one another. CenRNA production itself could promote local cohesin positioning. Together, these activities would position the centromeric nucleosome at the C-loop apex (Figure 3) to facilitate kinetochore assembly and sister chromatid alignment (Yeh et al., 2008; Stephens et al., 2011; Lawrimore and Bloom, 2019). Following kinetochore formation and sister chromatid bi-orientation, cohesin cleavage and cenRNA degradation may enable C-loop resolution (Figure 3). A comparable mechanism operates at the rDNA array, where long pre-rRNAs maintain sister chromatid cohesion until cohesin cleavage at anaphase entry is followed by rRNA degradation in late anaphase (Sullivan et al., 2004; D’Ambrosio et al., 2008; Iacovella et al., 2015).

#### Antagonistic transcription factors Cbf1 and Fhk2 regulate centromere transcription

The nonessential transcription factor Cbf1 (Cai and Davis, 1990) binds the centromeric CDEI element (Figure 1B) (Mellor et al., 1991), inducing DNA bending around the centromeric nucleosome (Niedenthal et al., 1993; Kent et al., 2004; Steiner and Henikoff, 2015), thereby facilitating the exposure of CDEIII for CBF3 recruitment and kinetochore assembly (Biggins, 2013). In addition to its structural role, Cbf1 suppresses centromere transcription in a cell cycle-dependent manner, as its dissociation in S-phase coincides with a peak in cenRNA levels (Chen et al., 2019). Consistently, deletion of *CBF1* led to a ∼tenfold increase in cenRNA abundance and increased chromosome loss (Chen et al., 2019; Ling and Yuen, 2019b; Hedouin et al., 2022; Smurova et al., 2023). This phenotype was partially rescued by an RNAi-mediated reduction in cenRNA levels (Ling and Yuen, 2019b), underscoring the requirement for tight cenRNA regulation to maintain centromere and kinetochore function (Ling and Yuen, 2019b).

ChIP-qPCR analyses revealed approximately twofold higher centromeric Cse4 levels in *cbf1Δ* cells, particularly in late G1/early S-phase, indicating an altered timing and abundance of Cse4 deposition. This phenotype was attributed to elevated cenRNA levels, as a further twofold increase in cenRNAs achieved by RNase H1 deletion (*rnh1Δ*) in the *cbf1Δ* background did not further affect centromeric Cse4 enrichment (Chen et al., 2019). Moreover, unlike CENP-A in flies, mice, humans, and maize (Caceres-Gutierrez and Herrera, 2017; Talbert and Henikoff, 2018), Cse4 did not co-immunoprecipitate with cenRNAs, indicating that cenRNAs do not promote Cse4 recruitment in budding yeast (Chen et al., 2019).

Cbf1 occupancy at centromeres peaks in G1 and progressively declines through S-phase, inversely correlating with cenRNA accumulation and the recruitment of the DNA helicase Pif1 (Chen et al., 2019). Pif1 displaces DNA-bound proteins and removes RNA from RNA–DNA hybrids (Boule and Zakian, 2007; Zhou et al., 2014). Consistent with this activity, loss of Pif1 (*pif1Δ*) increased centromeric Cbf1 binding, indicating that Pif1 mediates Cbf1 removal to permit centromere transcription (Figure 5E). Deletion of the related DNA helicase Rrm3 did not affect Cbf1 levels at centromeres, demonstrating that Pif1 is specific in clearing Cbf1. Finally, cenRNA regulation by Cbf1 is unidirectional: deletion of RNase H1 increased cenRNA levels without altering Cbf1 occupancy (Chen et al., 2019), confirming that cenRNA accumulation results from, rather than causes, Cbf1 dissociation.

In contrast, the forkhead transcription factor Fkh2 positively regulates cenRNA production, as an *FHK2* deletion reduced cenRNA levels by half (Ling and Yuen, 2019b). However, its precise roles within the centromeric transcription machinery, including interactions with RNA pol II, chromatin remodelers, and histone- or DNA-modifying enzymes, remain undefined. In addition, the transcription factors Ste12 and Dig1 have been reported to repress centromere transcription (Ohkuni and Kitagawa, 2011), although these findings have not been independently confirmed (Ling and Yuen, 2019b).

#### Histone variant H2A.Z represses centromere transcription

The histone variant H2A.Z (Htz1 in *S. cerevisiae*), deposited by the SWR1 chromatin remodeler complex (Keogh et al., 2006), is a key regulator of chromatin dynamics that can either activate or repress transcription in a context-dependent manner. H2A.Z is most often enriched at genes with low expression, where it localizes to nucleosomes flanking nucleosome-depleted regions, such as the +1 nucleosome at repressed promoters. At these sites, H2A.Z can restrict transcription factor binding and RNA pol II progression. At centromeres, H2A.Z localizes to flanking nucleosomes (Albert et al., 2007) (Figure 5E) and functions as a transcriptional repressor: deletion of *HTZ1* doubled cenRNA levels (Ling and Yuen, 2019b) and correlated with transcription initiation from pericentromeric regions (Ling and Yuen, 2019a; Hedouin et al., 2022; Smurova et al., 2023). Although the underlying mechanism remains unclear, repression may involve the exclusion of histone acetyltransferases such as SAGA or restriction of SWI/SNF remodeling activity, thereby maintaining a repressive chromatin state at centromere promoters (Santisteban et al., 2000). Notably, the chromatin remodeler Fun30 was found to be enriched at centromeres, and its deletion reduced H2A.Z levels at periCEN regions while compromising the centromeric chromatin structure. Fun30 may therefore support proper (peri)centromeric organization and faithful chromosome segregation by repressing (peri)centromeric transcription via H2A.Z deposition (Durand-Dubief et al., 2012). However, how Fun30 interacts with other transcriptional regulators and remodelers at centromeres remains unclear.

H2A.Z and Cbf1 repress centromere transcription independently, as evidenced by the additive increase in cenRNA levels in the *htz1Δ cbf1Δ* double mutant, with *cbf1Δ* exerting the stronger effect (Ling and Yuen, 2019b). Consistent with a broader repressive role, H2A.Z may promote local chromatin compaction at centromeres, analogous to its function at the rDNA array (Rogers et al., 2024). All *cbf1Δ*, *htz1Δ*, and *cbf1Δ htz1Δ* mutants exhibited elevated minichromosome loss, most pronounced in the double mutant. In *cbf1Δ htz1Δ* cells, centromeric and cellular levels of Cse4, Scm3, and Mif2 were markedly reduced despite unchanged mRNA levels, suggesting increased post-translational degradation due to defective centromere recruitment, likely resulting from derepressed centromere transcription (Ling and Yuen, 2019b).

#### Kinetochore occupancy reduces centromere transcription

CBF3 assembly and centromere binding are essential for kinetochore formation. In their absence, kinetochores fail to assemble, leading to severe chromosome missegregation because the spindle assembly checkpoint is not active without functional kinetochores (Goh and Kilmartin, 1993; Fraschini et al., 2001; Bouck D. C. and Bloom K. S., 2005). Under these conditions, cenRNA levels increased by up to 100-fold, likely due to unrestrained RNA pol II activity and loss of factors that normally promote cenRNA turnover (Smurova et al., 2023). However, transcription was not completely suppressed by an intact kinetochore, as wild-type centromeres remained transcriptionally active during mitosis (Smurova et al., 2023). This suggested that constitutive low-level RNA pol II activity and a limited supply of cenRNAs may contribute to kinetochore maintenance, integrity, and function during mitosis.

#### Kinase/ATPase Rio1 represses centromere transcription and promotes cenRNA turnover

The conserved and essential kinase/ATPase Rio1 maintains genomic stability in *S. cerevisiae* by repressing transcription at the ribosomal DNA (rDNA) array and centromeres. Rio1 localizes to centromeres during early S-phase, where it represses RNA pol II transcription. Depleting Rio1 markedly increases RNA pol II occupancy at centromeric and pericentromeric regions (Smurova et al., 2023). Although the mechanism of RNA pol II repression remains unclear, it may involve phosphorylation of its C-terminal domain.

Loss of Rio1 profoundly altered centromeric and pericentromeric chromatin by reducing Sir2 levels at centromere-flanking regions by half and by activating numerous cryptic transcription start sites in the pericentromere. These changes likely account for the increase in cenRNA levels observed upon Rio1 depletion (4- to 25-fold, depending on the centromere). Rio1 co-purified the chromatin remodelers SWI/SNF, RSC, and Fun30, suggesting it may restrict RNA pol II access through local chromatin remodeling, potentially also during Cse4 deposition. Beyond transcriptional repression, Rio1 additionally promotes transcript turnover: cen- and pericenRNAs accumulate in Rio1-depleted cells even after thiolutin-mediated transcriptional inhibition, indicating a requirement for Rio1 in both transcriptional silencing and cenRNA degradation (Smurova et al., 2023).

Depleting Rio1 in the TRAMP *trf4Δ* mutant, which can no longer present cenRNAs to the nuclear exosome (Houseley et al., 2007; Ling and Yuen, 2019b), resulted in an additive (three-fold) accumulation of cenRNAs, indicating that Rio1 and the TRAMP-exosome pathway act in parallel. In contrast, depletion of the 5′–3′ exoribonuclease Rat1 also increased the levels of these transcripts, but co-depletion with Rio1 showed no additive effect, suggesting that Rio1 and Rat1 act within the same pathway (Smurova et al., 2023). The impaired transcriptional repression and cenRNA clearance in Rio1-depleted cells disrupted kinetochore integrity. Although *CSE4* mRNA levels remain unchanged, total Cse4 protein levels increased three-fold (suggesting an involvement of Rio1 in Cse4 turnover as well), accompanied by a ∼50% increase in kinetochore-associated Cse4 and aberrant accumulation of Mif2 and Ndc80. These defects indicate compromised kinetochore architecture and account for the six-fold increase in chromosome loss measured in cells with reduced Rio1 levels (Smurova et al., 2023). Rio1’s role in centromere regulation is conserved: depletion of the human ortholog RioK1 similarly elevated cenRNA levels, enhanced kinetochore loading of CENP-A and Ndc80, and promoted chromosomal instability and micronuclei formation (Smurova et al., 2023).

#### Centromeric R-loops prevent Cse4 deposition

R-loops are three-stranded nucleic acid structures that form when nascent RNA hybridizes with its DNA template, displacing the non-template strand and generating a damage-prone single-stranded DNA region (Petermann et al., 2022). In *S. cerevisiae*, Hpr1, a subunit of the TREX (transcription-export) complex, suppresses R-loop formation by facilitating transcription elongation and mRNA processing. TREX may also regulate centromere transcription, as it co-immunoprecipitated with Cse4 (Mishra et al., 2021a). Deletion of *HPR1* resulted in a six-fold increase in centromeric R-loops, which were barely detectable in wild-type cells. This accumulation was suppressed by RNase H1 overexpression (Costantino and Koshland, 2018; Chen et al., 2019; Mishra et al., 2021a), which resolves the RNA–DNA hybrids by degrading the RNA strand. Persistent R-loops in *hpr1Δ* cells led to a 30%–40% reduction in centromeric Cse4 and Scm3 levels, without altering their mRNA abundance, indicating that R-loops hinder their chromatin incorporation. However, histone H3 was enriched at centromeres, suggesting aberrant nucleosome assembly. The reduced centromeric Cse4 levels compromised kinetochore integrity, causing bi-orientation defects and a six-fold increase in chromosome loss, phenotypes that were partially rescued by overexpressing RNase H1 (Mishra et al., 2021a). Genetic analyses further demonstrated that R-loop suppression and transcriptional repression are independent mechanisms of cenRNA control. Crucially, their combined deletion had an additive effect, demonstrating that Cbf1 represses RNA pol II initiation, while RNase H1 degrades centromeric transcripts within R-loops to promote centromere integrity (Chen et al., 2019).

## Discussion

*Saccharomyces cerevisiae* remains an unparalleled model for dissecting conserved mechanisms that safeguard chromosome integrity during cell division. Its genetically tractable haploid genome, combined with deeply conserved chromatin and cell cycle regulatory pathways, enables precise manipulation and systematic interrogation of essential processes that are frequently disrupted in cancer.

Over the past 25 years, studies of the budding yeast centromere and its defining histone H3 variant, Cse4, have uncovered an exceptionally sophisticated regulatory framework that enforces the exclusive localization of Cse4 to centromeres, an understanding unmatched in most eukaryotic systems. Key advances include the definition of Cse4–H4 dimerization interfaces, the role of the Cse4 histone-fold domain in centromeric DNA recognition, and the identification of specialized chaperones and chromatin remodelers that govern Cse4 deposition and kinetochore assembly, establishing yeast as a benchmark system for elucidating centromere identity and maintenance. Genetic interaction screens, particularly those leveraging Cse4 overexpression, have revealed SUMO- and ubiquitin-based pathways that, in concert with the proteasome, prevent ectopic Cse4 accumulation, thereby suppressing global transcriptional dysregulation. Strikingly, the continued degradation of a non-ubiquitinatable Cse4 mutant demonstrates that Cse4 turnover involves even more extensive and redundant activities that remain poorly understood.

**FIGURE 6:**
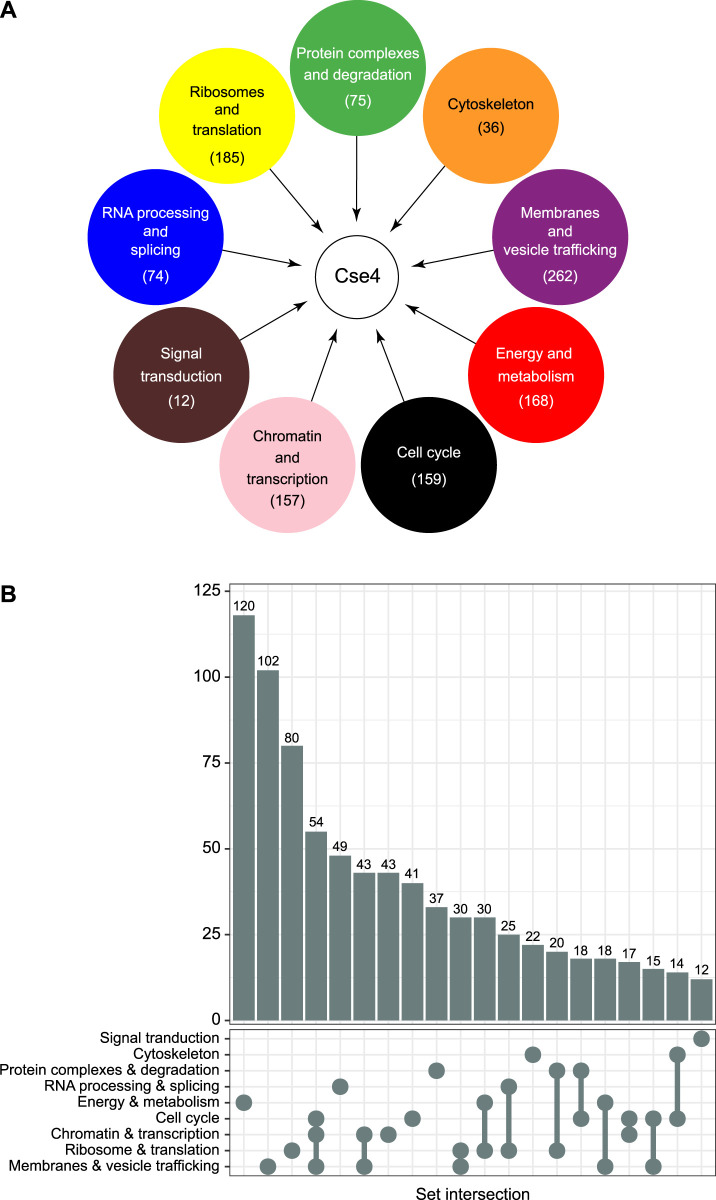
Cse4 protein interactions and genetic networks. **(A)** Functional grouping of proteins that physically interact with Cse4 (identified by affinity purification and mass spectrometry) and/or show negative genetic interactions with Cse4. Genetic interactions were identified through dosage lethality screens using SGA arrays or collections of essential temperature-sensitive strains, as well as from interactions with Cse4 mutations (Hewawasam et al., 2010; 2014; Ranjitkar et al., 2010; Deyter and Biggins, 2014; Ohkuni et al., 2016; Ciftci-Yilmaz et al., 2018; Au et al., 2020; Eisenstatt et al., 2020; Eisenstatt et al., 2021; Oughtred et al., 2021; Engel et al., 2025). The number of proteins in each group is indicated in parentheses. **(B)** Gene Ontology (GO)-term-based classification of the proteins that physically and/or genetically associate with Cse4. The chart indicates the number of proteins belonging to a single GO category (single dot) or multiple categories (connected dots).

The coordination, hierarchy, and temporal control of Cse4 post-translational modifications, especially phosphorylation and its opposing phosphatase activities, remain major unresolved questions, representing a critical gap in our understanding of Cse4 homeostasis. Addressing these challenges will require an integrated strategy that combines high-throughput genetic screens, quantitative proteomics, biochemical reconstitution, and molecular genetics. Existing datasets already provide a powerful foundation: more than 1,100 proteins have been identified as physical or genetic interactors of Cse4, spanning diverse cellular pathways including transcriptional regulation, proteostasis, metabolism, membrane trafficking, and signal transduction (Figure 6). These findings reveal extensive, previously unrecognized regulatory nodes that link centromere integrity to broader cellular maintenance processes.

Despite more than four decades of centromere research, the molecular complexity governing Cse4 function, kinetochore assembly, and chromosome segregation is only beginning to be fully appreciated. Importantly, misregulation of CENP-A, the human ortholog of Cse4, is increasingly recognized as a force driving chromosomal instability and aneuploidy in cancer. By leveraging the experimental power of yeast to define fundamental regulatory principles, this work offers a scalable framework for uncovering conserved vulnerabilities in centromere regulation. These insights are poised to inform translational strategies targeting drug-induced chromosomal instability in aggressive, treatment-resistant cancers.
