# Supplementary material for: Keeping it centered: decoding the activities that regulate yeast histone H3 variant Cse4 and confine it to centromeres
Source: Front Cell Dev Biol. 2026 Apr 1;14:1782273. doi: 10.3389/fcell.2026.1782273 (PMC13079627; doi:10.3389/fcell.2026.1782273)
Supplement: Supplementary file 1 [file Supplementaryfile1.pdf]

Supplemental Table 1 - Functional Grouping and Description of *S. cerevisiae* Genes and Their Human Orthologs

Functional group	Yeast gene	Human ortholog	Human gene name	Function (Alliance of Genome Resources)
Centromere / kinetochore & chromosome segregation	*AME1*	*CENP-U*	centromere protein U	The centromere is a specialized chromatin domain, present throughout the cell cycle, that acts as a platform on which the transient assembly of the kinetochore occurs during mitosis. All active centromeres are characterized by the presence of long arrays of nucleosomes in which CENPA (MIM 117139) replaces histone H3 (see MIM 601128). MLF1IP, or CENPU, is an additional factor required for centromere assembly (Foltz et al., 2006 [PubMed 16622419]).[supplied by OMIM, Mar 2008]
Histone chaperones & assembly regulators	*ASF1*	*ASF1A*	anti-silencing function 1A histone chaperone	This gene encodes a member of the H3/H4 family of histone chaperone proteins and is similar to the anti-silencing function-1 gene in yeast. The protein is a key component of a histone donor complex that functions in nucleosome assembly. It interacts with histones H3 and H4, and functions together with a chromatin assembly factor during DNA replication and repair. [provided by RefSeq, Jul 2008]
Spindle & microtubule regulators	*BIK1*	*CLIP1*	CAP-Gly domain containing linker protein 1	The protein encoded by this gene links endocytic vesicles to microtubules. This gene is highly expressed in Reed-Sternberg cells of Hodgkin disease. Several transcript variants encoding different isoforms have been found for this gene. [provided by RefSeq, Oct 2011]
Spindle & microtubule regulators	*BIM1*	*MAPRE1*	microtubule associated protein RP/EB family member 1	The protein encoded by this gene was first identified by its binding to the APC protein which is often mutated in familial and sporadic forms of colorectal cancer. This protein localizes to microtubules, especially the growing ends, in interphase cells. During mitosis, the protein is associated with the centrosomes and spindle microtubules. The protein also associates with components of the dynactin complex and the intermediate chain of cytoplasmic dynein. Because of these associations, it is thought that this protein is involved in the regulation of microtubule structures and chromosome stability. This gene is a member of the RP/EB family. [provided by RefSeq, Jul 2008]
Histone chaperones & assembly regulators	*CAC1 (RLF2)*	*CHAF1A*	chromatin assembly factor 1 subunit A	Chromatin assembly factor I (CAF1) is a nuclear complex consisting of p50, p60 (CHAF1B; MIM 601245), and p150 (CHAF1A) subunits that assembles histone octamers onto replicating DNA in vitro (Kaufman et al., 1995 [PubMed 7600578]).[supplied by OMIM, Mar 2008]
Histone chaperones & assembly regulators	*CAC2*	*CHAF1B*	chromatin assembly factor 1 subunit B	Chromatin assembly factor I (CAF-I) is required for the assembly of histone octamers onto newly-replicated DNA. CAF-I is composed of three protein subunits, p50, p60, and p150. The protein encoded by this gene corresponds to the p60 subunit and is required for chromatin assembly after replication. The encoded protein is differentially phosphorylated in a cell cycle-dependent manner. In addition, it is normally found in the nucleus except during mitosis, when it is released into the cytoplasm. This protein is a member of the WD-repeat HIR1 family and may also be involved in DNA repair. [provided by RefSeq, Jul 2008]
Histone chaperones & assembly regulators	*CAC3 (MSI1)*	*RBBP4*	RB binding protein 4, chromatin remodeling factor	This gene encodes a ubiquitously expressed nuclear protein which belongs to a highly conserved subfamily of WD-repeat proteins. It is present in protein complexes involved in histone acetylation and chromatin assembly. It is part of the Mi-2 complex which has been implicated in chromatin remodeling and transcriptional repression associated with histone deacetylation. This encoded protein is also part of co-repressor complexes, which is an integral component of transcriptional silencing. It is found among several cellular proteins that bind directly to retinoblastoma protein to regulate cell proliferation. This protein also seems to be involved in transcriptional repression of E2F-responsive genes. Three transcript variants encoding different isoforms have been found for this gene. [provided by RefSeq, Sep 2008]
Centromere / kinetochore & chromosome segregation	*CBF1*	*USF1*	upstream transcription factor 1	This gene encodes a member of the basic helix-loop-helix leucine zipper family, and can function as a cellular transcription factor. The encoded protein can activate transcription through pyrimidine-rich initiator (Inr) elements and E-box motifs. This gene has been linked to familial combined hyperlipidemia (FCHL). Alternative splicing of this gene results in multiple transcript variants. A related pseudogene has been defined on chromosome 21. [provided by RefSeq, Feb 2013]
Centromere / kinetochore & chromosome segregation	*CEP3 (CBF3)*	—	—	—
RNA processing & ribosome biogenesis	*CBF5*	*DKC1*	dyskerin pseudouridine synthase 1	This gene functions in two distinct complexes. It plays an active role in telomerase stabilization and maintenance, as well as recognition of snoRNAs containing H/ACA sequences which provides stability during biogenesis and assembly into H/ACA small nucleolar RNA ribonucleoproteins (snoRNPs). This gene is highly conserved and widely expressed, and may play additional roles in nucleo-cytoplasmic shuttling, DNA damage response, and cell adhesion. Mutations have been associated with X-linked dyskeratosis congenita. Alternative splicing results in multiple transcript variants. [provided by RefSeq, Jan 2014]

Supplemental Table 1 - Functional Grouping and Description of *S. cerevisiae* Genes and Their Human Orthologs

Functional group	Yeast gene	Human ortholog	Human gene name	Function (Alliance of Genome Resources)
Cell-cycle kinases & regulators	*CDC14*	*CDC14B*	cell division cycle 14B	The protein encoded by this gene is a member of the dual specificity protein tyrosine phosphatase family. This protein is highly similar to *Saccharomyces cerevisiae* Cdc14, a protein tyrosine phosphatase involved in the exit of cell mitosis and initiation of DNA replication, which suggests the role in cell cycle control. This protein has been shown to interact with and dephosphorylates tumor suppressor protein p53, and is thought to regulate the function of p53. Alternative splice of this gene results in 3 transcript variants encoding distinct isoforms. [provided by RefSeq, Jul 2008]
Ubiquitin / SUMO system & protein turnover	*CDC34 (UBC3)*	*UBE2G1*	ubiquitin conjugating enzyme E2 G1	The modification of proteins with ubiquitin is an important cellular mechanism for targeting abnormal or short-lived proteins for degradation. Ubiquitination involves at least three classes of enzymes: ubiquitin-activating enzymes, or E1s, ubiquitin-conjugating enzymes, or E2s, and ubiquitin-protein ligases, or E3s. This gene encodes a member of the E2 ubiquitin-conjugating enzyme family and catalyzes the covalent attachment of ubiquitin to other proteins. The protein may be involved in degradation of muscle-specific proteins. [provided by RefSeq, Jul 2008]
Ubiquitin / SUMO system & protein turnover	*CDC4*	*FBXW7*	F-box and WD repeat domain containing 7	This gene encodes a member of the F-box protein family which is characterized by an approximately 40 amino acid motif, the F-box. The F-box proteins constitute one of the four subunits of ubiquitin protein ligase complex called SCFs (SKP1-cullin-F-box), which function in phosphorylation-dependent ubiquitination. The F-box proteins are divided into 3 classes: Fbws containing WD-40 domains, Fbls containing leucine-rich repeats, and Fbxs containing either different protein-protein interaction modules or no recognizable motifs. The protein encoded by this gene was previously referred to as FBX30, and belongs to the Fbws class; in addition to an F-box, this protein contains 7 tandem WD40 repeats. This protein binds directly to cyclin E and probably targets cyclin E for ubiquitin-mediated degradation. Mutations in this gene are detected in ovarian and breast cancer cell lines, implicating the gene's potential role in the pathogenesis of human cancers. Multiple transcript variants encoding different isoforms have been found for this gene. [provided by RefSeq, Mar 2012]
Ubiquitin / SUMO system & protein turnover	*CDC48*	*VCP*	valosin containing protein	This gene encodes a member of the AAA ATPase family of proteins. The encoded protein plays a role in protein degradation, intracellular membrane fusion, DNA repair and replication, regulation of the cell cycle, and activation of the NF-kappa B pathway. This protein forms a homohexameric complex that interacts with a variety of cofactors and extracts ubiquitinated proteins from lipid membranes or protein complexes. Mutations in this gene cause IBMPFD (inclusion body myopathy with paget disease of bone and frontotemporal dementia), ALS (amyotrophic lateral sclerosis) and Charcot-Marie-Tooth disease in human patients. [provided by RefSeq, Aug 2017]
Cell-cycle kinases & regulators	*CDC5*	*PLK1*	polo like kinase 1	The Ser/Thr protein kinase encoded by this gene belongs to the CDC5/Polo subfamily. It is highly expressed during mitosis and elevated levels are found in many different types of cancer. Depletion of this protein in cancer cells dramatically inhibited cell proliferation and induced apoptosis; hence, it is a target for cancer therapy. [provided by RefSeq, Sep 2015]
DNA replication & fork progression	*CDC6*	*CDC6*	cell division cycle 6	The protein encoded by this gene is highly similar to *Saccharomyces cerevisiae* Cdc6, a protein essential for the initiation of DNA replication. This protein functions as a regulator at the early steps of DNA replication. It localizes in cell nucleus during cell cyle G1, but translocates to the cytoplasm at the start of S phase. The subcellular translocation of this protein during cell cyle is regulated through its phosphorylation by Cdks. Transcription of this protein was reported to be regulated in response to mitogenic signals through transcriptional control mechanism involving E2F proteins. [provided by RefSeq, Jul 2008]
DNA replication & fork progression	*CDC7*	*CDC7*	cell division cycle 7	This gene encodes a cell division cycle protein with kinase activity that is critical for the G1/S transition. The yeast homolog is also essential for initiation of DNA replication as cell division occurs. Overexpression of this gene product may be associated with neoplastic transformation for some tumors. Multiple alternatively spliced transcript variants that encode the same protein have been detected. [provided by RefSeq, Aug 2008]
Centromere / kinetochore & chromosome segregation	*CHL4*	*CENP-N*	centromere protein N	The protein encoded by this gene forms part of the nucleosome-associated complex and is important for kinetochore assembly. It is bound to kinetochores during S phase and G2 and recruits other proteins to the centromere. Pseudogenes of this gene are located on chromosome 2. Alternative splicing results in multiple transcript variants that encode different protein isoforms. [provided by RefSeq, Jul 2012]
Histone chaperones & assembly regulators	*CHZ1*	—	—	—

Supplemental Table 1 - Functional Grouping and Description of *S. cerevisiae* Genes and Their Human Orthologs

Functional group	Yeast gene	Human ortholog	Human gene name	Function (Alliance of Genome Resources)
Cell-cycle kinases & regulators	*CKA1*	*CSNK2A1*	casein kinase 2 alpha 1	Casein kinase II is a serine/threonine protein kinase that phosphorylates acidic proteins such as casein. It is involved in various cellular processes, including cell cycle control, apoptosis, and circadian rhythm. The kinase exists as a tetramer and is composed of an alpha, an alpha-prime, and two beta subunits. The alpha subunits contain the catalytic activity while the beta subunits undergo autophosphorylation. The protein encoded by this gene represents the alpha subunit. Multiple transcript variants encoding different protein isoforms have been found for this gene. [provided by RefSeq, Apr 2018]
Cell-cycle kinases & regulators	*CKA2*	*CSNK2A2*	casein kinase 2 alpha 2	This gene encodes the alpha', or alpha 2, catalytic subunit of the protein kinase enzyme, casein kinase 2 (CK2). Casein kinase 2 is a serine/threonine protein kinase that phosphorylates acidic proteins such as casein. It is involved in various cellular processes, including cell cycle control, apoptosis, and circadian rhythms. This heterotetrameric kinase includes two catalytic subunits, either alpha or alpha', and two regulatory beta subunits. The closely related gene paralog encoding the alpha, or alpha 1 subunit (CSNK2A1, Gene ID: 1457) is found on chromosome 20. An intronic variant in this gene (alpha 2) may be associated with leukocyte telomere length in a South Asian population. A related transcribed pseudogene is found on chromosome 11. [provided by RefSeq, Aug 2017]
Cell-cycle kinases & regulators	*CKB1*	*CSNK2B*	casein kinase 2 beta	This gene encodes the beta subunit of casein kinase II, a ubiquitous protein kinase which regulates metabolic pathways, signal transduction, transcription, translation, and replication. The enzyme is composed of three subunits, alpha, alpha prime and beta, which form a tetrameric holoenzyme. The alpha and alpha prime subunits are catalytic, while the beta subunit serves regulatory functions. The enzyme localizes to the endoplasmic reticulum and the Golgi apparatus. Two transcript variants encoding different isoforms have been found for this gene. [provided by RefSeq, Sep 2013]
Cell-cycle kinases & regulators	*CKB2*	*CSNK2B*	casein kinase 2 beta	This gene encodes the beta subunit of casein kinase II, a ubiquitous protein kinase which regulates metabolic pathways, signal transduction, transcription, translation, and replication. The enzyme is composed of three subunits, alpha, alpha prime and beta, which form a tetrameric holoenzyme. The alpha and alpha prime subunits are catalytic, while the beta subunit serves regulatory functions. The enzyme localizes to the endoplasmic reticulum and the Golgi apparatus. Two transcript variants encoding different isoforms have been found for this gene. [provided by RefSeq, Sep 2013]
Centromere / kinetochore & chromosome segregation	*CSE4*	*CENP-A*	centromere protein A	Centromeres are the differentiated chromosomal domains that specify the mitotic behavior of chromosomes. This gene encodes a centromere protein which contains a histone H3 related histone fold domain that is required for targeting to the centromere. Centromere protein A is proposed to be a component of a modified nucleosome or nucleosome-like structure in which it replaces 1 or both copies of conventional histone H3 in the (H3-H4)2 tetrameric core of the nucleosome particle. The protein is a replication-independent histone that is a member of the histone H3 family. Alternative splicing results in multiple transcript variants encoding distinct isoforms. [provided by RefSeq, Nov 2015]
Centromere / kinetochore & chromosome segregation	*CTF13*	—	—	—
Centromere / kinetochore & chromosome segregation	*CTF19*	*CENP-P*	centromere protein P	CENPP is a subunit of a CENPH (MIM 605607)-CENPI (MIM 300065)-associated centromeric complex that targets CENPA (MIM 117139) to centromeres and is required for proper kinetochore function and mitotic progression (Okada et al., 2006 [PubMed 16622420]).[supplied by OMIM, Mar 2008]
Centromere / kinetochore & chromosome segregation	*CTF3*	*CENP-I*	centromere protein I	This gene encodes a centromere protein that is a component of the CENPA-NAC (nucleosome-associated) complex. This complex is critical for accurate chromosome alignment and segregation and it ensures proper mitotic progression. This protein regulates the recruitment of kinetochore-associated proteins that are required to generate the spindle checkpoint signal. The product of this gene is involved in the response of gonadal tissues to follicle-stimulating hormone. Mutations in this gene may be involved in human X-linked disorders of gonadal development and gametogenesis. Alternate splicing results in multiple transcript variants. A pseudogene of this gene is found on chromosome 13. [provided by RefSeq, Jan 2016]
Centromere / kinetochore & chromosome segregation	*DAM1*	subunit of Ska complex	—	—
DNA replication & fork progression	*DBF4*	*DBF4*	DBF4-CDC7 kinase regulatory subunit	Predicted to enable protein serine/threonine kinase activator activity. Predicted to be involved in positive regulation of nuclear cell cycle DNA replication and regulation of cell cycle phase transition. Located in nuclear body. [provided by Alliance of Genome Resources, Apr 2025]

Supplemental Table 1 - Functional Grouping and Description of *S. cerevisiae* Genes and Their Human Orthologs

Functional group	Yeast gene	Human ortholog	Human gene name	Function (Alliance of Genome Resources)
Ubiquitin / SUMO system & protein turnover	*DOA1*	*PLAA*	phospholipase A2 activating protein	Predicted to enable ubiquitin binding activity. Involved in cellular response to lipopolysaccharide; macroautophagy; and positive regulation of phospholipase A2 activity. Located in cytoplasm; extracellular exosome; and nucleus. [provided by Alliance of Genome Resources, Apr 2025]
Centromere / kinetochore & chromosome segregation	*DSN1*	*DSN1*	DSN1 component of MIS12 kinetochore complex	This gene encodes a kinetochore protein that functions as part of the minichromosome instability-12 centromere complex. The encoded protein is required for proper kinetochore assembly and progression through the cell cycle. Alternative splicing results in multiple transcript variants. [provided by RefSeq, Feb 2009]
Histone modifiers & silencing enzymes	*ESA1*	*KAT5*	lysine acetyltransferase 5	The protein encoded by this gene belongs to the MYST family of histone acetyl transferases (HATs) and was originally isolated as an HIV-1 TAT-interactive protein. HATs play important roles in regulating chromatin remodeling, transcription and other nuclear processes by acetylating histone and nonhistone proteins. This protein is a histone acetylase that has a role in DNA repair and apoptosis and is thought to play an important role in signal transduction. Alternative splicing of this gene results in multiple transcript variants. [provided by RefSeq, Jul 2008]
Transcription machinery & regulators	*FKH2*	*FOXK1*	forkhead box K1	Enables 14-3-3 protein binding activity; DNA-binding transcription repressor activity, RNA polymerase II-specific; and transcription cis-regulatory region binding activity. Involved in several processes, including intracellular glucose homeostasis; negative regulation of autophagy; and regulation of DNA-templated transcription. Located in cytoplasm and nucleus. [provided by Alliance of Genome Resources, Apr 2025]
Transcription machinery & regulators	*FKH2*	*FOXK2*	forkhead box K2	The protein encoded by this gene contains a fork head DNA binding domain. This protein can bind to the purine-rich motifs of the HIV long terminal repeat (LTR), and to the similar purine-rich motif in the interleukin 2 (IL2) promoter. It may be involved in the regulation of viral and cellular promoter elements. [provided by RefSeq, Jul 2008]
RNA processing & ribosome biogenesis	*FPR3*	—	—	—
RNA processing & ribosome biogenesis	*FPR4*	—	—	—
Chromatin remodelers & ATPases	*FUN30*	*SMARCAD1*	SNF2 related chromatin remodeling ATPase with DExD box 1	This gene encodes a member of the SNF subfamily of helicase proteins. The encoded protein plays a critical role in the restoration of heterochromatin organization and propagation of epigenetic patterns following DNA replication by mediating histone H3/H4 deacetylation. Mutations in this gene are associated with adermatoglyphia. Alternatively spliced transcript variants encoding multiple isoforms have been observed for this gene. [provided by RefSeq, Dec 2011]
Histone modifiers & silencing enzymes	*GCN5*	*KAT2A*	lysine acetyltransferase 2A	KAT2A, or GCN5, is a histone acetyltransferase (HAT) that functions primarily as a transcriptional activator. It also functions as a repressor of NF-kappa-B (see MIM 164011) by promoting ubiquitination of the NF-kappa-B subunit RELA (MIM 164014) in a HAT-independent manner (Mao et al., 2009 [PubMed 19339690]).[supplied by OMIM, Sep 2009]
Core histones & histone variants	*HHF1*	*H4C16, H4C15, H4C1, H4C4, H4C6, H4C12, H4C11, H4C3, H4C8, H4C2, H4C5, H4C13, H4C9, H4C14*	*H4 histone family*	Histones are basic nuclear proteins that are responsible for the nucleosome structure of the chromosomal fiber in eukaryotes. Two molecules of each of the four core histones (H2A, H2B, H3, and H4) form an octamer, around which approximately 146 bp of DNA is wrapped in repeating units, called nucleosomes. The linker histone, H1, interacts with linker DNA between nucleosomes and functions in the compaction of chromatin into higher order structures.
Core histones & histone variants	*HHF2*	*H4C16, H4C15, H4C1, H4C4, H4C6, H4C12, H4C11, H4C3, H4C8, H4C2, H4C5, H4C13, H4C9, H4C14*		
Core histones & histone variants	*HHT1*	*H3C14, H3C15, H3C13, H3-7, H3-5, H3Y2, H3Y1, H3-3A, H3-3B, H3C1, H3C4, H3C3, H3C6, H3C11, H3C8, H3C7, H3C12, H3C10, H3C2, H3-4*	*H3 histone family*	

Supplemental Table 1 - Functional Grouping and Description of *S. cerevisiae* Genes and Their Human Orthologs

Functional group	Yeast gene	Human ortholog	Human gene name	Function (Alliance of Genome Resources)
Core histones & histone variants	*HHT2*	*H3C14, H3C15, H3C13, H3-7, H3-5, H3Y2, H3Y1, H3-3A, H3-3B, H3C1, H3C4, H3C3, H3C6, H3C11, H3C8, H3C7, H3C12, H3C10, H3C2, H3-4*		
Histone chaperones & assembly regulators	*HIR1*	*HIRA*	histone cell cycle regulator	This gene encodes a histone chaperone that preferentially places the variant histone H3.3 in nucleosomes. Orthologs of this gene in yeast, flies, and plants are necessary for the formation of transcriptionally silent heterochromatin. This gene plays an important role in the formation of the senescence-associated heterochromatin foci. These foci likely mediate the irreversible cell cycle changes that occur in senescent cells. It is considered the primary candidate gene in some haploinsufficiency syndromes such as DiGeorge syndrome, and insufficient production of the gene may disrupt normal embryonic development. [provided by RefSeq, Jul 2008]
Histone chaperones & assembly regulators	*HIR2*	*HIRA*	histone cell cycle regulator	This gene encodes a histone chaperone that preferentially places the variant histone H3.3 in nucleosomes. Orthologs of this gene in yeast, flies, and plants are necessary for the formation of transcriptionally silent heterochromatin. This gene plays an important role in the formation of the senescence-associated heterochromatin foci. These foci likely mediate the irreversible cell cycle changes that occur in senescent cells. It is considered the primary candidate gene in some haploinsufficiency syndromes such as DiGeorge syndrome, and insufficient production of the gene may disrupt normal embryonic development. [provided by RefSeq, Jul 2008]
Histone chaperones & assembly regulators	*HIR3*	*CABIN1*	calcineurin binding protein 1	Calcineurin plays an important role in the T-cell receptor-mediated signal transduction pathway. The protein encoded by this gene binds specifically to the activated form of calcineurin and inhibits calcineurin-mediated signal transduction. The encoded protein is found in the nucleus and contains a leucine zipper domain as well as several PEST motifs, sequences which confer targeted degradation to those proteins which contain them. Alternative splicing results in multiple transcript variants encoding two different isoforms. [provided by RefSeq, Jan 2011]
Histone modifiers & silencing enzymes	*HMT1*	*PRMT1*	protein arginine methyltransferase 1	This gene encodes a member of the protein arginine N-methyltransferase (PRMT) family. Post-translational modification of target proteins by PRMTs plays an important regulatory role in many biological processes, whereby PRMTs methylate arginine residues by transferring methyl groups from S-adenosyl-L-methionine to terminal guanidino nitrogen atoms. The encoded protein is a type I PRMT and is responsible for the majority of cellular arginine methylation activity. Increased expression of this gene may play a role in many types of cancer. Alternatively spliced transcript variants encoding multiple isoforms have been observed for this gene, and a pseudogene of this gene is located on the long arm of chromosome 5. [provided by RefSeq, Dec 2011]
Histone chaperones & assembly regulators	*HPC2*	*UBN1*	ubinuclein 1	Cellular senescence is a hallmark of tumor suppression and tissue aging. Senescent cells contain domains of heterochromatin, called senescence-associated heterochromatin foci (SAHF), that repress proliferation-promoting genes. The protein encoded by this gene binds to proliferation-promoting genes and is required for SAHF formation, enhancing methylation of histone H3. [provided by RefSeq, Oct 2016]
Transcription machinery & regulators	*HPR1*	*THOC1*	THO complex subunit 1	Predicted to enable DNA binding activity and RNA binding activity. Involved in mRNA export from nucleus. Located in chromosome, telomeric region; cytoplasm; and nuclear speck. Part of THO complex part of transcription export complex. Implicated in autosomal dominant nonsyndromic deafness 86. [provided by Alliance of Genome Resources, Apr 2025]
Ubiquitin / SUMO system & protein turnover	*HRT1 (RBX1)*	*RBX1*	ring-box 1	This locus encodes a RING finger-like domain-containing protein. The encoded protein interacts with cullin proteins and likely plays a role in ubiquitination processes necessary for cell cycle progression. This protein may also affect protein turnover. Related pseudogenes exist on chromosomes 2 and 5.[provided by RefSeq, Sep 2010]
Cell-cycle kinases & regulators	*HSL7*	*PRMT5*	protein arginine methyltransferase 5	This gene encodes an enzyme that belongs to the methyltransferase family. The encoded protein catalyzes the transfer of methyl groups to the amino acid arginine, in target proteins that include histones, transcriptional elongation factors and the tumor suppressor p53. This gene plays a role in several cellular processes, including transcriptional regulation, and the assembly of small nuclear ribonucleoproteins. A pseudogene of this gene has been defined on chromosome 4. Alternative splicing results in multiple transcript variants encoding different isoforms. [provided by RefSeq, Sep 2015]

Supplemental Table 1 - Functional Grouping and Description of *S. cerevisiae* Genes and Their Human Orthologs

Functional group	Yeast gene	Human ortholog	Human gene name	Function (Alliance of Genome Resources)
Core histones & histone variants	*HTZ1*	*H2AZ2*	H2A.Z variant histone 2	Histones are basic nuclear proteins that are responsible for the nucleosome structure of the chromosomal fiber in eukaryotes. Nucleosomes consist of approximately 146 bp of DNA wrapped around a histone octamer composed of pairs of each of the four core histones (H2A, H2B, H3, and H4). The chromatin fiber is further compacted through the interaction of a linker histone, H1, with the DNA between the nucleosomes to form higher order chromatin structures. This gene encodes a replication-independent histone that is a member of the histone H2A family. Several transcript variants encoding different isoforms, have been identified for this gene. [provided by RefSeq, Oct 2015]
Centromere / kinetochore & chromosome segregation	*IML3*	*CENP-L*	centromere protein L	CENPL is a subunit of a CENPH (MIM 605607)-CENPI (MIM 300065)-associated centromeric complex that targets CENPA (MIM 117139) to centromeres and is required for proper kinetochore function and mitotic progression (Okada et al., 2006) [PubMed 16622420].[supplied by OMIM, Mar 2008]
Chromatin remodelers & ATPases	*INO80*	*INO80*	INO80 complex ATPase subunit	This gene encodes a subunit of the chromatin remodeling complex, which is classified into subfamilies depending on sequence features apart from the conserved ATPase domain. This protein is the catalytic ATPase subunit of the INO80 chromatin remodeling complex, which is characterized by a DNA-binding domain. This protein is proposed to bind DNA and be recruited by the YY1 transcription factor to activate certain genes. Alternative splicing results in multiple transcript variants. [provided by RefSeq, Aug 2013]
Spindle & microtubule regulators	*IPL1*	*AURKB*	aurora kinase B	This gene encodes a member of the aurora kinase subfamily of serine/threonine kinases. The genes encoding the other two members of this subfamily are located on chromosomes 19 and 20. These kinases participate in the regulation of alignment and segregation of chromosomes during mitosis and meiosis through association with microtubules. A pseudogene of this gene is located on chromosome 8. Alternatively spliced transcript variants have been found for this gene. [provided by RefSeq, Sep 2015]
Spindle & microtubule regulators	*KIP1*	*KIF11*	kinesin family member 11	This gene encodes a motor protein that belongs to the kinesin-like protein family. Members of this protein family are known to be involved in various kinds of spindle dynamics. The function of this gene product includes chromosome positioning, centrosome separation and establishing a bipolar spindle during cell mitosis. [provided by RefSeq, Jul 2008]
Cell-cycle kinases & regulators	*MCK1*	*GSK3B*	glycogen synthase kinase 3 beta	The protein encoded by this gene is a serine-threonine kinase belonging to the glycogen synthase kinase subfamily. It is a negative regulator of glucose homeostasis and is involved in energy metabolism, inflammation, ER-stress, mitochondrial dysfunction, and apoptotic pathways. Defects in this gene have been associated with Parkinson disease and Alzheimer disease. [provided by RefSeq, Aug 2017]
DNA replication & fork progression	*MCM16*	*CENP-H*	centromere protein H	Centromere and kinetochore proteins play a critical role in centromere structure, kinetochore formation, and sister chromatid separation. The protein encoded by this gene colocalizes with inner kinetochore plate proteins CENP-A and CENP-C in both interphase and metaphase. It localizes outside of centromeric heterochromatin, where CENP-B is localized, and inside the kinetochore corona, where CENP-E is localized during prometaphase. It is thought that this protein can bind to itself, as well as to CENP-A, CENP-B or CENP-C. Multimers of the protein localize constitutively to the inner kinetochore plate and play an important role in the organization and function of the active centromere-kinetochore complex. [provided by RefSeq, Jul 2008]
Centromere / kinetochore & chromosome segregation	*MCM21*	*CENP-O*	centromere protein O	This gene encodes a component of the interphase centromere complex. The encoded protein is localized to the centromere throughout the cell cycle and is required for bipolar spindle assembly, chromosome segregation and checkpoint signaling during mitosis. Alternatively spliced transcript variants encoding multiple protein isoforms have been observed for this gene. [provided by RefSeq, Dec 2010]
Centromere / kinetochore & chromosome segregation	*MCM22*	*CENP-K*	centromere protein K	CENPK is a subunit of a CENPH (MIM 605607)-CENPI (MIM 300065)-associated centromeric complex that targets CENPA (MIM 117139) to centromeres and is required for proper kinetochore function and mitotic progression (Okada et al., 2006 [PubMed 16622420]).[supplied by OMIM, Mar 2008]
Transcription machinery & regulators	*MED9 (CSE2)*	*MED9*	mediator complex subunit 9	The multiprotein Mediator complex is a coactivator required for activation of RNA polymerase II transcription by DNA bound transcription factors. The protein encoded by this gene is thought to be a subunit of the Mediator complex. This gene is located within the Smith-Magenis syndrome region on chromosome 17. [provided by RefSeq, Jul 2008]

Supplemental Table 1 - Functional Grouping and Description of *S. cerevisiae* Genes and Their Human Orthologs

Functional group	Yeast gene	Human ortholog	Human gene name	Function (Alliance of Genome Resources)
Ubiquitin / SUMO system & protein turnover	*MET30*	*FBXW7*	F-box and WD repeat domain containing 7	This gene encodes a member of the F-box protein family which is characterized by an approximately 40 amino acid motif, the F-box. The F-box proteins constitute one of the four subunits of ubiquitin protein ligase complex called SCFs (SKP1-cullin-F-box), which function in phosphorylation-dependent ubiquitination. The F-box proteins are divided into 3 classes: Fbws containing WD-40 domains, Fbls containing leucine-rich repeats, and Fbxs containing either different protein-protein interaction modules or no recognizable motifs. The protein encoded by this gene was previously referred to as FBX30, and belongs to the Fbws class; in addition to an F-box, this protein contains 7 tandem WD40 repeats. This protein binds directly to cyclin E and probably targets cyclin E for ubiquitin-mediated degradation. Mutations in this gene are detected in ovarian and breast cancer cell lines, implicating the gene's potential role in the pathogenesis of human cancers. Multiple transcript variants encoding different isoforms have been found for this gene. [provided by RefSeq, Mar 2012]
Centromere / kinetochore & chromosome segregation	*MIF2*	*CENP-C*	centromere protein C	Centromere protein C 1 is a centromere autoantigen and a component of the inner kinetochore plate. The protein is required for maintaining proper kinetochore size and a timely transition to anaphase. A putative pseudogene exists on chromosome 12. [provided by RefSeq, Jul 2008]
Ubiquitin / SUMO system & protein turnover	*MUB1*	*ZMYND15*	zinc finger MYND-type containing 15	This gene encodes a MYND-containing zinc-binding protein with a nuclear localization sequence. A similar gene in mice has been shown to act as a testis-specific transcriptional repressor by recruiting histone deacetylase enzymes to regulate spatiotemporal expression of many haploid genes. This protein may play an important role in spermatogenesis. Alternative splicing results in multiple transcript variants and protein isoforms. [provided by RefSeq, Jun 2012]
Histone chaperones & assembly regulators	*NAP1*	*NAP1L1*	nucleosome assembly protein 1 like 1	This gene encodes a member of the nucleosome assembly protein (NAP) family. This protein participates in DNA replication and may play a role in modulating chromatin formation and contribute to the regulation of cell proliferation. Alternative splicing results in multiple transcript variants encoding different isoforms; however, not all have been fully described. [provided by RefSeq, Apr 2015]
Centromere / kinetochore & chromosome segregation	*NDC10 (CBF2)*	—	—	—
Centromere / kinetochore & chromosome segregation	*NDC80*	*NDC80*	NDC80 kinetochore complex component	This gene encodes a component of the NDC80 kinetochore complex. The encoded protein consists of an N-terminal microtubule binding domain and a C-terminal coiled-coiled domain that interacts with other components of the complex. This protein functions to organize and stabilize microtubule-kinetochore interactions and is required for proper chromosome segregation. [provided by RefSeq, Oct 2011]
Chromatin remodelers & ATPases	*NHP10*	*TFAM*	transcription factor A, mitochondrial	This gene encodes a key mitochondrial transcription factor containing two high mobility group motifs. The encoded protein also functions in mitochondrial DNA replication and repair. Sequence polymorphisms in this gene are associated with Alzheimer's and Parkinson's diseases. There are pseudogenes for this gene on chromosomes 6, 7, and 11. Alternative splicing results in multiple transcript variants. [provided by RefSeq, Aug 2012]
Ubiquitin / SUMO system & protein turnover	*NPL4*	*NPLOC4*	NPL4 homolog, ubiquitin recognition factor	Predicted to enable ubiquitin binding activity and ubiquitin protein ligase binding activity. Predicted to contribute to K48-linked polyubiquitin modification-dependent protein binding activity and K63-linked polyubiquitin modification-dependent protein binding activity. Involved in negative regulation of RIG-I signaling pathway; negative regulation of type I interferon production; and proteolysis involved in protein catabolic process. Located in nucleus. Part of UFD1-NPL4 complex and VCP-NPL4-UFD1 AAA ATPase complex. [provided by Alliance of Genome Resources, Apr 2025]
Centromere / kinetochore & chromosome segregation	*NUF2*	*NUF2*	NUF2 component of NDC80 kinetochore complex	This gene encodes a protein that is highly similar to yeast Nuf2, a component of a conserved protein complex associated with the centromere. Yeast Nuf2 disappears from the centromere during meiotic prophase when centromeres lose their connection to the spindle pole body, and plays a regulatory role in chromosome segregation. The encoded protein is found to be associated with centromeres of mitotic HeLa cells, which suggests that this protein is a functional homolog of yeast Nuf2. Alternatively spliced transcript variants that encode the same protein have been described. [provided by RefSeq, Jul 2008]
Centromere / kinetochore & chromosome segregation	*OKP1*	*CENP-Q*	centromere protein Q	CENPQ is a subunit of a CENPH (MIM 605607)-CENPI (MIM 300065)-associated centromeric complex that targets CENPA (MIM 117139) to centromeres and is required for proper kinetochore function and mitotic progression (Okada et al., 2006 [PubMed 16622420]).[supplied by OMIM, Mar 2008]
RNA processing & ribosome biogenesis	*PAT1*	*PATL1*	PAT1 homolog 1, processing body mRNA decay factor	Enables poly(G) binding activity and poly(U) RNA binding activity. Involved in P-body assembly and deadenylation-dependent decapping of nuclear-transcribed mRNA. Located in CCR4-NOT complex; P-body; and cytosol. [provided by Alliance of Genome Resources, Apr 2025]

Supplemental Table 1 - Functional Grouping and Description of *S. cerevisiae* Genes and Their Human Orthologs

Functional group	Yeast gene	Human ortholog	Human gene name	Function (Alliance of Genome Resources)
Centromere / kinetochore & chromosome segregation	*PDS1*	*PTTG1*	PTTG1 regulator of sister chromatid separation, securin	The encoded protein is a homolog of yeast securin proteins, which prevent separins from promoting sister chromatid separation. It is an anaphase-promoting complex (APC) substrate that associates with a separin until activation of the APC. The gene product has transforming activity in vitro and tumorigenic activity in vivo, and the gene is highly expressed in various tumors. The gene product contains 2 PXXP motifs, which are required for its transforming and tumorigenic activities, as well as for its stimulation of basic fibroblast growth factor expression. It also contains a destruction box (D box) that is required for its degradation by the APC. The acidic C-terminal region of the encoded protein can act as a transactivation domain. The gene product is mainly a cytosolic protein, although it partially localizes in the nucleus. Three transcript variants encoding the same protein have been found for this gene. [provided by RefSeq, Sep 2013]
DNA damage response & checkpoints	*PIF1*	*PIF1*	PIF1 5'-to-3' DNA helicase	This gene encodes a DNA-dependent adenosine triphosphate (ATP)-metabolizing enzyme that functions as a 5' to 3' DNA helicase. The encoded protein can resolve G-quadruplex structures and RNA-DNA hybrids at the ends of chromosomes. It also prevents telomere elongation by inhibiting the actions of telomerase. Alternative splicing and the use of alternative start codons results in multiple isoforms that are differentially localized to either the mitochondria or the nucleus. [provided by RefSeq, Nov 2013]
Histone chaperones & assembly regulators	*POB3*	*SSRP1*	structure specific recognition protein 1	The protein encoded by this gene is a subunit of a heterodimer that, along with SUPT16H, forms chromatin transcriptional elongation factor FACT. FACT interacts specifically with histones H2A/H2B to effect nucleosome disassembly and transcription elongation. FACT and cisplatin-damaged DNA may be crucial to the anticancer mechanism of cisplatin. This encoded protein contains a high mobility group box which most likely constitutes the structure recognition element for cisplatin-modified DNA. This protein also functions as a co-activator of the transcriptional activator p63. An alternatively spliced transcript variant of this gene has been described, but its full-length nature is not known. [provided by RefSeq, Jul 2008]
Ubiquitin / SUMO system & protein turnover	*PSH1*	*TRIM25*	tripartite motif containing 25	The protein encoded by this gene is a member of the tripartite motif (TRIM) family. The TRIM motif includes three zinc-binding domains, a RING, a B-box type 1 and a B-box type 2, and a coiled-coil region. The protein is an RNA binding protein, functions as a ubiquitin E3 ligase and is involved in multiple cellular processes, including regulation of antiviral innate immunity. [provided by RefSeq, Sep 2021]
RNA processing & ribosome biogenesis	*RAT1*	*XRN2*	5'-3' exoribonuclease 2	This gene encodes a 5'-3' exonuclease that promotes transcription termination at cotranscriptional cleavage sites. Alternative splicing results in multiple transcript variants encoding different isoforms. [provided by RefSeq, Dec 2015]
Membrane trafficking & metabolism	*RCY1*	*EXOC5*	exocyst complex component 5	The protein encoded by this gene is a component of the exocyst complex, a multiple protein complex essential for targeting exocytic vesicles to specific docking sites on the plasma membrane. Though best characterized in yeast, the component proteins and functions of exocyst complex have been demonstrated to be highly conserved in higher eukaryotes. At least eight components of the exocyst complex, including this protein, are found to interact with the actin cytoskeletal remodeling and vesicle transport machinery. The complex is also essential for the biogenesis of epithelial cell surface polarity. [provided by RefSeq, Jul 2008]
Transcription machinery & regulators	*REP1*	—	—	—
Transcription machinery & regulators	*REP2*	—	—	—
RNA processing & ribosome biogenesis	*RIO1*	*RIOK1*	RIO kinase 1	The protein encoded by this gene competes with pICln for inclusion in the protein arginine methyltransferase 5 complex. This complex targets substrates for dimethylation. The encoded protein is essential for the last steps in the maturation of 40S subunits. [provided by RefSeq, Jan 2017]
RNA processing & ribosome biogenesis	*RMT2*	*GAMT*	guanidinoacetate N-methyltransferase	The protein encoded by this gene is a methyltransferase that converts guanidoacetate to creatine, using S-adenosylmethionine as the methyl donor. Defects in this gene have been implicated in neurologic syndromes and muscular hypotonia, probably due to creatine deficiency and accumulation of guanidinoacetate in the brain of affected individuals. Two transcript variants encoding different isoforms have been described for this gene. Pseudogenes of this gene are found on chromosomes 2 and 13. [provided by RefSeq, Feb 2012]

Supplemental Table 1 - Functional Grouping and Description of *S. cerevisiae* Genes and Their Human Orthologs

Functional group	Yeast gene	Human ortholog	Human gene name	Function (Alliance of Genome Resources)
RNA processing & ribosome biogenesis	*RNH1*	*RNASEH1*	ribonuclease H1	This gene encodes an endonuclease that specifically degrades the RNA of RNA-DNA hybrids and plays a key role in DNA replication and repair. Alternate in-frame start codon initiation results in the production of alternate isoforms that are directed to the mitochondria or to the nucleus. The production of the mitochondrial isoform is modulated by an upstream open reading frame (uORF). Mutations in this gene have been found in individuals with progressive external ophthalmoplegia with mitochondrial DNA deletions, autosomal recessive 2. Alternative splicing results in additional coding and non-coding transcript variants. Pseudogenes of this gene have been defined on chromosomes 2 and 17. [provided by RefSeq, Jul 2017]
Transcription machinery & regulators	*RPA43*	*POLR1F*	RNA polymerase I subunit F	Predicted to be involved in transcription elongation by RNA polymerase I. Predicted to act upstream of or within cellular response to leukemia inhibitory factor. Part of RNA polymerase I complex. [provided by Alliance of Genome Resources, Apr 2025]
Histone chaperones & assembly regulators	*RTT106*	—	—	—
Chromatin remodelers & ATPases	*RVB1*	*RUVBL1*	RuvB like AAA ATPase 1	This gene encodes a protein that has both DNA-dependent ATPase and DNA helicase activities and belongs to the ATPases associated with diverse cellular activities (AAA+) protein family. The encoded protein associates with several multisubunit transcriptional complexes and with protein complexes involved in both ATP-dependent remodeling and histone modification. Alternate splicing results in multiple transcript variants. [provided by RefSeq, Jan 2016]
Chromatin remodelers & ATPases	*RVB2*	*RUVBL2*	RuvB like AAA ATPase 2	This gene encodes the second human homologue of the bacterial RuvB gene. Bacterial RuvB protein is a DNA helicase essential for homologous recombination and DNA double-strand break repair. Functional analysis showed that this gene product has both ATPase and DNA helicase activities. This gene is physically linked to the CGB/LHB gene cluster on chromosome 19q13.3, and is very close (55 nt) to the LHB gene, in the opposite orientation. [provided by RefSeq, Jul 2008]
Cell-cycle kinases & regulators	*SAK1*	*CAMKK1*	calcium/calmodulin dependent protein kinase kinase 1	The product of this gene belongs to the Serine/Threonine protein kinase family, and to the Ca(2+)/calmodulin-dependent protein kinase subfamily. This protein plays a role in the calcium/calmodulin-dependent (CaM) kinase cascade. Three transcript variants encoding two distinct isoforms have been identified for this gene. [provided by RefSeq, Jul 2008]
Histone modifiers & silencing enzymes	*SAS2*	*KAT8*	lysine acetyltransferase 8	This gene encodes a member of the MYST histone acetylase protein family. The encoded protein has a characteristic MYST domain containing an acetyl-CoA-binding site, a chromodomain typical of proteins which bind histones, and a C2HC-type zinc finger. Multiple transcript variants encoding different isoforms have been found for this gene. [provided by RefSeq, Feb 2012]
Centromere / kinetochore & chromosome segregation	*SCM3*	— (HJURP as functional equivalent)	Holliday junction recognition protein	Enables histone binding activity and identical protein binding activity. Involved in CENP-A containing chromatin assembly; chromosome segregation; and regulation of protein-containing complex assembly. Located in cytosol; kinetochore; and nuclear lumen. [provided by Alliance of Genome Resources, Apr 2025]
Histone modifiers & silencing enzymes	*SET2*	*SETD2*	SET domain containing 2, histone lysine methyltransferase	Huntington's disease (HD), a neurodegenerative disorder characterized by loss of striatal neurons, is caused by an expansion of a polyglutamine tract in the HD protein huntingtin. This gene encodes a protein belonging to a class of huntingtin interacting proteins characterized by WW motifs. This protein is a histone methyltransferase that is specific for lysine-36 of histone H3, and methylation of this residue is associated with active chromatin. This protein also contains a novel transcriptional activation domain and has been found associated with hyperphosphorylated RNA polymerase II. [provided by RefSeq, Aug 2008]
Transcription machinery & regulators	*SFP1*	*MYC*	MYC proto-oncogene, bHLH transcription factor	This gene is a proto-oncogene and encodes a nuclear phosphoprotein that plays a role in cell cycle progression, apoptosis and cellular transformation. The encoded protein forms a heterodimer with the related transcription factor MAX. This complex binds to the E box DNA consensus sequence and regulates the transcription of specific target genes. Amplification of this gene is frequently observed in numerous human cancers. Translocations involving this gene are associated with Burkitt lymphoma and multiple myeloma in human patients. There is evidence to show that translation initiates both from an upstream, in-frame non-AUG (CUG) and a downstream AUG start site, resulting in the production of two isoforms with distinct N-termini. [provided by RefSeq, Aug 2017]

Supplemental Table 1 - Functional Grouping and Description of *S. cerevisiae* Genes and Their Human Orthologs

Functional group	Yeast gene	Human ortholog	Human gene name	Function (Alliance of Genome Resources)
Histone modifiers & silencing enzymes	*SIR2*	*SIRT1*	sirtuin 1	This gene encodes a member of the sirtuin family of proteins, homologs to the yeast Sir2 protein. Members of the sirtuin family are characterized by a sirtuin core domain and grouped into four classes. The functions of human sirtuins have not yet been determined; however, yeast sirtuin proteins are known to regulate epigenetic gene silencing and suppress recombination of rDNA. Studies suggest that the human sirtuins may function as intracellular regulatory proteins with mono-ADP-ribosyltransferase activity. The protein encoded by this gene is included in class I of the sirtuin family. Alternative splicing results in multiple transcript variants. [provided by RefSeq, Dec 2008]
Ubiquitin / SUMO system & protein turnover	*SIZ1*	*PIAS1*	protein inhibitor of activated STAT 1	This gene encodes a member of the protein inhibitor of activated STAT (PIAS) family. PIAS proteins function as SUMO E3 ligases and play important roles in many cellular processes by mediating the sumoylation of target proteins. This protein plays a central role as a transcriptional coregulator of numerous cellular pathways includign the STAT1 and nuclear factor kappaB pathways. Alternate splicing results in multiple transcript variants. [provided by RefSeq, Mar 2016]
Ubiquitin / SUMO system & protein turnover	*SIZ2 (NFI1)*	*PIAS1*	protein inhibitor of activated STAT 1	This gene encodes a member of the protein inhibitor of activated STAT (PIAS) family. PIAS proteins function as SUMO E3 ligases and play important roles in many cellular processes by mediating the sumoylation of target proteins. This protein plays a central role as a transcriptional coregulator of numerous cellular pathways includign the STAT1 and nuclear factor kappaB pathways. Alternate splicing results in multiple transcript variants. [provided by RefSeq, Mar 2016]
Ubiquitin / SUMO system & protein turnover	*SKP1*	*SKP1*	S-phase kinase associated protein 1	This gene encodes a component of SCF complexes, which are composed of this protein, cullin 1, a ring-box protein, and one member of the F-box family of proteins. This protein binds directly to the F-box motif found in F-box proteins. SCF complexes are involved in the regulated ubiquitination of specific protein substrates, which targets them for degradation by the proteosome. Specific F-box proteins recognize different target protein(s), and many specific SCF substrates have been identified including regulators of cell cycle progression and development. Studies have also characterized the protein as an RNA polymerase II elongation factor. Alternative splicing of this gene results in two transcript variants. A related pseudogene has been identified on chromosome 7. [provided by RefSeq, Jul 2008]
Centromere / kinetochore & chromosome segregation	*SLI15*	*INCENP*	inner centromere protein	In mammalian cells, 2 broad groups of centromere-interacting proteins have been described: constitutively binding centromere proteins and 'passenger,' or transiently interacting, proteins (reviewed by Choo, 1997). The constitutive proteins include CENPA (centromere protein A; MIM 117139), CENPB (MIM 117140), CENPC1 (MIM 117141), and CENPD (MIM 117142). The term 'passenger proteins' encompasses a broad collection of proteins that localize to the centromere during specific stages of the cell cycle (Earnshaw and Mackay, 1994 [PubMed 8088460]). These include CENPE (MIM 117143); MCAK (MIM 604538); KID (MIM 603213); cytoplasmic dynein (e.g., MIM 600112); CliPs (e.g., MIM 179838); and CENPF/mitosin (MIM 600236). The inner centromere proteins (INCENPs) (Earnshaw and Cooke, 1991 [PubMed 1860899]), the initial members of the passenger protein group, display a broad localization along chromosomes in the early stages of mitosis but gradually become concentrated at centromeres as the cell cycle progresses into mid-metaphase. During telophase, the proteins are located within the midbody in the intercellular bridge, where they are discarded after cytokinesis (Cutts et al., 1999 [PubMed 10369859]).[supplied by OMIM, Mar 2008]
Ubiquitin / SUMO system & protein turnover	*SLX5*	*RNF4*	ring finger protein 4	The protein encoded by this gene contains a RING finger motif and acts as a transcription regulator. This protein has been shown to interact with, and inhibit the activity of, TRPS1, a transcription suppressor of GATA-mediated transcription. Transcription repressor ZNF278/PATZ is found to interact with this protein, and thus reduce the enhancement of androgen receptor-dependent transcription mediated by this protein. Studies of the mouse and rat counterparts suggested a role of this protein in spermatogenesis. A pseudogene of this gene is found on chromosome 1.[provided by RefSeq, Jul 2010]
Ubiquitin / SUMO system & protein turnover	*SLX8*			
Cell-cycle kinases & regulators	*SNF1*	*PRKAA1*	protein kinase AMP-activated catalytic subunit alpha 1	The protein encoded by this gene belongs to the ser/thr protein kinase family. It is the catalytic subunit of the 5'-prime-AMP-activated protein kinase (AMPK). AMPK is a cellular energy sensor conserved in all eukaryotic cells. The kinase activity of AMPK is activated by the stimuli that increase the cellular AMP/ATP ratio. AMPK regulates the activities of a number of key metabolic enzymes through phosphorylation. It protects cells from stresses that cause ATP depletion by switching off ATP-consuming biosynthetic pathways. Alternatively spliced transcript variants encoding distinct isoforms have been observed. [provided by RefSeq, Jul 2008]

Supplemental Table 1 - Functional Grouping and Description of *S. cerevisiae* Genes and Their Human Orthologs

Functional group	Yeast gene	Human ortholog	Human gene name	Function (Alliance of Genome Resources)
Chromatin remodelers & ATPases	*SNF2*	*SMARCA4*	SWI/SNF related BAF chromatin remodeling complex subunit ATPase 4	The protein encoded by this gene is a member of the SWI/SNF family of proteins and is similar to the brahma protein of Drosophila. Members of this family have helicase and ATPase activities and are thought to regulate transcription of certain genes by altering the chromatin structure around those genes. The encoded protein is part of the large ATP-dependent chromatin remodeling complex SNF/SWI, which is required for transcriptional activation of genes normally repressed by chromatin. In addition, this protein can bind BRCA1, as well as regulate the expression of the tumorigenic protein CD44. Mutations in this gene cause rhabdoid tumor predisposition syndrome type 2. Multiple transcript variants encoding different isoforms have been found for this gene. [provided by RefSeq, May 2012]
Cell-cycle kinases & regulators	*SNF4*	*PRKAG1*	protein kinase AMP-activated non-catalytic subunit gamma 1	The protein encoded by this gene is a regulatory subunit of the AMP-activated protein kinase (AMPK). AMPK is a heterotrimer consisting of an alpha catalytic subunit, and non-catalytic beta and gamma subunits. AMPK is an important energy-sensing enzyme that monitors cellular energy status. In response to cellular metabolic stresses, AMPK is activated, and thus phosphorylates and inactivates acetyl-CoA carboxylase (ACC) and beta-hydroxy beta-methylglutaryl-CoA reductase (HMGCR), key enzymes involved in regulating de novo biosynthesis of fatty acid and cholesterol. This subunit is one of the gamma regulatory subunits of AMPK. Alternatively spliced transcript variants encoding distinct isoforms have been observed. [provided by RefSeq, Jul 2008]
Centromere / kinetochore & chromosome segregation	*SPC24*	*SPC24*	SPC24 component of NDC80 kinetochore complex	Predicted to contribute to microtubule binding activity. Involved in attachment of spindle microtubules to kinetochore. Located in nucleolus and nucleoplasm. Part of Ndc80 complex. [provided by Alliance of Genome Resources, Apr 2025]
Centromere / kinetochore & chromosome segregation	*SPC25*	*SPC25*	SPC25 component of NDC80 kinetochore complex	This gene encodes a protein that may be involved in kinetochore-microtubule interaction and spindle checkpoint activity. [provided by RefSeq, Jul 2008]
Centromere / kinetochore & chromosome segregation	*SPC34*	subunit of Ska complex	—	—
Cell-cycle kinases & regulators	*SPS1*	*STK26*	serine/threonine kinase 26	The product of this gene is a member of the GCK group III family of kinases, which are a subset of the Ste20-like kinases. The encoded protein contains an amino-terminal kinase domain, and a carboxy-terminal regulatory domain that mediates homodimerization. The protein kinase localizes to the Golgi apparatus and is specifically activated by binding to the Golgi matrix protein GM130. It is also cleaved by caspase-3 in vitro, and may function in the apoptotic pathway. Several alternatively spliced transcript variants of this gene have been described, but the full-length nature of some of these variants has not been determined. [provided by RefSeq, Jul 2008]
Histone chaperones & assembly regulators	*SPT16*	*SUPT16H*	SPT16 homolog, facilitates chromatin remodeling subunit	Transcription of protein-coding genes can be reconstituted on naked DNA with only the general transcription factors and RNA polymerase II. However, this minimal system cannot transcribe DNA packaged into chromatin, indicating that accessory factors may facilitate access to DNA. One such factor, FACT (facilitates chromatin transcription), interacts specifically with histones H2A/H2B to effect nucleosome disassembly and transcription elongation. FACT is composed of an 80 kDa subunit and a 140 kDa subunit; this gene encodes the 140 kDa subunit. [provided by RefSeq, Feb 2009]
Transcription machinery & regulators	*SPT4*	*SUPT4H1*	SPT4 homolog, DSIF elongation factor subunit	This gene encodes the small subunit of DRB (5,6-dichloro-1-beta-d-ribofuranosylbenzimidazole) sensitivity-inducing factor (DSIF) complex, which regulates mRNA processing and transcription elongation by RNA polymerase II. The encoded protein is localized to the nucleus and interacts with the large subunit (SUPT5H) to form the DSIF complex. Related pseudogenes have been identified on chromosomes 2 and 12. Alternatively spliced transcript variants have been found for this gene. [provided by RefSeq, Nov 2012]
Transcription machinery & regulators	*SPT5*	*SUPT5H*	SPT5 homolog, DSIF elongation factor subunit	Enables enzyme binding activity and protein heterodimerization activity. Involved in positive regulation of macroautophagy; regulation of DNA-templated transcription; and transcription elongation by RNA polymerase II. Located in nucleoplasm. Part of DSIF complex. [provided by Alliance of Genome Resources, Apr 2025]
Chromatin remodelers & ATPases	*STH1*	*SMARCA4*	SWI/SNF related BAF chromatin remodeling complex subunit ATPase 4	The protein encoded by this gene is a member of the SWI/SNF family of proteins and is similar to the brahma protein of Drosophila. Members of this family have helicase and ATPase activities and are thought to regulate transcription of certain genes by altering the chromatin structure around those genes. The encoded protein is part of the large ATP-dependent chromatin remodeling complex SNF/SWI, which is required for transcriptional activation of genes normally repressed by chromatin. In addition, this protein can bind BRCA1, as well as regulate the expression of the tumorigenic protein CD44. Mutations in this gene cause rhabdoid tumor predisposition syndrome type 2. Multiple transcript variants encoding different isoforms have been found for this gene. [provided by RefSeq, May 2012]

Supplemental Table 1 - Functional Grouping and Description of *S. cerevisiae* Genes and Their Human Orthologs

Functional group	Yeast gene	Human ortholog	Human gene name	Function (Alliance of Genome Resources)
Membrane trafficking & metabolism	*STP1*	*PRDM1*	PR/SET domain 1	This gene encodes a protein that acts as a repressor of beta-interferon gene expression. The protein binds specifically to the PRDI (positive regulatory domain I element) of the beta-IFN gene promoter. Transcription of this gene increases upon virus induction. Two alternatively spliced transcript variants that encode different isoforms have been reported. [provided by RefSeq, Jul 2008]
Chromatin remodelers & ATPases	*SWR1*	*SRCAP*	Snf2 related CREBBP activator protein	This gene encodes the core catalytic component of the multiprotein chromatin-remodeling SRCAP complex. The encoded protein is an ATPase that is necessary for the incorporation of the histone variant H2A.Z into nucleosomes. It can function as a transcriptional activator in Notch-mediated, CREB-mediated and steroid receptor-mediated transcription. Mutations in this gene cause Floating-Harbor syndrome, a rare disorder characterized by short stature, language deficits and dysmorphic facial features. [provided by RefSeq, Feb 2012]
RNA processing & ribosome biogenesis	*TRF4 (PAP2)*	*TENT4A*	terminal nucleotidyl-transferase 4A	The protein encoded by this gene is a DNA polymerase that is likely involved in DNA repair. In addition, the encoded protein may be required for sister chromatid adhesion. Alternatively spliced transcript variants that encode different isoforms have been described. [provided by RefSeq, Jan 2010]
Ubiquitin / SUMO system & protein turnover	*UBC4*	*UBE2D2*	ubiquitin conjugating enzyme E2 D2	Regulated degradation of misfolded, damaged or short-lived proteins in eukaryotes occurs via the ubiquitin (Ub)-proteasome system (UPS). An integral part of the UPS system is the ubiquitination of target proteins and covalent linkage of Ub-containing proteins to form polymeric chains, marking them as targets for 26S proteasome-mediated degradation. Ubiquitination of proteins is mediated by a cascade of enzymes which includes E1 (ubiquitin activating), E2 (ubiquitin conjugating), and E3 (ubiquitin ligases) enzymes. This gene encodes a member of the E2 enzyme family. Substrates of this enzyme include the tumor suppressor protein p53 and peroxisomal biogenesis factor 5 (PEX5). Alternative splicing results in multiple transcript variants of this gene. [provided by RefSeq, May 2013]
Ubiquitin / SUMO system & protein turnover	*UBC9*	*UBE2I*	ubiquitin conjugating enzyme E2 I	The modification of proteins with ubiquitin is an important cellular mechanism for targeting abnormal or short-lived proteins for degradation. Ubiquitination involves at least three classes of enzymes: ubiquitin-activating enzymes, or E1s, ubiquitin-conjugating enzymes, or E2s, and ubiquitin-protein ligases, or E3s. This gene encodes a member of the E2 ubiquitin-conjugating enzyme family. Four alternatively spliced transcript variants encoding the same protein have been found for this gene. [provided by RefSeq, Jul 2008]
Ubiquitin / SUMO system & protein turnover	*UBR1*	*UBR1*	ubiquitin protein ligase E3 component n-recognin 1	The N-end rule pathway is one proteolytic pathway of the ubiquitin system. The recognition component of this pathway, encoded by this gene, binds to a destabilizing N-terminal residue of a substrate protein and participates in the formation of a substrate-linked multiubiquitin chain. This leads to the eventual degradation of the substrate protein. The protein described in this record has a RING-type zinc finger and a UBR-type zinc finger. Mutations in this gene have been associated with Johanson-Blizzard syndrome. [provided by RefSeq, Jul 2008]
Ubiquitin / SUMO system & protein turnover	*UBR2*			
Ubiquitin / SUMO system & protein turnover	*UFD1*	*UFD1*	ubiquitin recognition factor in ER associated degradation 1	The protein encoded by this gene forms a complex with two other proteins, nuclear protein localization-4 and valosin-containing protein, and this complex is necessary for the degradation of ubiquitinated proteins. In addition, this complex controls the disassembly of the mitotic spindle and the formation of a closed nuclear envelope after mitosis. Mutations in this gene have been associated with Catch 22 syndrome as well as cardiac and craniofacial defects. Alternative splicing results in multiple transcript variants encoding different isoforms. A related pseudogene has been identified on chromosome 18. [provided by RefSeq, Jun 2009]
Membrane trafficking & metabolism	*UPA1*	*SPOUT1*	SPOUT domain containing methyltransferase 1	Enables miRNA binding activity. Involved in maintenance of centrosome location and miRNA processing. Acts upstream of with a positive effect on post-transcriptional regulation of gene expression. Located in kinetochore; mitotic spindle; and spindle pole centrosome. [provided by Alliance of Genome Resources, Apr 2025]
Chromatin remodelers & ATPases	*YTA7*	*ATAD2*	ATPase family AAA domain containing 2	A large family of ATPases has been described, whose key feature is that they share a conserved region of about 220 amino acids that contains an ATP-binding site. The proteins that belong to this family either contain one or two AAA (ATPases Associated with diverse cellular Activities) domains. AAA family proteins often perform chaperone-like functions that assist in the assembly, operation, or disassembly of protein complexes. The protein encoded by this gene contains two AAA domains, as well as a bromodomain. [provided by RefSeq, Jul 2008]
